# Rational optimization of a transcription factor activation domain inhibitor

**DOI:** 10.1038/s41594-023-01159-5

**Published:** 2023-12-04

**Authors:** Shaon Basu, Paula Martínez-Cristóbal, Marta Frigolé-Vivas, Mireia Pesarrodona, Michael Lewis, Elzbieta Szulc, C. Adriana Bañuelos, Carolina Sánchez-Zarzalejo, Stasė Bielskutė, Jiaqi Zhu, Karina Pombo-García, Carla Garcia-Cabau, Levente Zodi, Hannes Dockx, Jordann Smak, Harpreet Kaur, Cristina Batlle, Borja Mateos, Mateusz Biesaga, Albert Escobedo, Lídia Bardia, Xavier Verdaguer, Alessandro Ruffoni, Nasrin R. Mawji, Jun Wang, Jon K. Obst, Teresa Tam, Isabelle Brun-Heath, Salvador Ventura, David Meierhofer, Jesús García, Paul Robustelli, Travis H. Stracker, Marianne D. Sadar, Antoni Riera, Denes Hnisz, Xavier Salvatella

**Affiliations:** 1 Department of Genome Regulation, Max Planck Institute for Molecular Genetics, Berlin, Germany; 2 Institute for Research in Biomedicine (IRB Barcelona), The Barcelona Institute of Science and Technology, Barcelona, Spain; 3 Genome Sciences, BC Cancer and Department of Pathology and Laboratory Medicine, University of British Columbia, Vancouver, Canada; 4 Dartmouth College, Department of Chemistry, Hanover, NH USA; 5 Max Planck Institute of Molecular Cell Biology and Genetics, Dresden, Germany; 6 Nuage Therapeutics, Barcelona, Spain; 7 Radiation Oncology Branch, Center for Cancer Research, National Cancer Institute, NIH, Bethesda, MD USA; 8 Institut de Biotecnologia i Biomedicina and Departament de Bioquímica i Biologia Molecular, Universitat Autònoma de Barcelona, Bellaterra, Spain; 9 Departament de Química Inorgànica i Orgànica, Universitat de Barcelona, Barcelona, Spain; 10 Max Planck Institute for Molecular Genetics, Mass Spectrometry Facility, Berlin, Germany; 11 ICREA, Barcelona, Spain

**Keywords:** Intrinsically disordered proteins, Drug discovery, Protein aggregation

## Abstract

Transcription factors are among the most attractive therapeutic targets but are considered largely ‘undruggable’ in part due to the intrinsically disordered nature of their activation domains. Here we show that the aromatic character of the activation domain of the androgen receptor, a therapeutic target for castration-resistant prostate cancer, is key for its activity as transcription factor, allowing it to translocate to the nucleus and partition into transcriptional condensates upon activation by androgens. On the basis of our understanding of the interactions stabilizing such condensates and of the structure that the domain adopts upon condensation, we optimized the structure of a small-molecule inhibitor previously identified by phenotypic screening. The optimized compounds had more affinity for their target, inhibited androgen-receptor-dependent transcriptional programs, and had an antitumorigenic effect in models of castration-resistant prostate cancer in cells and in vivo. These results suggest that it is possible to rationally optimize, and potentially even to design, small molecules that target the activation domains of oncogenic transcription factors.

## Abstract (web summary)

Transcription factors are rich in intrinsic disorder and therefore hard to drug. The authors improve an experimental drug for castration-resistant prostate cancer by learning how the activation domain of the androgen receptor activates transcription.

## Main

The genes encoding transcription factors (TFs) are frequently mutated or dysregulated in cancer, and TFs are coveted targets in oncology^1,2^. For example, *TP53*, the most frequently mutated gene in cancer, and *MYC*, the most frequently overexpressed gene in cancer, encode TFs^3^. The rewiring of transcriptional programs is a hallmark of cancer, and oncogenic transcriptional programs of numerous tumor types depend on small subsets of specific TFs^2,4^. Despite their appeal, TFs are considered largely ‘undruggable’ because of the intrinsic disorder of their protein regions that are essential for transcriptional activity, rendering them challenging targets for structure-based drug discovery^5,6^.

Nuclear hormone receptors, for example the androgen receptor (AR), are TFs that contain a structured ligand-binding domain (LBD), and anti-androgens targeting the LBD are a first-line therapy for the treatment of AR-driven prostate cancer^7,8^. However, approximately 20% of people with prostate cancer progress to castration-resistant prostate cancer (CRPC), a lethal disease that is associated with the emergence of constitutively active AR splice variants. Such splice variants lack a LBD and consist of only a DNA-binding domain (DBD) and an intrinsically disordered activation domain (AD), rendering them insensitive to LBD-targeting anti-androgens^9–12^. Insights into how the ADs of oncogenes function could thus facilitate the development of therapeutic approaches for some of the most lethal cancers.

Recent studies have suggested that IDRs in many cellular proteins mediate liquid–liquid phase separation in vitro and the partitioning of proteins into biomolecular condensates in cells^13,14^. Essentially all human TFs, including AR, contain an IDR, and these regions have recently been shown to contribute to the formation of TF condensates and the partitioning of TFs into heterotypic condensates with transcriptional effectors such as the co-activator Mediator (MED-1) or RNA polymerase II (RNAPII)^15–19^. The molecular basis of TF condensation has been dissected for a small number of TFs and, in all cases, substitutions of amino acids in the IDRs that altered phase separation also altered transcriptional activity^15,20–22^. On the basis of these findings, we hypothesized that gaining insights into the molecular basis of the phase-separation capacity encoded in the IDRs of oncogenic TFs could be exploited to develop small molecules that alter its activity.

To investigate this, we chose to study the sequence and structural determinants of AR phase separation. We discovered that this process is required for nuclear translocation and transactivation. The phase transition is driven by interactions between aromatic residues, distributed throughout the sequence of the AD but particularly concentrated near the C terminus. This region includes a subdomain of the AD that has high helical propensity (transactivation unit 5, Tau-5). Tau-5, which plays a key role in transactivation by the splice variants associated with CRPC, harbors the binding site of EPI-001 (ref. ^23^), a small-molecular inhibitor of the AR AD discovered by phenotypic screening^24^, a derivative of which is being investigated in clinical trials for CRPC (NCT04421222, NCT05075577). On the basis of how this small molecule interacts with Tau-5, we introduced changes in its chemical structure that have led to substantially improved potency in cells and in human xenografts models of CRPC.

## Results

### AR phase separation is driven by tyrosine residues in the AD

**Fig. 1:**
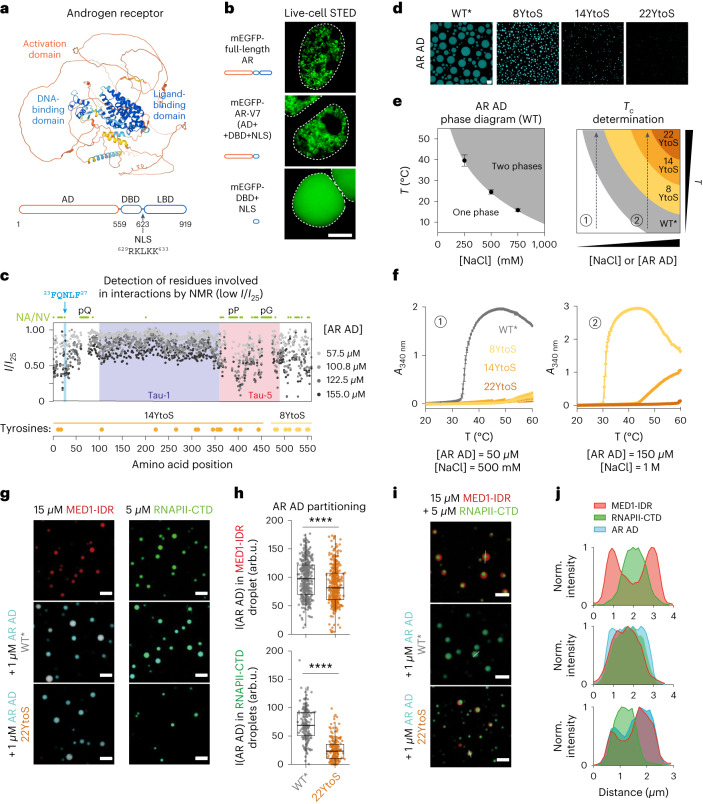
AR phase separation is driven by tyrosine residues in the AD. **a**, Predicted structure of AR, colored by structure-prediction confidence from high (blue) to low (yellow). The domains and the native NLS are highlighted. **b**, Live-cell STED imaging of representative (*n* > 3) HEK293T cells transfected with AR constructs tagged with mEGFP. Cells were imaged after treatment with 10 nM DHT for 4 h. Scale bar, 5 μm. The dashed line indicates the nuclear periphery. **c**, Intensity of AR AD NMR resonances at different concentrations, relative to the intensity at 25 μM. The positions of Tau-1, Tau-5 and ^23^FQNLF^27^ are highlighted. Green circles indicate residues that were not visible (NV) or not assigned (NA), including residues in polyglutamine (pQ), polyproline (pP) and polyglycine (pG) tracts. Yellow and orange circles represent the positions of tyrosine residues substituted by serines in 8YtoS and 14YtoS; all tyrosine residues were substituted in 22YtoS. **d**, Fluorescence microscopy images of 40 µM AR-AD droplets (WT* and mutants) at 1 M NaCl. Scale bar, 10 μm. **e**, Scheme of the phase diagram of the AR AD and of how *T*_c_ measurements at different solution conditions allow the phase separation capacity of the mutants to be ranked. **f**, *T*_c_ measurements of AR AD (WT* and the tyrosine to serine mutants), as mean ± s.d. of three independent samples, at two different solution conditions. **g**, Representative merged confocal images of 15 µM MED1-IDR and 5 µM RNAPII-CTD droplets at 20 mM NaCl or 50 mM NaCl, respectively, and 10% ficoll before and after addition of 1 µM AR AD (WT* or 22YtoS). Scale bars, 5 μm. **h**, Quantification of AR AD partitioning in MED1-IDR (top) and RNAPII-CTD droplets (bottom), by measuring AR AD fluorescence intensity (*I*(AR AD)). Boxes show the mean and the quartiles of all droplets, represented as colored dots from three replicated images. arb.u., arbitrary units. **i**, Representative (*n* > 3) merged confocal images of MED1-IDR and RNAPII-CTD droplets obtained in 125 mM NaCl and 10% ficoll with and without the addition of 1 µM AR AD (WT* or 22YtoS). Scale bar, 5 μm. **j**, Normalized intensity plot of cross-sections from the images shown in **i**. Source data

**Extended Data Fig. 1:**
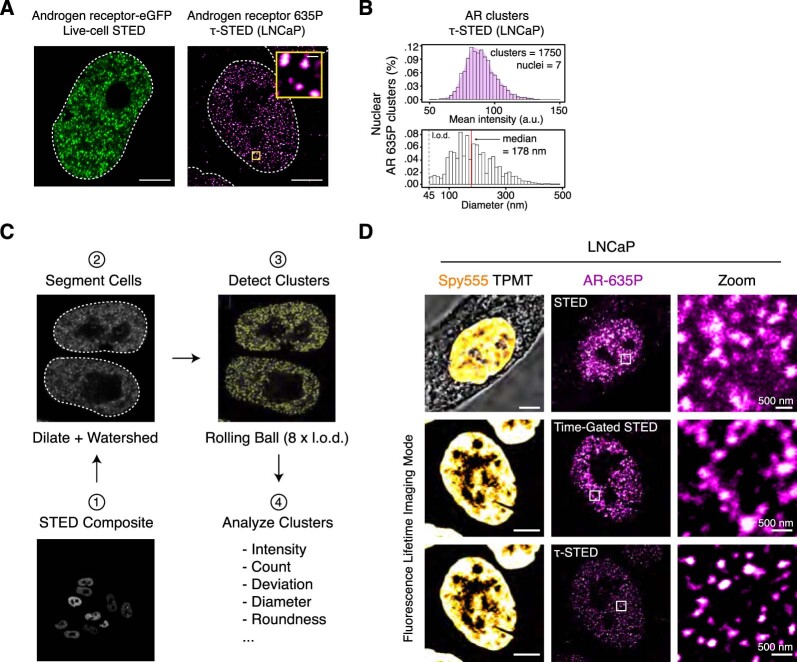
Characterization of AR condensates in cells using high resolution microscopy. **a**) (left) Live-cell stimulated emission depletion (STED) imaging of a HeLa cell nucleus expressing AR-eGFP, treated with 1 nM DHT for 4 h (right) τ-STED imaging of endogenous AR in fixed human prostate adenocarcinoma (LNCaP) cells. Large scale bars: 5 μm. Scale bar in τ-STED inset: 300 nm. Dashed line indicates the nuclear periphery. **b**) (top) Quantification of τ-STED intensity signal and (bottom) diameter of endogenous AR clusters in LNCaP cells (1750 AR clusters detected across 7 LNCaP nuclei imaged with same fluorescence time gating). L.o.d indicates the limit of detection. Densitymax diameter (bin with highest density of AR clusters in the distribution of all detected AR clusters): 123 nm, median diameter: 178 nm. **c**) Quantification pipeline used to analyze STED image composites, showing segmentation of cells and detection of clusters using rolling ball background subtraction adjusted to 8 x the resolving capacity of the image (48 nm pixel^-1^ for TauSTED imaging of LNCaP cells). **d**) Representative (*n* > 3) STED (top row) and FLIM STED images showing AR clusters in LNCaP nuclei before and after τ-STED deconvolution (middle and bottom row). Left column shows LNCaP nuclear counterstain using Spy555-DNA stain. Scale bar: 5 μm. Right panels show zoom-ins corresponding to intra-nuclear regions indicated by white boxes on panels in the central column. Scale bar: 500 nm. Source data

**Extended Data Fig. 2:**
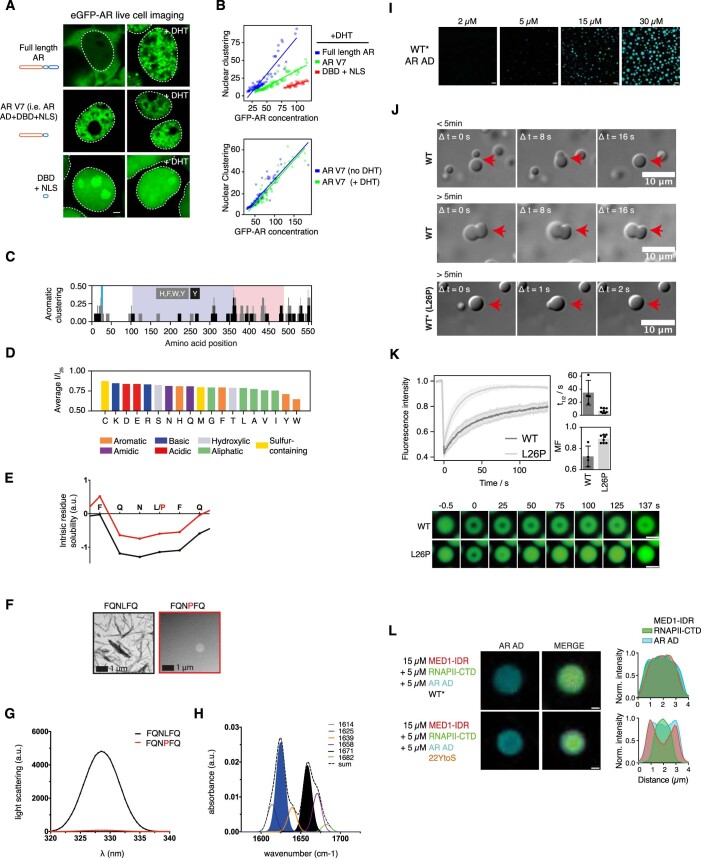
AR phase separation is driven by tyrosine residues in the AD. **a**) Live-cell confocal imaging of constructs in HEK293T cells after treatment with vehicle or 10 nM DHT for 4 h. Scale bar: 3 µm. Dashed lines indicate nuclear periphery. **b**) Quantification of data in panel A. Y-axis indicates s.d. and x-axis indicates mean intensity of pixels in the corresponding nucleus. Each dot represents measurements from an individual cell, and lines represent standard regression fits to the corresponding data spread (*n* = 2). **c**) Distribution of aromatic and tyrosine residues, clustered using a 9 residue window, where the shaded areas correspond to those represented in Fig. 1c. **d**) Average intensity of the resonances at different concentrations, relative to their intensity at 25 μM, grouped by residue type. **e**) Solubility predicted by CamSol for peptides FQNLFQ (black line) and FQNPFQ (red line)^99^. **f**) Representative (*n* > 3) TEM micrographs of peptides FQNLFQ and FQNPFQ after an overnight incubation. **g**) Synchronous light scattering of peptides FQNLFQ (black line) and FQNPFQ (red line) after an overnight incubation. **h**) FT-IR absorbance spectrum in the amide I region (dashed line) of the aggregates formed by the FQNLFQ peptide. The blue shaded area indicates the contribution of the intermolecular β-sheet signal. **i**) Fluorescence microscopy images of AR AD (WT*) droplets in 20 mM sodium phosphate, 1 mM TCEP pH 7.4 with 150 mM NaCl and 10% ficoll. Scale bar: 10 µm. **j**) DIC images showing fusion events of 50 μM AR AD WT or WT* samples at 500 mM NaCl at times before and/or after 5 min from sample preparation. **k**) Fluorescence intensity recovery curves shown as average and s.d. (*n* = 3 independent samples) and quantification of the recovery half-time and mobile fraction (average ± s.d., *n* = 4 and *n* = 8 droplets for WT and L26P (WT*) respectively) and representative confocal microscopy images. **l**) (Left) Droplets formed by the indicated proteins and signals of the AR AD channel and merged channel. AR AD proteins were used concentrations 5 times higher than in Fig. 1i. Scale bar: 1 µm. (Right) the representative droplet’s cross-section intensity profile. Source data

AR forms mesoscale nuclear ‘speckles,’ but their properties have been elusive because of their small size and the nuclear shuttling of the receptor^17–19,25,26^. Using live-cell and fixed imaging, we confirmed that hormone-stimulated endogenous and transgenic AR form clusters (Extended Data Fig. 1). To identify the molecular basis of cluster formation, we studied the clusters formed by AR variants. Full-length AR contains an intrinsically disordered AD, DBD and a C-terminal LBD (Fig. 1a). We found that, in transiently transfected HEK293T cells, full-length AR and the AR-V7 splice variant, which contain the AD and DBD, formed nuclear clusters, but the DBD alone did not (Fig. 1b); as expected, AR-V7 formed nuclear clusters even in the absence of hormone (Extended Data Fig. 2a,b). Of note, higher expression of AR and AR-V7 increased nuclear clustering in HEK293T cells (Extended Data Fig. 2a,b), consistent with the notion that cluster formation involves phase separation and is driven by the AR AD.

To identify the residues of the AR AD that drive cluster formation, we used solution nuclear magnetic resonance (NMR). This technique provides residue-specific information in the absence of structure and is thus well-suited to studying intrinsically disordered proteins^27^. An analysis of the ^1^H–^15^N correlation spectrum of purified AR AD revealed that the intensity of the signals of many residues was low, especially when the AR AD was present at high concentrations, suggesting that these residues are involved in transient intermolecular interactions^28^. We analyzed the decrease in signal intensity as a function of position and residue type, which revealed that the residues involved in such interactions are hydrophobic, and many of them are aromatic (Fig. 1c and Extended Data Fig. 2c,d). These residues are clustered around the ^23^FQNLF^27^ motif^29 and^, especially, in Tau-5 (Extended Data Fig. 2c).

We recently reported that Hsp70 binding to the ^23^FQNLF^27^ motif of the AR AD increases its solubility in vitro^30^. To avoid interference from this aggregation-prone motif in our ability to study AR AD in vitro, we introduced a substitution in the motif (L26P) that decreases its propensity to aggregate (Extended Data Fig. 2e–h). The AR AD formed droplets in vitro in a concentration-dependent manner (Extended Data Fig. 2i) and the substitution increased the kinetic stability of the droplets (Extended Data Fig. 2j) as well as the rate and degree of recovery in fluorescence recovery after photobleaching experiments (Extended Data Fig. 2k). The AR AD containing substitution L26P, which was introduced only in the experiments performed in vitro, is referred to as wild type* (WT*) throughout the study.

To test the contribution of aromatic residues to phase separation, we measured how decreasing the aromatic character of the AR AD affects its cloud point (*T*_c_) in vitro^18,31^. We substituted tyrosine residues, the most abundant aromatic amino acid in the AR AD, by serines, thus generating three mutants: 8YtoS, in which the 8 tyrosines closest to the DBD were substituted; 14YtoS, in which the other 14 tyrosines were substituted; and 22YtoS, in which all tyrosines were substituted (Fig. 1c). The substitution of tyrosines by serines led to a reduction in droplet formation (Fig. 1d): *T*_c_ measurements revealed that phase separation of the AR AD occurred at high temperature and ionic strength (Fig. 1e) in the lower critical solution temperature (LCST) regime; therefore, an elevated *T*_c_ is indicative of a reduction in phase separation capacity. We found that none of the YtoS mutants phase-separated at temperatures lower than 60 °C under conditions in which *T*_c_ = 34 °C for WT* AR AD (Fig. 1e,f). To resolve the phase separation capacity of the mutants, we increased protein concentration and ionic strength: we observed that the *T*_c_ measurements of 8YtoS and 14YtoS were 31 °C and 48 °C, respectively; the 22YtoS mutant did not phase separate (Fig. 1f).

Substitution of aromatic residues also compromised the partitioning of the AR AD into heterotypic condensates with transcriptional effector partners. We incubated AR AD proteins with preassembled droplets formed by purified recombinant MED1 IDR, an in vitro model of Mediator condensates^32^, and droplets formed by purified recombinant RNAPII C-terminal domain (CTD), an in vitro model for RNAPII condensates^33^. WT* AR AD partitioned into both MED1 IDR and RNAPII CTD droplets, whereas the partitioning was reduced by the 22YtoS AR AD mutant (Fig. 1g,h). We modeled heterotypic condensation by mixing MED1 IDR, RNAPII CTD and AR AD proteins. To our surprise, MED1 IDR and RNAPII CTD formed biphasic droplets in which the RNAPII CTD was segregated from the MED1 IDR within the MED1 IDR droplets (Fig. 1i). The addition of 1 µM WT* AR AD caused the biphasic droplets to blend into a single phase, in which the three components were distributed homogeneously (Fig. 1i,j). This relied on the aromatic character of the AR AD, because the addition of 1 µM 22YtoS led to preferential partitioning into the MED1-IDR liquid phase under the same experimental conditions (Fig. 1i,j and Extended Data Fig. 2l). We attempted to express and purify AR in mammalian cells to study the effect of the YtoS alterations in the full-length receptor, but we could not obtain sufficient amounts of high-quality samples for in vitro experiments.

### AR phase separation is associated with key AR functions

**Fig. 2:**
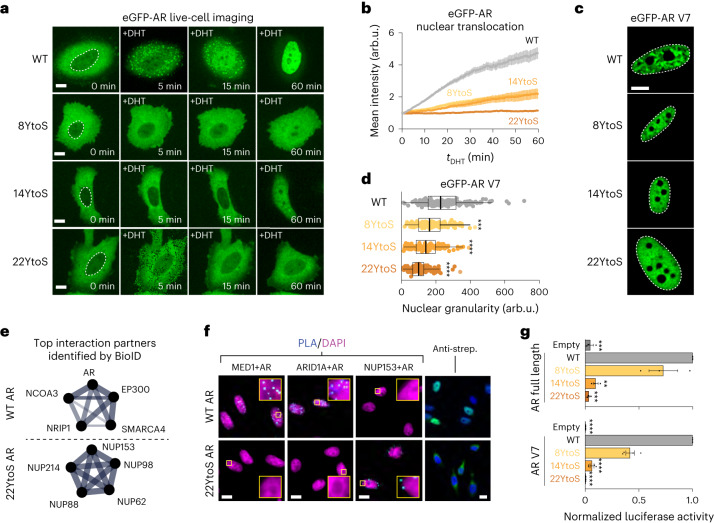
AR phase separation is associated with nuclear translocation and transactivation. **a**, Fluorescence images from live-cell time-lapse videos of PC3 cells expressing eGFP-AR or the indicated mutants. Scale bar, 10 μm. **b**, Quantification of eGFP-AR relative nuclear localization for the cells in **a**, as a function of time elapsed since the addition of 1 nM DHT (*t*_DHT_). Error bars represent the s.d. of *n* ≥ 15 cells per time point. **c**, Representative images (*n* > 3) of live PC3 nuclei expressing eGFP-AR-V7 WT or a Tyr to Ser mutant. Scale bar, 5 μm. **d**, Quantification of the nuclear granularity for the cells in **c**; each dot represents one nucleus, boxes show the mean of the quartiles of all cells and *P* values were calculated using a Dunnett’s multiple-comparison test against the WT (*n* ≥ 150 cells per condition). **e**, Selected Gene Ontology (GO) molecular function networks enriched in the top 75 most abundant hits (Bayesian false discovery rate (BFDR) ≤ 0.02, fold change (FC) ≥ 3) for the indicated bait. Two protein–protein interaction networks are shown: androgen receptor binding (for WT) and structural constituent of the nuclear pore (for 22YtoS). The line thickness corresponds to the strength of published data supporting the interaction, generated from STRING (string-db.org). Additional GO results are provided in Extended Data Fig. 3h and Supplementary Data Table 1. **f**, Representative results of PLAs in DHT-treated PC3 cells using the indicated antibodies are shown in cyan, with DAPI staining in magenta (*n* > 3). Streptavidin (strep.) labeling is shown in green, with DAPI in blue (far right) in DHT-treated PC3 cells. The boxes correspond to magnified regions of the images, that illustrate the differences in interactions between WT AR and 22YtoS. Scale bars, 10 μm. **g**, Transcriptional activity (average ± s.e.m.) of AR and Tyr to Ser mutants, assessed using a luciferase reporter assay for AR (*t*_DHT_ = 1 h, top) or AR-V7 (bottom) in HEK293 cells. Empty stands for empty vector, and *P* were calculated using a Dunnett’s multiple-comparison test against the WT (*n* = 3, top; *n* = 4, bottom). Source data

**Extended Data Fig. 3:**
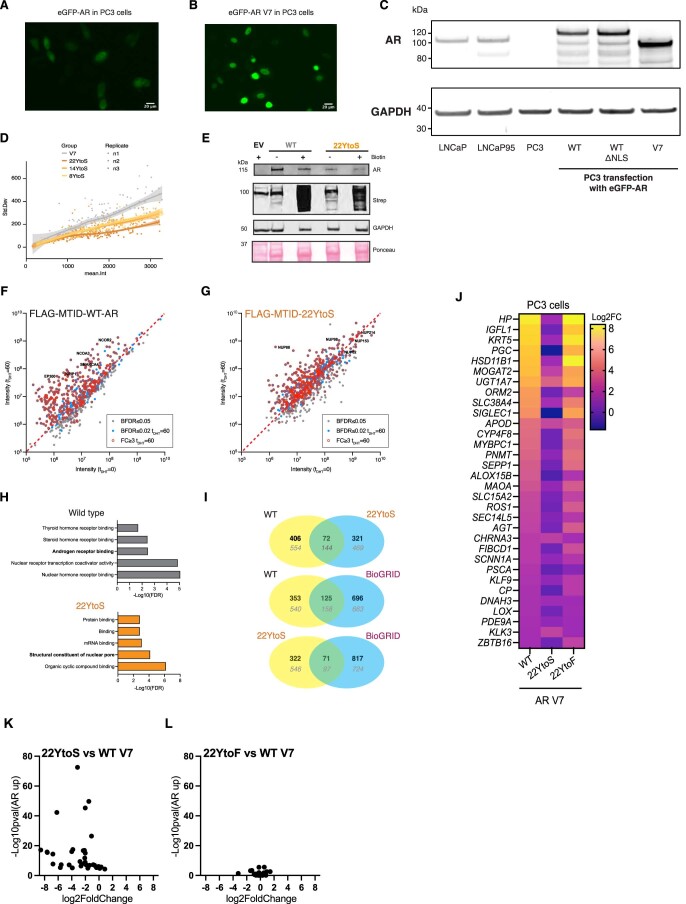
Tyrosine to serine mutations decrease AR granularity, translocation and alter its interactome. **a, b**) Representative (*n* > 3) images of PC3 cells expressing eGFP-AR (A) and eGFP-AR V7 (B). **c**) Expression levels of transfected PC3 cells with eGFP-AR constructs and endogenous levels of AR in LNCaP and LNCaP95 cells. GAPDH was used as loading control. **d**) Quantification of the granularity (s.d.) as a function of the mean nuclear intensity. **e**) Western blot of FLAG-MTID-AR or FLAG-MTID-Y22toS proteins in PC3 cells. **f, g**) Scatter plot of the protein intensities at t_DHT_ = 0 and 60 min for PC3 cells expressing FLAG-MTID-WT-AR (F) and FLAG-MTID-22YtoS (G) following SAINTq analysis. Proteins with a BFDR ≤ 0.05 are shown (gray circle) and those with a BFDR ≤ 0.02 and/or FC ≥ 3in t_DHT_ = 60 min are highlighted in blue and red. **h**) Enriched gene ontology molecular function (GO-MF) categories in FLAG-MTID-WT-AR and FLAG-MTID-22YtoS samples (t_DHT_ = 60 min). The 75 most abundant proteins, with a cutoff of BFDR ≤ 0.02 and FC ≥ 3, were analyzed using STRING and GO categories. The -log_10_(FDR) for selected categories are shown: those highlighted in Fig. 2e are in bold (Supplementary Data Table 1. **i**) Venn diagrams showing proteins identified in WT and 22YtoS (top), WT and AR interactions reported in BioGRID and Y22toS and AR interactions reported in BioGRID. Number of proteins identified (t_DHT_ = 0 and 60 min) with a BFDR ≤ 0.02 and a FC ≥ 3 in bold and numbers of proteins identified with a BFDR ≤ 0.05 in gray (Supplementary Data Table 1). **j**) Gene expression in AR-V7 mutants: heatmap of log_2_ fold changes (log_2_FC) compared to the empty vector transduced control PC3 line (Supplementary Data Table 5). Selected genes shown are a composite of several GSEA ‘AR up’ genesets (Broad Institute) upregulated by AR V7 in PC3 cells. K, L) Scatter plots of the selected genes comparing 22YtoS (**k**) or 22YtoF (**l**) to WT AR V7 according to a one-tailed *t*-test comparing calculated NES to the permuted null distribution, with *P*value adjustment using Benjamini-Hochberg procedure. Source data

To test the functional relevance of phase separation, we transiently expressed enhanced green fluorescent protein (eGFP)-tagged wild-type full-length AR and mutants containing the 8YtoS, 14YtoS or 22YtoS substitutions in the AD in AR-negative PC3 cells. The expression levels of wild-type and mutant full-length AR were heterogeneous and, in certain cell populations, higher than those of endogenous AR in relevant cell lines (Extended Data Fig. 3a–c). For both wild-type and mutant AR, only cells exhibiting low fluorescence emission were therefore considered for analysis. In contrast to wild-type AR, none of the YtoS mutants formed condensates upon dihydrotestosterone (DHT) treatment (Fig. 2a). In addition, although these substitutions do not alter the native nuclear localization signal (NLS)^34^ (^629^RKLKK^633^), they decreased the nuclear translocation rate of the AR: when the amount of time that had elapsed since DHT treatment (*t*_DHT_) was 60 min, WT AR was localized within the nucleus, 8YtoS and 14YtoS were distributed roughly evenly between the cytosol and nucleus and 22YtoS remained in the cytosol (Fig. 2a,b). Next, we transfected cells with wild-type and mutant eGFP-AR-V7 variants, which are localized in the nucleus, and measured cluster formation (Fig. 2c). We observed a decrease of the spatial variance of fluorescence intensity, that is granularity, in cells expressing the 8YtoS, 14YtoS or 22YtoS mutants, indicating that the propensity of these cells to form clusters was reduced (Fig. 2c,d and Extended Data Fig. 3d).

To probe the mechanistic basis of reduced translocation of phase-separation-deficient AR mutants, we mapped the interactomes of WT and 22YtoS full-length AR using proximity-dependent BioID–mass spectrometry (BioID–MS), a technique to systematically identify the interactions of a specific protein in living cells. The WT AR and the 22YtoS mutant were fused to a FLAG-tagged Mini-TurboID (MTID) enzyme and introduced into PC3 cells using a lentiviral vector. The addition of biotin for 1 h led to increased protein labeling, demonstrating that the MTID enzyme was functional (Extended Data Fig. 3e). We collected samples for BioID–MS before and after 60 minutes of DHT treatment (*t*_DHT_, 60 min) and a significance analysis of interactome quantification (SAINTq), a sampling method to assign confidence scores to protein-protein interactions, revealed that many proteins were enriched after DHT stimulation in cells expressing WT AR (Extended Data Fig. 3f,g). Enrichment analysis (STRING) identified enriched proteins in categories related to transcription, including a number of proteins that have been established to interact with the AR (Fig. 2e, Extended Data Fig. 3h and Supplementary Data Table 1). By contrast, fewer proteins were identified in 22YtoS cells, with little overlap with the WT AR proteins (Extended Data Fig. 3h,i): enrichment analysis of 22YtoS cells uncovered enriched proteins in several categories related to nuclear transport. Five nucleoporins were identified among the top 75 most enriched proteins (Fig. 2e, Extended Data Fig. 3h and Supplementary Data Table 1). To validate these observations, we performed proximity ligation assays (PLAs) for several of the top hits, including the SWI–SNF component ARID1A, the Mediator component MED1 and NUP153, in both WT and AR-mutant cells. There was an evident PLA signal for WT AR in association with MED1 and with ARID1A, whereas no such interaction was observed for the 22YtoS mutant (Fig. 2f); by contrast, the 22YtoS mutant, but not WT AR, exhibited an interaction with NUP153 in the perinuclear space (Fig. 2f).

We measured the transcriptional activity of both AR and AR-V7 in cells that had been transiently co-transfected with a luciferase reporter gene driven by an AR-dependent promoter. We found that substitutions of tyrosines in the AR AD led to a reduction of the transcriptional activity of both full-length AR and the AR-V7 splice variant (Fig. 2g).

Finally, we investigated the dependency of gene expression by AR-V7 on the aromatic character of the AR AD. As expected, for the 22YtoS mutant, gene expression was greatly reduced relative to that of WT AR-V7 and substituting all tyrosines with phenylalanines, another aromatic residue, restored gene expression levels to those obtained for WT AR-V7 (Extended Data Fig. 3j–l). Taken together, these results indicate that AR aromatic mutants with reduced phase separation have a lower nuclear translocation rate, increased association with the nuclear pore and reduced gene expression and thus transcriptional activity.

### Short transient helices enhance AR phase separation

**Fig. 3:**
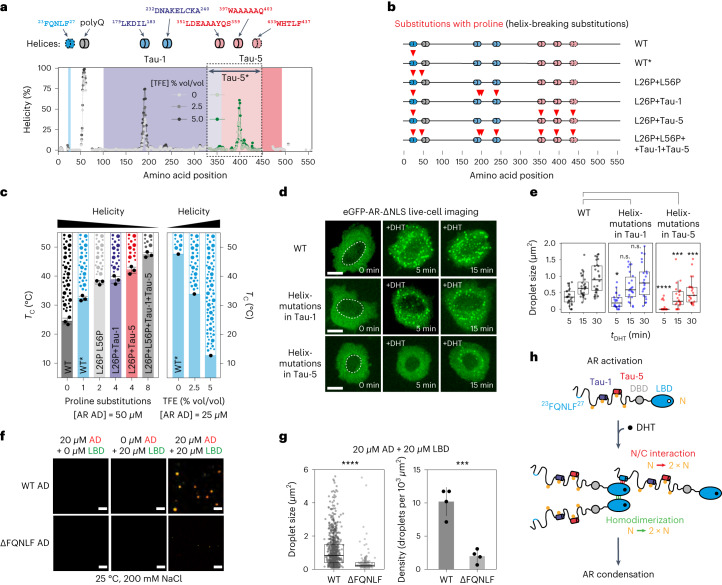
Short transient helices enhance AR phase separation. **a**, Annotation of short helical motifs in the AR AD. The plots show the helical propensity of the WT* AD, measured by NMR in the absence or presence of 2.5% or 5% TFE. Tau-1 and Tau-5 are highlighted. A discontinuous contour indicates motifs that fold when bound to globular binding partners. Helicity values were derived from the main-chain chemical shifts by using δ2D (ref. ^67^). Green values are from an equivalent experiment carried out with the Tau-5* construct (ref. ^23^), which was done because the most informative resonances are invisible in AR AD owing to their involvement in transient long-range interactions. **b**, The mutants that were used to investigate the effect of reduced helical propensity on phase separation. The color code is the same as that in **a**. **c**, *T*_c_ measurements of purified AR AD proteins containing proline substitutions (mean ± s.d., *n* = 3 independent samples), or in the presence of TFE. The solid shading represents the one-phase regime, and droplets represent the two-phase regime. **d**, Representative (*n* > 3) live-cell fluorescence microscopy images of DHT-treated PC3 cells expressing the indicated eGFP-AR-ΔNLS mutants. Scale bar, 10 μm. **e**, Distributions of droplet size for eGFP-AR-ΔNLS and mutants in PC3 cells as a function of *t*_DHT_. Each dot corresponds to the mean droplet size in a single cell (*n* > 20 cells), boxes shown the mean and the quartiles of all cells and *P* values were calculated using a Mann–Whitney *U* test. n.s., not significant. **f**, Representative (*n* > 3**)** fluorescence microscopy images of purified AR AD (WT and ΔFQNLF), the LBD and an equimolar mixture of the two proteins in vitro. In the images, the red (AR AD) and green (LBD) channels are merged; 200 mM NaCl and 20 μM protein were used. Approximately 1% of the total amount of protein is labeled. Scale bars, 5 μm. **g**, Distributions of the size of droplets (*n* = 750 droplets for WT and *n* = 150 for ΔFQNLF) from the samples in **f,** where boxes show the mean and quartiles of all droplets, and average density of droplets in the cells (*n* = 4 independent samples). **h**, Scheme illustrating how the N/C interaction and LBD homodimerization each double the valency (*N*) of the freely diffusing AR species, thus increasing AR phase separation propensity. Source data

Transcriptional activation involves interactions between AD motifs—also known as activation units—and members of the transcriptional machinery^35,36^. Some motifs are known to fold into ɑ-helices when interacting^37,38^. Therefore, we tested whether such motifs in the AR AD contribute to its phase separation behavior. We identified seven regions with helical propensity in the AR AD using NMR. These included the flanking region of the polyglutamine (pQ) tract starting at position 58 and the ^179^LKDIL^183^ motif in the Tau-1 region (Fig. 3a)^39,40^. To map the Tau-5 region, which has low peak intensity in the spectrum of full-length AR AD, we performed NMR on a Tau-5 fragment (Tau-5*), which confirmed the high helical propensity of the ^397^WAAAAAQ^403^ motif (Fig. 3a)^41^. Previous work has shown that the ^23^FQNLF^27^motif forms an ɑ-helix when interacting with the AR LBD^29^ in an interaction known as the N/C interaction. Our previous NMR experiments have shown that the ^433^WHTLF^437^ motif in Tau-5 forms a helix when interacting with TFIIF and have identified two additional motifs, ^232^DNAKELCKA^240^ and ^351^LDEAAAYQS^359^, with weak helical propensity^23,42^ (Fig. 3a) that reached approximately 5% in the presence of 5% trifluoroethanol (TFE), a co-solvent that stabilizes the transient helices formed by disordered peptides and proteins^43^.

To investigate the contribution of helical propensity to AR phase separation, we introduced helix-breaking proline substitutions in the AR AD within or immediately adjacent to the helices (Fig. 3b) and measured the *T*_c_ of AR AD proteins (Fig. 3c). We found that L26P (WT*) increased the *T*_c_ by 8 °C (Fig. 3c). Next, we studied three mutants, all in the L26P background, designed to decrease helicity of the polyQ tract (L56P), Tau-1 (A186P, L192P and C238P) or Tau-5 (A356P, A398P and T435P). We observed that these alterations increased the *T*_c_ to varying degrees: L56P increased it by 5 °C, as did substitutions in Tau-1, but those in Tau-5 had a larger effect, of approximately 10 °C. (Fig. 3b,c). We also analyzed the effect of TFE on phase separation propensity: it increased the helical propensity of the most helical motifs in Tau-1 and Tau-5 (Fig. 3a) and decreased the *T*_c_ of the AD, by 12 °C at 2.5% TFE (vol/vol) and by 35 °C at 5% TFE (Fig. 3c), suggesting that regions with helical propensity enhance AR AD phase separation in vitro^44^.

**Extended Data Fig. 4:**
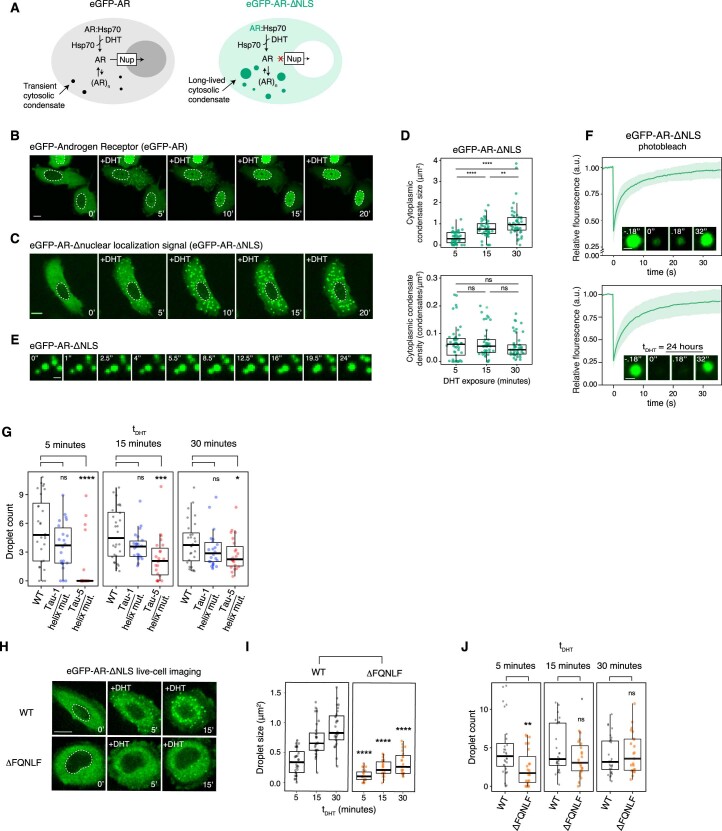
Transactivating units and motifs with helical propensity in AR AD contribute to condensation of AR in vitro and in cells. **a**) Schematic model describing the nuclear translocation pathway of eGFP-AR and cytoplasmic retention of eGFP-AR-ΔNLS upon exposure to ligand (DHT). **b, c**) Time-lapse fluorescence microscopy of eGFP-AR (A) and eGFP-AR-ΔNLS (B) condensates upon treatment with 1 nM dihydrotestosterone (DHT) in transiently transfected PC3 cells. Scale bar: 10 µm. Dashed line indicates the nuclear periphery. **d**) Distributions of average condensate size and density. Each dot corresponds to the mean values measured in an individual cell (*n* = 45 cells). *P*values are from Mann-Whitney U tests. n.s.: not significant. **e**) Snapshots at the indicated time points highlighting a fusion event of eGFP-AR-ΔNLS condensates in the cytoplasm of a PC3 cell. Scale bar: 1 µm. **f**) Fluorescence recovery after photobleaching (FRAP) analysis of cytoplasmic eGFP-AR-ΔNLS condensates in PC3 cells 1 hour and 24 h after addition of 1 nM DHT (t_DHT_ ≈ 1 h). Average relative fluorescence intensity curve of the eGFP-AR-ΔNLS cytoplasmic condensates as a function of time is shown. Error bars represent s.d. of *n* = 34 condensates per time point. Within the box, representative images of condensates before and after photobleaching are shown. Scale bar: 1 µm. **g**) Effect of the mutations introduced in Tau-1 and Tau-5 on the density of the cytosolic condensates formed by eGFP-AR-ΔNLS as a function of t_DHT_ in PC3 cells. Each dot corresponds to a cell (*n* > 20 cells). *P*values are from a Mann-Whitney U test. **h**) Effect of deleting the region of sequence of the AD containing the ^23^FQNLQ^27^ motif on the cytosolic condensates formed by eGFP-AR-ΔNLS upon addition of DHT. Scale bar: 10 μm. The dashed line indicates nuclear periphery. **i, j**) Effect of deleting the region of sequence of the AD containing the ^23^FQNLQ^27^ motif on the distribution of average droplet size (I) and droplet density (J) of the cytosolic condenstates formed by eGFP-AR-ΔNLS as a function of t_DHT_, where each dot corresponds to a cell (*n* > 20 cells). *P*values are from Mann-Whitney U tests. Source data

We examined the effect of reduced AR AD helicity on phase separation in cells. For this purpose, we developed an assay to stabilize the cytosolic AR condensates by deleting the native NLS (^629^RKLKK^633^) to obtain eGFP-AR-ΔNLS (Extended Data Fig. 4a). DHT stimulation of PC3 prostate cancer cells expressing this variant led to the formation of large cytosolic AR condensates, which can facilitate the use of live-cell imaging to examine how substitutions affect the size of the condensates because the lack of interactions with chromatin does not limit their growth (Extended Data Fig. 4b,c). The condensates were spherical; their number hardly changed over time, but their size increased substantially (Extended Data Fig. 4d). In addition, the condensates fused (Extended Data Fig. 4e) and recovered fluorescence intensity quickly after photobleaching (mobile fraction = 94 ± 8%, and half-time of recovery (*t*_1/2_) = 2.29 ± 1.17 s) both 1 h and 24 h after DHT stimulation (Extended Data Fig. 4f). Helix-breaking substitutions in Tau-1 had a negligible effect on the formation and dynamics of cytosolic AR condensates, but substitutions in Tau-5 decreased the number and size of condensates following short-term (5–15 min) hormone exposure (Fig. 3b,d,e and Extended Data Fig. 4g). This indicates that regions with helical propensity in the Tau-5 subdomain enhance AR phase separation in cells^45^.

Our results show that aromatic residues drive AR phase separation but do not explain why hormone binding triggers it (Fig. 2a). Androgen binding to the LBD causes a conformational change that leads to AR oligomerization due to the formation of two dimerization interfaces: the N/C interaction^29^ and the homotypic dimerization of the AR LBD^46^. These dimerization processes do not change the strength of the interactions between aromatic residues, but each doubles the valency of the freely diffusing AR species. According to theory^47^, coarse-grained simulations^48^ and experiments^49^, this decreases the minimal concentration needed for phase separation. In agreement with this hypothesis, eGFP-AR-ΔNLS lacking the ^23^FQNLF^27^ motif (ΔFQNLF) formed fewer and smaller condensates in PC3 cells (Extended Data Fig. 4h–j). Similarly, when we incubated the AD in vitro with 1 molar equivalent of hormone-bound LBD, at a concentration (20 µM) and solution conditions (25 °C, 200 mM NaCl) that do not lead to its phase separation, we observed the formation of droplets containing both domains (Fig. 3f,g); by contrast, AD lacking the ^23^FQNLF^27^ motif formed smaller condensates (Fig. 3f,g). We conclude, therefore, that AR oligomerization upon activation leads to its phase separation (Fig. 3h).

### Rational design of small molecules with enhanced potency

**Fig. 4:**
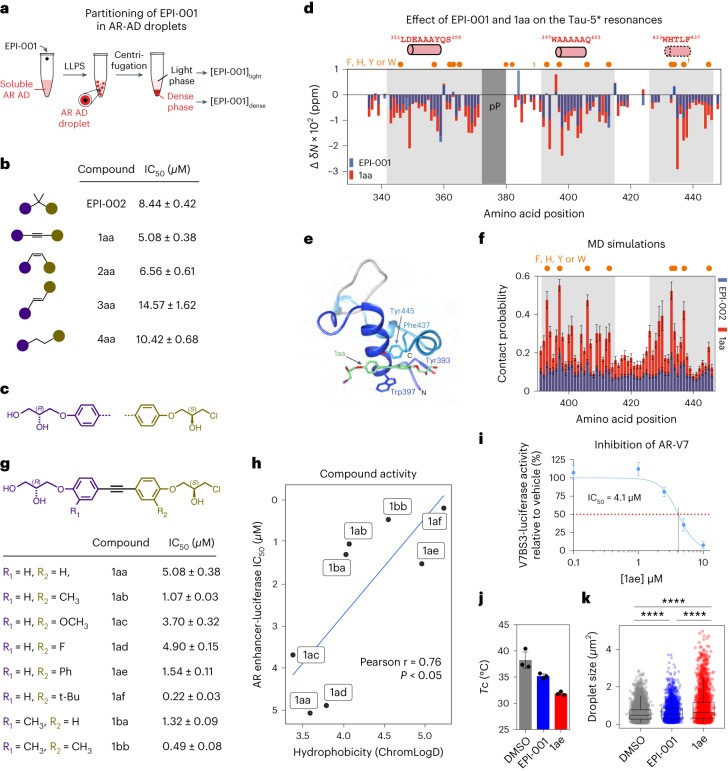
Optimization of the structure of EPI-001. **a**, Experimental set-up for the measurement of the partition coefficient of EPI-001 in condensed AR AD. LLPS, liquid–liquid phase separation. **b**,**c**, Chemical structures of EPI-002 and compounds, with a modified linker between the two aromatic rings of EPI-002. **b**, Schematic of the structures and the corresponding IC_50_ measured in androgen-induced PSA-luciferase assay. Purple and brown circles correspond to chemical groups depicted in c, in which hydrogens are at the R_1_ and R_2_ positions. **d**, Changes in ^15^N chemical shift (δ*N*) in the NMR spectra of Tau-5* (60 μM) as a function of amino acid positions, caused by addition of 1 molar equivalent of EPI-001 (blue) or 1aa (red). Orange circles indicate aromatic amino acids positions in the sequence of Tau-5*. R1-3 (ref. ^23^) and polyP regions are highlighted in light and dark gray, respectively. Samples contained 200 mM NaCl and 2% DMSO-d_6_. **e**, Illustrated molecular dynamics (MD) snapshot of the AR AD interacting with 1aa. Helices are shown in dark and light blue; the loop between them is gray. 1aa is shown in green, and chlorine in purple. **f,** Per-residue contact probabilities observed in REST2 MD simulations between Tau-5 residues 391–446 and the compounds EPI-002 (blue) or 1aa (red). Contacts are defined as occurring in frames in which any non-hydrogen ligand atom is within 6.0 Å of a non-hydrogen protein atom. Orange circles represent the positions of aromatic residues. Values are presented as mean ± statistical errors from block averaging. **g**, Compounds developed from 1aa, and their corresponding potency in the androgen-induced PSA-luciferase assay. **h**, Correlation between the activity of the compounds in the PSA-luciferase assay and their hydrophobicity in terms of LogD determined by chromatography (ChromLogD). **i**, Dose-dependent inhibition of AR-V7 transcriptional activity by 1ae. **j**,**k**, Effect of 1 molar equivalent EPI-001 and 1ae on the *T*_c_ of AR AD (average ± s.d., *n* = 3 independent samples) (**j**) and on the distribution of droplet sizes (*n* > 4,000 droplets for DMSO, *n* > 2,500 for EPI-001 and *n* > 2,000 for 1ae), where boxes show the mean and the quartiles of all droplets (**k**). Source data

EPI-001 is a small-molecule inhibitor of the AR AD, identified by phenotypic screening, that was investigated in clinical trials but was insufficiently potent^24,50^. Solution NMR and molecular simulations have shown that the compound forms a dynamic complex with a collapsed, partially helical state of Tau-5 that is stabilized in part by interactions between the aromatic rings of EPI-001 and the side chains of aromatic residues^23,51^. Because the condensates are stabilized by interactions between aromatic residues (Fig. 1) and the conformation adopted by the AR AD in this complex is related to that leading to condensation (Fig. 3), we hypothesized that EPI-001 partitions within the condensates formed by the AR AD and that this contributes to its mechanism of action. To investigate this, we measured, using high-performance liquid chromatography (HPLC), the equilibrium concentrations of EPI-001 in a biphasic system (Fig. 4a) and found that it indeed partitions within the AR AD liquid phase, with a high partition coefficient (*P*^WT^_EPI-001_ = [EPI-001]_dense_ / [EPI-001]_light_ ≈ 55). We also studied its partitioning in the condensates formed by the 8YtoS mutant and obtained an approximately 40% lower partition coefficient (*P*^8YtoS^_EPI-001_ ≈ 32), indicating that the aromatic character of the AR AD liquid phase is a determinant of partitioning.

**Extended Data Fig. 5:**
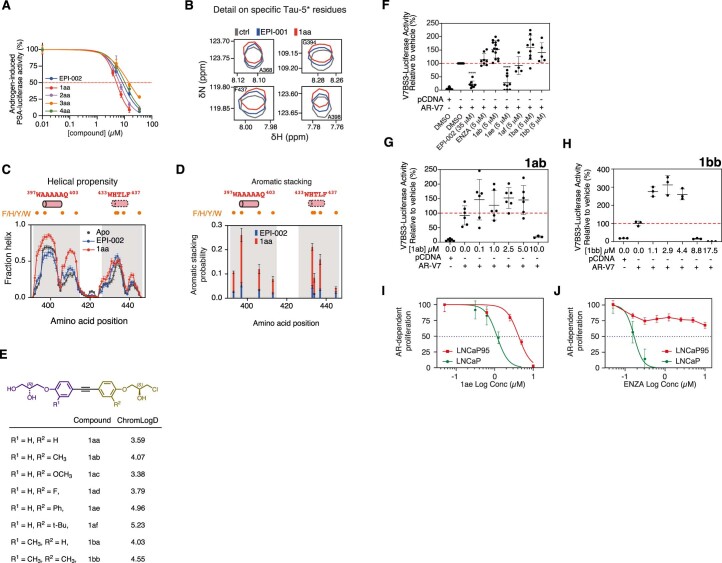
Characterisation of small molecules with enhanced potency. **a**) Inhibition (average ± s.e.m., *n* = 3) of the androgen-induced full-length AR transcriptional activity by compounds shown in Fig. 4a. **b**) Selected regions of Tau-5* ^1^H,^15^ N BEST-TROSY spectra in the absence (gray) and presence of 1 mol equivalent of EPI-001 (blue) and 1aa (red). **c**) Helical propensities of Tau-5R2_R3 in its apo form (black) and in bound conformations obtained from simulations run in the presence of EPI-002 (blue) and 1aa (red) with an indication of the positions of helical motifs and aromatic residues in the sequence. The data was obtained from the 300 K REST2 MD simulations. Values are presented as averages ± statistical errors from block averaging. **d**) Populations of aromatic stacking contacts between aromatic side chains of Tau-5R2_R3 and aromatic rings of EPI-002 (blue) and 1aa (red) with an indication of the positions of helical motifs and aromatic residues. The data was obtained from the 300 K REST2 MD simulations. Values are presented as averages ± statistical errors from block averaging. **e**) ChromLogD values of compounds developed from 1aa scaffold reporting their hydrophobicity (n = 3). **f**) Comparison of EPI-002 (35 µM) and enzalutamide (ENZA, 5 µM) with the most potent compounds (5 µM) to block AR-V7 transcriptional activity (average ± s.e.m., *n* = 3). **g, h**) Lack of a dose-dependent inhibition of AR-V7 transcriptional activity for 1ab (g) and 1bb (g). LNCaP cells that ectopically expressed AR-V7 were co-transfected with a V7BS3-luciferase reporter gene construct and incubated with the indicated concentrations of the compounds (average ±s.e.m., *n* = 3). **i, j**) 1ae blocked the proliferation of both LNCaP cells in response to androgen and AR-V-driven proliferation of LNCaP95 cells whereas Enzalutamide (ENZA) blocked androgen-induced proliferation driven by full-length AR in LNCaP cells but had poor potency against AR-V-driven proliferation of LNCaP95 (LN95) cells (average ± s.e.m., *n* = 3). Source data

We hypothesized that optimizing the distance and orientation of aromatic rings, and modulating the flexibility of the functional group connecting them, would facilitate the interaction of this molecule with the aromatic side chains of the target and increase its potency. We synthesized a series of compounds in which the carbon atom between the aromatic rings of EPI-002, the (2*R*,19*S*) stereoisomer of EPI-001, was replaced by two carbon atoms separated by a single (compound 4aa), a double (2aa, *cis* and 3aa, *trans*) or a triple (1aa) bond (Fig. 4b,c). The potency of the compounds was evaluated in LNCaP cells transfected with a luciferase reporter driven by an AR-dependent promoter and enhancer^24,50,52^. Compounds 2aa and, in particular, 1aa were the most potent inhibitors, and were substantially more potent than EPI-002 (Fig. 4b and Extended Data Fig. 5a).

To confirm that this led to an optimized interaction with the AR AD, we analyzed the NMR spectrum of Tau-5* in the presence of the compounds. The chemical-shift perturbations caused by 1 molar equivalent of 1aa were larger than those induced by EPI-001, indicating that the interaction’s strength was enhanced (Fig. 4d and Extended Data Fig. 5b). We also simulated the interaction of the compounds with residues 391 to 446 of the AR AD^51^ (Fig. 4e) and observed that its atoms contacted those of 1aa more frequently than those of EPI-002, leading to a more stable and structured complex. The simulated dissociation constant (*K*_D_) was 1.4 ± 0.1 mM for 1aa versus 5.2 ± 0.4 mM for EPI-002, in agreement with the NMR and gene reporter data (Fig. 4f and Extended Data Fig. 5c,d). Given that the droplets formed by the AR AD are stabilized by hydrophobic and aromatic interactions (Fig. 1), we synthesized analogs of 1aa with substitutions in positions R_1_ and R_2_ (Fig. 4g,h and Extended Data Fig. 5e) to modulate the hydrophobic and aromatic character of the compounds. Introduction of a methyl (CH_3_) group at R_1_ (achieved with compound 1ba) or R_2_ (compound 1ab) increased the potency, reflected by the half-maximal inhibitory concentration (IC_50_) changing from approximately 5 µM to approximately 1 µM; after introduction of this group in both positions (compound 1bb), the IC_50_ was 0.5 µM in the luciferase reporter system. In line with this, the introduction of a tert-butyl (C(CH_3_)_3_) group at R_2_, bearing three methyl groups (compound 1af), led to an IC_50_ of approximately 0.22 µM. Substitution of hydrogen by fluorine (compound 1ad) or a methoxy (CH_3_O) group (compound 1ac) at position R_2_ barely changed the IC_50_, but introduction of an additional aromatic ring (compound 1ae) at this position led to an IC_50_ of approximately 1.5 µM (Fig. 4g,h).

Next, we measured the inhibitory potential of the compounds using the V7BS3-luciferase reporter, designed specifically for AR-V7 (ref. ^53^). As expected, 5 µM enzalutamide, which binds to the AR LBD, had no activity against AR-V7-induced V7BS3-luciferase activity, whereas 35 µM EPI-002 blocked luciferase activity, consistent with previous reports^52^ (Extended Data Fig. 5f). Notably, 1ae was the most potent inhibitor of AR-V7 transcriptional activity, in a dose-dependent manner (Fig. 4i) (IC_50_ = 4.1 µM), whereas 1ab and 1bb had no inhibitory effects (Extended Data Fig. 5g,h). In line with these results, 1ae blocked the proliferation of both LNCaP and LNCaP95 cells, driven by full-length AR and AR-V7, respectively (Extended Data Fig. 5i), whereas enzalutamide blocked the proliferation of only LNCaP cells, consistent with its mechanism of action (Extended Data Fig. 5j). In addition, to confirm that partitioning in the AR AD condensates contributes to the mechanism of this optimized AR AD inhibitor, we compared the extent to which EPI-001 and 1ae decrease the T_c_ of the AR AD and increase the size of the droplets that it forms. In agreement with polyphasic linkage^54,55^, we found that EPI-001 decreases the *T*_c_ and increases the size of the droplets, and that, at the same concentration, 1ae has a larger effect (Fig. 4j,k).

**Fig. 5:**
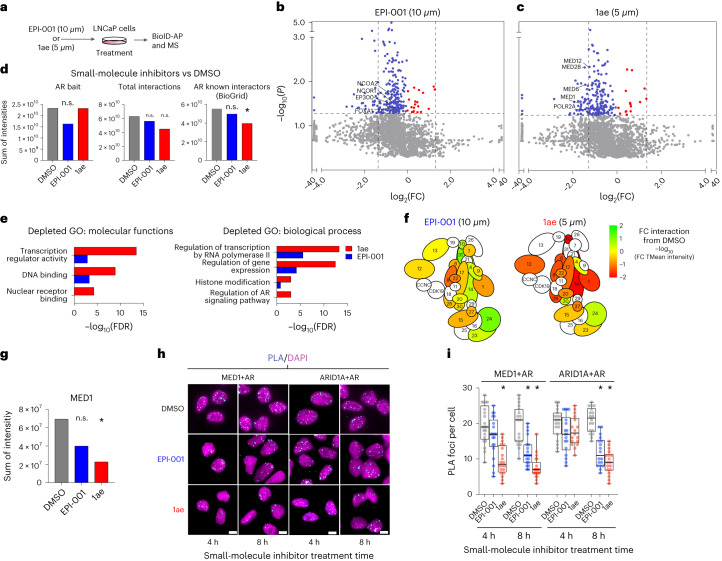
1ae decreases interactions between AR and the transcription machinery. **a**, Schematic of the method for small-molecule treatment of cells and the BioID experiment. **b,** BioID–MS of LNCaP MTID-AR-WT cells treated with EPI-001 (10 μM, 1 h), followed by treatment with DHT and biotin (2 h). Intensity data were obtained from SAINTq analysis. Shown is the log_2_(FC) of the intensity of the interaction in inhibitor-treated versus DMSO-treated cells. Decreased interactors in inhibitor-treated cells relative to DMSO-treated cells are shown in blue; *P* < 0.05. The dashed lines mark where log_2_(FC) < −1.5. Increased interactors in inhibitor-treated cells relative to DMSO-treated cells are shown in red; *P* < 0.05. The dashed lines mark where log_2_(FC) > 1.5. Proteins of interest are annotated (*n* = 3). **c**, As in **b**, BioID–MS of LNCaP MTID-AR-WT cells. Cells were treated for 1 h with 1ae (5 μM) and then for 2 h with DHT and biotin (*n* = 3). **d**, (Total mean) TMean intensities of peptides identified by MS (*n* = 3) from SAINTq data of individual proteins (bait), total interactors (all) or collated known AR interactors sourced from BioGrid (https://thebiogrid.org/) were compared between LNCaP MTID-AR-WT cells treated with DMSO and those treated with small-molecule inhibitors. **e**, GO search terms of key biological processes and molecular functions in SAINTq intensity data from **b** and **c** (BFDR < 0.02, depleted = log_2_(FC) < −1.5), obtained from the LNCaP MTID-AR-WT cells treated with small-molecule inhibitors versus DMSO. Full categories are available in Supplementary Data Table 2. **f**, BioID–MS of the LNCaP MTID-AR-WT interaction with Mediator complex in cells treated with small-molecule inhibitors versus DMSO. SAINTq data, color indicates strength of interaction change from logFC_10_ TMean of intensity. **g**, The TMean intensity of interactions with the MED1 subunit of Mediator was compared between LNCaP MTID-AR-WT cells treated with DMSO or small-molecule inhibitors. SAINTq data were used. Statistical significance was determined by Student’s *t*-test against the control group. **h**, Results from a PLA in LNCaP MTID-AR-WT cells, using the indicated antibodies shown in cyan with DAPI staining shown in magenta. Cells were treated with small-molecule inhibitors or DMSO and DHT at the indicated times. Scale bars, 10 μm**. i**, Distributions of PLA foci per cell, each dot corresponds to a cell and boxes shown the mean and quartiles of all cells (*n* > 20 cells). Source data

**Extended Data Fig. 6:**
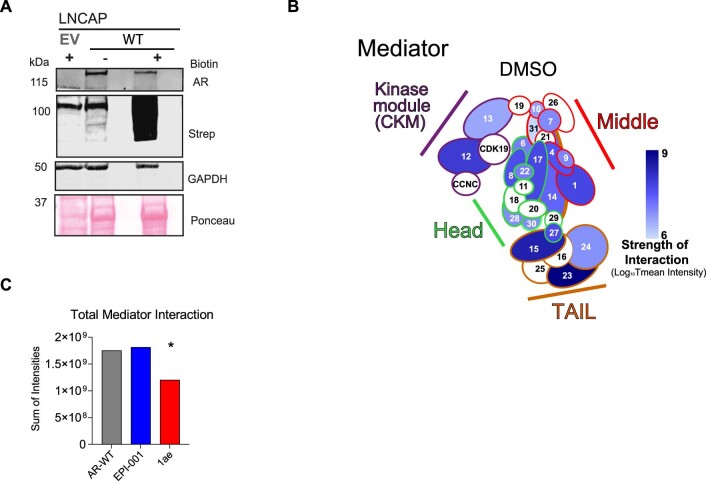
Small molecule inhibitors alter AR proteomic interactions with Mediator. **a**) Representative (*n* = 2) western blot showing expression of FLAG-MTID-AR or FLAG-MTID-Y22toS proteins in LNCaP cells with antibodies for AR. Biotin-dependent labeling is shown with Streptavidin antibodies (Strep) and GAPDH and Ponceau staining are shown as loading controls. EV indicates the empty vector expressing FLAG-MTID. **b**) BioID MS of LNCaP MTID-AR-WT interaction with Mediator complex. Colour indicates strength of interaction from FLAG, LogFC10 Tmean of intensity. **c**) TMean SAINTq intensity of total mediator interactions were compared across LNCaP MTID-AR-WT with DMSO or treated cells with small molecule inhibitors. Source data

To understand the mechanisms by which the inhibitors decrease AR transcriptional activity, we performed a BioID–MS analysis in LNCaP cells stably expressing MTID-AR-WT that were treated with EPI-001 or 1ae (Fig. 5a and Extended Data Fig. 6a) and found that both inhibitors caused a general decrease in AR interactions (Fig. 5b,c). 1ae caused a stronger decrease in interactions than EPI-001; in addition, it exhibited a significant decrease in interactions with known AR interactors in the BioGrid database (Fig. 5d). An enrichment analysis of the depleted interactors revealed that the decrease was more marked for 1ae in all categories, in line with its higher potency (Fig. 5e and Supplementary Data Table 2). Focusing on Mediator, we found that, among all its subunits, MED1 was the most significantly reduced by 1ae (Fig. 5f,g and Extended Data Fig. 6b,c). Finally, we used PLAs between AR and MED1 or ARID1A to validate these findings and found that, for both inhibitors, the total number of foci per cell was reduced as time of incubation increased (Fig. 5h,i). These data show that the small-molecule inhibitors decrease the extent to which AR interacts with the transcription machinery, thus inhibiting its transcriptional activity.

### 1ae inhibits AR-dependent transcription and tumor growth

**Fig. 6:**
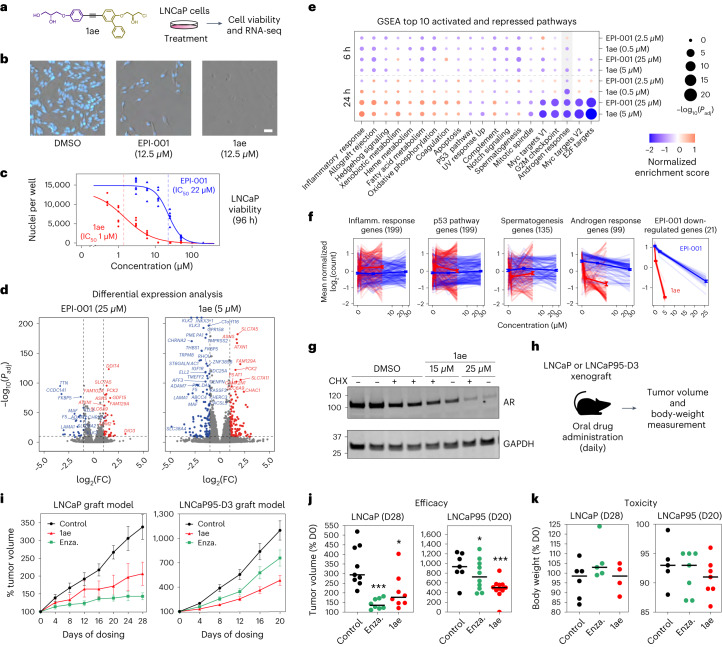
Compound 1ae inhibits AR-dependent transcription and tumor growth. **a**, Structure of 1ae, and a schematic of the experiment used to investigate its effect on LNCaP cells. **b**, Representative (*n* > 3) images of LNCaP cells (stained with Hoechst) after 96 h of treatment. Scale bar, 50 μm. **c**, Dose–response curve (log-logistic fit) of viable LNCaP nuclei, with IC_50_ values calculated from the dose–response curve (*n* = 6). **d**, Volcano plots of differentially expressed genes in LNCaP cells treated with EPI-001 or 1ae for 24 h at a concentration near the IC_50_ versus cells treated with DMSO (fold change cutoffs: 2×, 0.5×). (Supplementary Data Table 3). **e**, Gene set enrichment analysis of the top 10 enriched and top 10 depleted msigdb hallmark signature pathways^68^ in LNCaP cells treated with EPI-001 or 1ae versus those treated with DMSO. Circle size represents the significance of the normalized enrichment score (log(*P*_adj_)), and the color gradient represents normalized enrichment score of the indicated pathway analyzed with GSEA. The hallmark androgen-response pathway is highlighted in gray (*n* = 3). **f**, The log transformation of mean normalized counts of the indicated gene sets in LNCaP cells treated with EPI-001 or 1ae. Light lines represent individual genes, dark lines represent average of all genes and the shaded areas represent the standard error (*n* = 3). **g**, Representative (*n* = 3) western blot of endogenous AR in LNCaP cells pretreated with cycloheximide (CHX) for 3 h, which were then treated with 1ae for 21 h. GAPDH was used as the loading control (bottom). **h**, Schematic of the LNCaP and LNCaP95-D3 xenografting procedure in the CRPC model. **i**, Tumor volume in mice with LNCaP (left) or LNCaP95-D3 (right) xenografts. Values are presented as the mean percentage relative to the volume measured at the first day of treatment with the error bars representing the s.e.m. of *n* ≥ 8 (LNCaP) or *n* ≥ 7 (LNCaP95-D3) tumors per treatment group. Enza., enzalutamide. **j**, Tumor volume on day 28 or 20 of the experiments, presented as the percentage relative to the volume measured at the first day of treatment. **k**, Body weight of animals on day 28 or 20 of the xenograft experiments, presented as percentages relative to the body weight measurement on the first day of treatment. Horizontal bars in **j** and **k** represent the median. Source data

**Extended Data Fig. 7:**
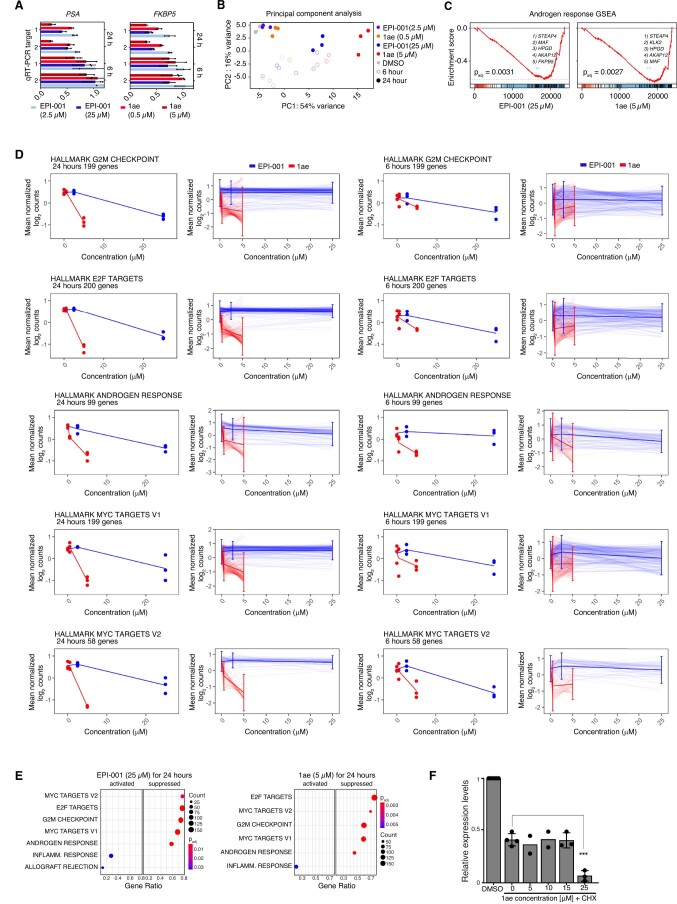
1ae inhibits AR dependent oncogenic pathways in models of CRPC. **a**) qRT-PCR of PSA and FKBP5 transcript targets using two primer pairs for each locus. Values indicate 2^-∆∆Ct^ (Log fold change in target signal versus β-Glucuronidase housekeeping gene signal normalized to values from corresponding DMSO control sample, average ± s.e.m., *n* = 3). **b**) Principal component analysis of LNCaP cells treated with EPI-001 or 1ae (*n* = 3). **c**) Sequential walk of the GSEA running enrichment score of hallmark androgen response pathway genes in LNCaP cells treated with EPI-001 or 1ae, versus DMSO, for 24 h. Top 5 downregulated genes for EPI-001 and 1ae treatment contributing to the leading edge indicated in top right, and adjusted *P*value of GSEA statistic indicated in bottom left (*n* = 3) according to a one tailed *t*-test comparing calculated NES to the permuted null distribution, with *P*value adjustment using Benjamini-Hochberg procedure. **d**) Line plots of mean normalized, log transformed read counts of significantly depleted gene sets in LNCaP cells treated with EPI-001 or 1ae versus DMSO at 24 h (shown in Fig. 6e), as a function of concentration. Light lines represent individual genes, dark lines represent average of all genes, and bars represent s.d. (*n* = 3). **e**) GSEA analysis of RNA-seq experiment showing most significantly activated and suppressed pathways for EPI-001 and 1ae treatment, vs DMSO, at 24 h, ranked by the adjusted *P*value (padj). Gene pathways split by ‘activated’ or ‘suppressed’ based on GSEA enrichment in the gene list ranked by log_2_FC vs DMSO, in order of gene ratio (detected genes/all genes in pathway) of the analyzed pathway. Circles scale to the count of detected genes from the pathway, and color scales to padj from the pathway (*n* = 3), according to a one tailed *t*-test comparing calculated NES to the permuted null distribution, with *P*value adjustment using Benjamini-Hochberg procedure. **f**) Quantification of AR signal, versus DMSO control, normalized to GAPDH signal from western blots of LNCaP cells treated with CHX for 3 h, then 1ae at indicated concentrations for 21 h (*n* = 3, except 5 and 10 μM where *n* = 2). Source data

To further explore the potency and specificity of 1ae and EPI-001 on the AR-driven transcriptional program, we used RNA sequencing (RNA-seq) on LNCaP prostate adenocarcinoma cells after treatment with approximately IC_10_ and IC_50_ doses of the compounds for 6 or 24 h (Fig. 6a–c and Extended Data Fig. 7a,b). Six-hour treatment with IC_10_ concentrations had negligible effects on the gene expression profile of prostate cancer cells (Extended Data Fig. 7a,b); by contrast, 24-h treatment with 25 µM EPI-001 led to the differential expression of 64 genes, and 24-h treatment with 5 µM 1ae led to the differential expression of 231 genes, compared with DMSO-treated control cells (Fig. 6d). Gene set enrichment analysis (GSEA) revealed that downregulated genes were significantly enriched for known AR targets, for both EPI-001 and 1ae (*P*_adj_ < 0.01) (Extended Data Fig. 7c–e). Both EPI-001 and 1ae dysregulated the same subset (5/50) of pathways tested with GSEA (Fig. 6e,f and Extended Data Fig. 7e). The significantly dysregulated pathways included the AR response pathway and other pathways that are known to be active in CRPC^56,57^. Of note, treatment with 5 µM 1ae, a concentration that does not alter AR levels (Fig. 6g and Extended Data Fig. 7f), led to a more profound reduction in the expression of all downregulated and differentially expressed genes that were induced than did treatment with 25 µM of EPI-001 (Fig. 6f and Extended Data Fig. 7d). These results indicate that 1ae inhibits AR-dependent targets in prostate cancer cells and is more potent in its transcriptional inhibitory effect than EPI-001.

**Extended Data Fig. 8:**
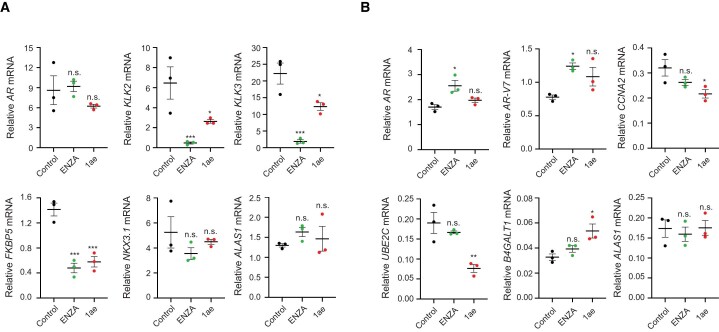
1ae has on-target activity in LNCaP and LNCaP95 xenografts. Real-time PCR of AR, KLK2, KLK3, FKBP5, NKX3.1, and ALAS1 transcript normalized to SDHA harvested from LNCaP tumors (**a**), and AR, AR-V7, CCNA2, UBE2C, B4GALT1 and ALAS1 transcript normalized to SDHA harvested from LNCaP95-D3 tumors (**b**). Both enzalutamide and 1ae show on target activity and do not affect expression of housekeeping gene ALAS1 in LNCaP xenografts. Conversely only 1ae is capable of repressing AR-V7 induced genes, or de-repressing AR-V7 repressed gene B4GALT1. Error bars represent s.e.m. of *n* = 3 samples per treatment arm. Source data

The in vivo efficacy of 1ae was tested on human CRPC xenografts in castrated mice. For this purpose, LNCaP cells (driven by the full-length AR), and LNCaP95-D3 cells (expressing elevated levels of the AR-V7 splice variant)^58^ were xenografted into the mice. 1ae was administered at a daily dose of 30 mg per kg body weight for 28 d (Fig. 6h). After 20–28 d of treatment, 1ae modestly but significantly reduced tumor volumes in both the LNCaP and LNCaP95-D3 xenograft models compared with control animals (Fig. 6i,j): in the AR-V7-driven LNCaP95-D3 xenograft model of CRPC, 1ae outperformed enzalutamide, a second-generation antiandrogen that targets the AR LBD. No overt toxicity was observed for 1ae, as determined by the fact that there were no substantial differences in body weights among the animals at the end of the experiment (Fig. 6k). Finally, we confirmed the in vivo on-target activity of 1ae for both AR-FL and AR-V7 in LNCaP and LNCaP95-D3 xenografts by analyzing gene expression in the tumors. Both enzalutamide and 1ae inhibited androgen-induced genes in LNCaP xenografts, but only 1ae was able to block AR-V7-mediated gene transcription in LNCaP95-D3 xenografts and, notably, it de-repressed the *B4GALT* gene, which is repressed by AR-V7 (ref. ^59^). In both models, neither enzalutamide nor 1ae had an effect on the housekeeping gene *ALAS1* (Extended Data Fig. 8). In summary, although the activity of 1ae on the LNCaP95-D3 xenograft is modest, its superiority to enzalutamide indicates that our approach leads to an inhibition of the AR AD.

## Discussion

Our data provide insights into the molecular basis of phase separation encoded in the AR, which may also apply to other transcriptional regulators. The cytosolic and nuclear condensates that AR forms are stabilized by interactions between aromatic residues, similar to condensates formed by various prion-like proteins^28,60^. In the AR AD, these cluster in the ^23^FQNLF^27^ motif and in the C terminus of the domain, which includes a sequence region that is key for transactivation in the absence of androgens^61^ (Tau-5, Fig. 1c). The N/C interaction, stimulated by hormone binding, also contributes to stabilizing the condensates by increasing the valency of AR^29,46,62^. The presence of partially folded helices in the AR AD further facilitates phase separation, especially in regions of sequence that are rich in aromatic residues, likely by projecting forward aromatic side chains.

Our results reveal unexpected links between phase separation and the functions of transcriptional regulators. We found that reducing phase separation of the AR AD inhibited transcriptional activity, consistent with previous studies on a small number of TFs^15,20–22^ as well as nuclear translocation (Fig. 2a,b,e,f). We speculate that aromatic residues in the AR AD, which drive its phase separation, can interact with aromatic residues in FG repeats of nucleoporins, without the mediation of nuclear import receptors and adapter proteins^63^, to facilitate translocation. This idea is supported by the observation that substituting surface residues with aromatic ones in a large globular protein enhances translocation^64^. We conclude that AR activation by androgens leads to the formation of condensates that are stabilized by interactions between aromatic residues that are key for the receptor to perform its cytosolic and nuclear functions.

The molecular features driving AR phase separation suggest how the compounds that inhibit the AR AD function. We found that they partition into AR condensates in vitro, and that their partitioning into AR condensates is driven by interactions with aromatic residues in the AR AD (Fig. 4a). NMR experiments and molecular simulations revealed that the helical regions of sequence within Tau-5 form a transient binding pocket that facilitates the interaction (Fig. 4e,f). Stabilization of the pocket and covalent modification of the cysteine residues found in the AR AD trap it in a conformation that disfavors interactions with effector partners. This explains why the compounds reduce the *T*_c_ in vitro (Fig. 4j,k) and inhibit AR-co-activator interactions and AR-dependent transcription in cells (Figs. 5a–e and 6c–f). It also suggests that enhancing compound partitioning by optimizing hydrophobicity and aromaticity leads to increased potency of the compounds, consistent with the RNA-seq and viability data (Fig. 6a–f). We propose that other intrinsically disordered proteins contain sequences that assume transient secondary structures in condensates, and that such structures provide transient ‘druggability’ to the target protein, a proposal that is consistent with the evidence for the structure–activity relationship of the AR AD-targeting compounds described here (Fig. 4).

Finally, we show that targeting small molecules to the condensates formed by AR and, specifically, to the region of sequence that drives its phase separation has an antitumorigenic effect, specific to AR-dependent tumor growth, in an in vivo CRPC model driven by an ‘undruggable’ AR variant. Anti-androgens used as first-line therapy against prostate cancer, such as enzalutamide, target the LBD and inhibit activation by androgens^65^. A hallmark of CRPC is the emergence of AR splice variants that lack the LBD and are resistant to this class of drugs. Such isoforms consist of the DNA-binding domain and the disordered AD of the receptor, suggesting that its inhibition could inhibit prostate cell proliferation in CRPC. We took advantage of a previously described small molecule, EPI-001, clarified its mode of action, and improved its potency by using insights into the driving forces of AR phase separation and the physicochemical properties of the condensates that it forms. Our approach, based on the rational optimization of a drug-like small molecule initially identified by phenotypic screening^24,50^, is complementary to alternative strategies based on the identification of inhibitors of AR condensation^19^ and on targeting Cys residues of the AR AD by the addition of electrophilic warheads to existing ligands^66^. In summary we establish a basis on which anti-CRPC drugs can be further developed, and we propose a generalizable framework for targeting with therapeutic intent the phase-separation capacity of intrinsically disordered regions in oncogenic transcription factors^6^.

## Methods

### Materials availability

All unique reagents generated in this study are available with a materials transfer agreement.

### Experimental model and subject details

#### Cell culture

PC3 (ATCC; CRL-1435) and LNCaP clone FGC (ATCC; CRL-1740) cells were cultured in RPMI-1640 containing 4.5 g L^–1^ glucose (Glutamax, Gibco) supplemented with either 10% (vol/vol) charcoal-stripped serum (CSS, Thermo Fisher Scientific A3382101) or 5% FBS (vol/vol), as specified below, and antibiotics. Induction of transcriptional activation by the AR in experiments using 5% FBS cultured LNCaP cells (Fig. 6a–f and Extended Data Figs. 1a,d and 7a–e) was verified using high-resolution microscopy and quantitative reverse transcription PCR (qRT–PCR). HEK293T cells (ATCC; CRL-3216) and AR-eGFP Hela stable cells^69^ (a gift from the M. Pennuto lab) were maintained in DMEM containing 4.5 g L^–1^ glucose supplemented with 10% (vol/vol) charcoal-stripped FBS and antibiotics. LNCaP95 cells were obtained from S. R. Plymate (University of Washington) and cultured in phenol-red-free RPMI supplemented with 10% (vol/vol) charcoal-stripped FBS (Gibco) and antibiotics. Cells were cultured in a humidified atmosphere containing 5% CO_2_ at 37 °C. Cell cultures tested negative for mycoplasma contamination.

#### Human prostate cancer xenografts

All animal experiments adhere to regulatory and ethical standards and were approved by the University of British Columbia Animal Care Committee (A18-0077). Before any surgery, metaCAM (1 mg per kg body weight, 0.05 ml per 10 g body weight) was administered subcutaneously. Isoflurane was used as an anesthetic. CO_2_ was used to euthanize the animals. Six- to eight-week-old male mice (NOD-scid IL2Rgamma^null^) were maintained at the Animal Care Facility at the British Columbia Cancer Research Centre. Five million LNCaP cells were inoculated subcutaneously in a 1:1 volume of matrigel (Corning Discovery Labware). Tumors were measured daily using digital calipers, and the volume was calculated using the formula for ovoid volume: length × width × height × 0.5236. When xenograft volumes were approximately 100 mm^3^, the mice were castrated, and treatment dosing started one week later. Animals were dosed daily by oral gavage with 30 mg per kg body weight of 1ae, 10 mg per kg body weight enzalutamide, or vehicle (5% DMSO, 1.5% Tween-80, 1% CMC).

### Cloning of constructs

The primers and synthetic genes used in this work are listed in Supplementary Data Table 6.

#### GFP-AR FL, V7, and ∆NLS cloning strategy

For peGFPC1-AR-∆NLS, the NLS sequence (RKLKK, corresponding to amino acids 629–633 of AR) of the eGFP-AR fusion protein^70^ was removed from peGFP-C1-AR (Addgene no. 28235) using the Q5 site-directed mutagenesis kit and primer design tools (New England BioLabs). Any clones found to have expansion or shrinkage of either the polyQ or polyG site in the AR were corrected by replacing the 1510-base-pair (bp) KpnI-KpnI fragment with that of the WT AR sequence from peGFP-C1-AR.

For peGFPC1A-V7, the V7 variant of AR was generated from peGFP-C1-AR using the Q5 site-directed mutagenesis kit and primer design tools (New England Biolabs). Any clones that were found to contain expansion or shrinkage of either the polyQ or polyG site in the AR were corrected by replacing the 1510-bp KpnI-KpnI fragment with that of the WT AR sequence from peGFP-C1-AR.

For monomeric eGFP (mEGFP) constructs, mEGFP was subcloned into vectors containing human AR (Addgene no. 29235) and AR-V7 (Addgene no. 86856) using Gibson assembly to create mEGFP-AR-FL and mEGFP-AR-V7 (referred to as AD+DBD+NLS in Fig. 1b and Extended Data Fig. 2a,b) mammalian expression vectors. AR-V7 contains a 16-amino-acid (aa) constitutively active NLS containing an exon that replaces the LBD exons in AR-FL^71^. The sequence downstream of the AR activation domain in AR-V7, containing the DBD and NLS, was subcloned into an mEGFP plasmid (Addgene no. 18696) using Gibson assembly to create the mEGFP-AR-V7-ΔAD (referred to as DBD+NLS in Fig. 1b and Extended Data Fig. 2a,b) expression vector.

#### AR tyrosine mutagenesis strategy

##### Production of YtoS mutants for mammalian expression

The sequences were optimized for expression in human cells, synthesized and cloned into the pUC57 plasmid (high-copy AmpR) by GenScript Biotech. To enable simple excision from pUC57 and insertion into plasmids derived from peGFPC1-AR, two HindIII sites were included as flanks on the fragments. After digestion using HindIII, the resulting 1,722-bp fragments were excised from TBE agarose gels, purified using the E.Z.N.A. MicroElute Gel Extraction Kit (Omega Biotech) and ligated into HindIII-cut, calf intestinal alkaline phosphatase (CIP)-treated and gel-purified peGFPC1-AR, peGFPC1-AR ∆NLS or peGFPC1A-V7 plasmids to produce the YtoS mutants.

##### Production of YtoS mutants for bacterial expression

pDEST17 plasmids for bacterial recombinant production of AR AD YtoS mutants were synthesized by Thermo Fisher Scientific with open reading frame (ORF) sequences flanked with attB1 and attB2 sequences.

#### AR helix-breaking mutagenesis strategy

##### pDONR221-AR-AD-WT

The DNA sequence corresponding to the 1,558-aa fragment of AR-AD was synthesized and encoded in a pDONR221 vector by Thermo Fisher Scientific (flanked with attB1 and attB2 sequences).

##### pDEST17-AR-AD-WT

pDONR221-AR-AD-WT was subcloned into a pDEST17 vector using the LP clonase reaction (Thermo Fisher Scientific).

##### pDEST17-AR-AD-WT*

L26P was introduced into a WT AR sequence (pDONR221-AR-AD-WT) using a Quickchange protocol with Pfu Turbo polymerase (Agilent), and the resulting plasmid with the L26P substitution (pDONR221-AR-AD-WT*) was subcloned into a pDEST17 vector using the LP clonase reaction (Thermo Fisher Scientific).

##### pDEST17-AR-AD-L56P*

L56P was introduced into the pDONR221-AR-AD-WT* (bearing L26P; described above) using a Quickchange protocol with Pfu Turbo polymerase (Agilent) to generate pDONR221-AR-AD-L56P*. The resulting plasmid with the L26P and L56P substitutions (pDONR221-AR-AD-L56P*) was subcloned into a pDEST17 vector using the LP clonase reaction (Thermo).

##### pDEST17-AR-AD-Tau-1*

The A186P, L192P and C238P substitutions were introduced in a step-wise manner into pDONR221-AR-AD-WT* (bearing L26P; described above) using a Quickchange protocol with Pfu Turbo polymerase (Agilent) to generate pDONR221-AR-AD-Tau-1*. The resulting plasmid with the L26P, A186P, L192P and C238P substitutions (pDONR221-AR-AD-Tau-1*) was subcloned into a pDEST17 vector using the LP clonase reaction (Thermo Fisher Scientific).

##### pDEST17-AR-AD-Tau-5*

The A356P, A398P and T435P substitutions were introduced in a step-wise manner into pDONR221-AR-AD-WT* (bearing L26P; described above) using a Quickchange protocol with Pfu Turbo polymerase (Agilent) to generate pDONR221-AR-AD-Tau-5*. The resulting plasmid with the L26P, A356P, A398P and T435P substitutions (pDONR221-AR-AD-Tau-5*) was subcloned into a pDEST17 vector using the LP clonase reaction (Thermo Fisher Scientific).

##### pDEST17-AR-AD-L56P+Tau-1+Tau-5*

The L56P, A186P, L192P and C238P substitutions were introduced in a step-wise manner into pDONR221-AR-AD-TAU-5* (bearing the L26P, A186P, L192P and C238P substitutions; described above) using a Quickchange protocol with Pfu Turbo polymerase (Agilent) to generate pDONR221-AR-AD-L56P+Tau-1+Tau-5*. The resulting plasmid containing the L26P, L56P, A186P, L192P, C238P, A356P, A398P and T435P substitutions (pDONR221-AR-AD-L56P+Tau-1+Tau-5*) was subcloned into a pDEST17 vector using the LP clonase reaction (Thermo Fisher Scientific).

##### eGFP-AR-ΔNLS-Δ21–35

A 507-bp fragment with deletion of residues 21–35 was amplified from pCMV5-FLAG-AR deltaFQNLF^30^ using KOD polymerase (Merck Millipore) and the supplied buffer no. 2. The resulting fragment was purified using AmPure XT (Beckman) before InFusion (Takara Bio) into SalI and AflII-cut and gel-purified peGFPC1-AR ∆NLS plasmid.

##### eGFP-AR-ΔNLS-Tau-1

The A186P, L192P and C238P substitutions were introduced in a step-wise manner into the WT AR sequence encoded in pDONR221-AR-AD-WT using a Quickchange protocol with Pfu Turbo polymerase (Agilent). A 755-bp fragment was amplified from the resulting clone, incorporating the A186P, L192P and C238P substitutions (pDONR221-AR-AD-TAU1), using KOD polymerase (Takara Bio). The resulting fragment was digested with DpnI to remove the template and purified using AmPure XT (Beckman) before InFusion into AflII-BstEII-cut and gel-purified peGFP-C1-ARΔNLS plasmid.

##### eGFP-AR-ΔNLS-Tau-5

The A356P, A398P and T435P substitutions were introduced in a step-wise manner into the WT AR sequence (pDONR221-AR-AD-WT) using a Quickchange protocol with Pfu Turbo polymerase (Agilent). A 1,544-bp fragment was then amplified from the resulting plasmid, incorporating the A356P, A398P and T435P substitutions (pDONR221-AR-AD-TAU-5), using KOD polymerase (Takara Bio). The resulting fragment was digested with DpnI to remove the template and purified using AmPure XT (Beckman) before InFusion into KpnI-cut and gel-purified peGFP-C1-ARΔNLS plasmid.

#### BioID plasmid-generation strategy

Constructs for expression of FLAG-MTID or its fusions with AR WT and 22YtoS were synthesized by Genscript and were either cloned into pcDNA3.1(–) and subsequently cloned into pLenti-CMV-MCS-GFP-SV-puro using XbaI and BamHI to replace GFP or cloned directly into pLenti-CMV-MCS-GFP-SV-puro by Genscript using the same sites. Sequences were codon optimized for mammalian expression and verified by sequencing. pLenti-CMV-MCS-GFP-SV-puro was a gift from P. Odgren (Addgene plasmid no. 73582).

### Experiments in vitro

#### Expression and purification of constructs

WT and mutant AR AD (1–558 aa) were recombinantly produced in *E. coli* Rosetta (DE3) cells that were transformed with pDEST17 plasmid encoding His-AR-AD, as described previously^72^. Briefly, cell cultures at an optical density of 600 nm (OD_600_) of 0.5 were induced with 0.1 mM IPTG at 22 °C overnight. Cells were lysed in PBS buffer and centrifuged. The pellet was solubilized overnight in Tris buffer (20 mM Tris, 500 mM NaCl, 5 mM Imidazole, pH 8) containing 8 M urea and 500 mM NaCl at pH 8. The protein was captured on Nickel columns (His Trap HP, GE Healthcare) and eluted with 500 mM imidazole. After urea removal by dialysis, the His-tag was cleaved by TEV protease at 4 °C overnight. Urea (8 M) was added to cleaved protein before reverse-nickel affinity chromatography to separate noncleaved protein and the His-tag. Protein in the flowthrough was concentrated, filtered and stored at −80 °C. To prevent protein aggregation or instability, an additional purification step was conducted, and the sample was run on a Superdex 200 16/600 column pre-equilibrated with AR AD buffer (20 mM sodium phosphate, 1 mM TCEP pH 7.4). Tau-5* (330–448 aa) was expressed and purified as previously described^23^, and an equivalent protocol was used to express and purify fragment AR AD (441–558 aa).

AR-LBD (663–919 aa) containing an amino-terminal His-tag and encoded in a pET15b plasmid (Addgene no. 89083) was expressed in Rosetta (DE3) cells with 1 mM IPTG at 16 °C overnight. Cells were resuspended in Ni-Wash buffer (25 mM HEPES, 500 mM NaCl, 10% glycerol, 1 mM DTT, 10 μM DHT, 1% Tween-20, 20 mM imidazole at pH 7.4), lysed and centrifuged. Soluble protein was captured by IMAC and eluted with 500 mM imidazole. During an overnight dialysis, His-tag was cleaved by thrombin (GE Healthcare), and the NaCl concentration was reduced to 100 mM. Cleaved protein was captured by cation exchange (GE Healthcare) and eluted with 1 M NaCl gradient. LBD was injected in a Superdex 200 16/600 column pre-equilibrated with 25 mM HEPES, 250 mM NaCl, 10% glycerol, 1 mM TCEP, 10 μM DHT, 1 mM EDTA and 0.5% Tween-20 at pH 7.2.

MED1 IDR (948–1573), encoded in a peTEC plasmid, was a gift from T. Graf. A 3C cleavage site was introduced by Q5 site-directed mutagenesis (New England Biolabs) between mCherry and the MED1 sequence, yielding peTEC-His-mcherry-3C-MED1-IDR plasmid. Protein was expressed in B834 (DE3) cells at 16 °C overnight with 0.1 mM IPTG. Upon cell lysis in Tris buffer with 100 mM NaCl, the soluble cell fraction was injected in a HisTrap HP column, and protein was eluted with 500 mM imidazole. The eluted protein was concentrated and separated by cation exchange chromatography. The collected fractions were cleaved by 3C protease, and MED1 IDR was separated from other protein fragments by size-exclusion chromatography (SEC) with Superdex 200 16/600 column pre-equilibrated with 20 mM sodium phosphate, 100 mM NaCl and 1 mM TCEP at pH 7.4.

RNAPII CTD (1592–1970) was produced in *E. coli* B834(DE3) cells transformed with the pDEST17 plasmid, which encodes H6-3C-RNAPII-CTD. The protein was expressed at 25 °C overnight with 0.1 mM IPTG and extracted from the insoluble cell fraction. The pellet was resuspended in Tris buffer with 8 M urea and loaded on a HisTrap HP column. Captured protein was dialyzed against 50 mM Tris-HCl, 50 mM NaCl and 1 M NaCl at pH 8 and was cleaved by 3C protease overnight at 4 °C. RNAPII CTD was injected in a Superdex 200 16/600 column pre-equilibrated with 20 mM sodium phosphate, 150 mM NaCl, 5% glycerol and 1 mM TCEP at pH 7.4.

AR-LBD, MED1-IDR and RNAPII-CTD fractions from SEC were concentrated, filtered and stored at −80 °C until further use.

#### Turbidity measurements

Protein samples were prepared in AR AD buffer (20 mM sodium phosphate, 1 mM TCEP pH 7.4), with the indicated protein and NaCl concentrations, on ice. Samples were centrifuged at 21,130 r.c.f. for 20 min at 4 °C, and the supernatant was transferred to a quartz cuvette. Phase separation *T*_c_ measurements of protein solutions were monitored by measuring the absorbance of the solutions at 340 nm as a function of temperature on a Cary 100 Multicell UV-vis spectrophotometer, equipped with a Peltier temperature controller, at a heating rate of 1 °C min^–1^. The *T*_c_ values were obtained as the maximum of the first-order derivative of the obtained curves from three independent samples.

#### Protein labeling

For in vitro condensation experiments, proteins were labeled with fluorescent dye instead of being tagged with fluorescent protein, to avoid nonspecific interactions in heterotypic condensates. AR AD and MED1 IDR were fluorescently labeled with Dylight 405 or Alexa Fluor 647, respectively, unless otherwise indicated in the figure legends. LBD and RNAPII-CTD were labeled with Oregon Green 488. In all cases, sulfhydryl-reactive dyes were used, which reacted to the naturally occurring cysteines of the protein, except for RNAPII-CTD in which an N-terminal Cys was added. Protein was labeled according to the manufacturer’s instructions for sulfhydryl-reactive dyes (Thermo Fisher Scientific). Briefly, protein and dye were mixed at a 1:20 ratio in each protein storage buffer, adjusted to pH 7.5 overnight at 4 °C. Then, 1 mM DTT was added to stop the reaction, and protein was separated from free dye with a pre-equilibrated PD-10 column. Protein was concentrated and filtered, and the concentration and conjugation efficiency were analyzed, following the manufacturer’s instructions for sulfhydryl-reactive dyes (Thermo Fisher Scientific).

#### Fluorescence microscopy of in vitro protein condensation

Each protein solution was prepared by adding approximately 1% of equivalent labeled protein. Solutions were stored on ice. Samples were prepared by mixing proteins at the indicated protein concentration with AR AD buffer (20 mM sodium phosphate, 1 mM TCEP pH 7.4) in low binding PCR tubes at RT. Once all proteins were mixed, the phase separation trigger was added: NaCl for AR samples, or Ficoll 70 for transcriptional component samples. Samples were homogenized, and 1.5 μl of sample was transferred into sealed chambers composed of a slide and a PEGylated coverslip sandwiching 3M 300 LSE high-temperature double-sided tape (0.34 mm). Coverslips were PEGylated according to the published protocol^73^. Images were taken using Zeiss LSM 780 Confocal Microscope with a Plan-ApoChromat ×63/1.4 Oil objective lens. Fluorescence recovery after photobleaching (FRAP) experiments were recorded using the same set-up on a 50 μM AR AD sample containing approximately 1% of protein labeled with DyLight 488 dye (Thermo Fisher Scientific) with 500 mM NaCl. The data were analyzed using the EasyFRAP software^74^ to extract the mobile fractions and recovery half-times.

#### NMR experiments

##### Assignment strategy

All NMR experiments were performed at 5 °C (278 K) on either a Bruker 800 MHz (DRX or Avance NEO) or a Bruker Avance III 600 MHz spectrometer, both equipped with TCI cryoprobes, and versions 3.2 and 4.0.8 of TOPSPIN.

A 300 μM ^15^N,^13^C-double-labeled AR AD (441–558 aa) sample in NMR buffer (20 mM sodium phosphate (pH 7.4), 1 mM TCEP, 0.05% (wt:vol) NaN_3_) was used for backbone resonance assignment. The following series of three-dimensional (3D) triple resonance experiments were acquired: HNCO, HN(CA)CO, HNCA, HN(CO)CA, CBCANH and CBCA(CO)NH. Chemical shifts were deposited in the Biological Magnetic Resonance Bank (BMRB) (ID: 51476).

The assignment of AR AD (1–558 aa) was guided by the assignments obtained for the smaller AR fragments that were first studied here (residues 441–558) or previously reported (residues 1–151 (BMRB ID: 25607) and 142–448 (BMRB ID: 51479)). In addition, 3D HNCO and HNCA experiments were acquired for two ^15^N,^13^C-double-labeled AR AD (1–558 aa) samples (25 μM and 100 μM) dissolved in NMR buffer. For the 100 μM sample, additional 3D HN(CA)CO and HN(CO)CACB experiments were also recorded. Three-dimensional experiments were done using 25% non-uniform sampling. Chemical shifts were deposited in the BMRB (ID: 51480).

Backbone resonances of AR WT* were almost identical to those of AR AD (1–558 aa), with only local differences in residues around the position substituted (L26), which were assigned using non-uniform sampled 3D BEST-TROSY HNCO and HNCA experiments^75^ recorded on a 50 μM ^15^N,^13^C-double-labeled WT* AR AD sample dissolved in NMR buffer.

##### Site-specific and residue-type identification of oligomerization

The oligomerization of AR AD was monitored by recording the induced intensity changes on the two-dimensional ^1^H,^15^N correlation spectrum by adding increasing amounts of unlabeled sample on a 25 μM ^15^N-labeled AR AD to reach total concentrations of 57.5, 100.8, 122.5 or 155 μM. Spectra were recorded using 128 scans per increment (with an experimental time of 21 h per spectrum) to ensure that intensities in the regions with weaker signals were quantified properly. Throughout the article, the term oligomer refers to intermolecular complexes formed through weak, and therefore reversible, site-specific interactions between monomers.

##### Helicity studies upon TFE addition

The effect of TFE on 50 μM WT* AR AD and Tau-5* secondary structures were monitored by a series of ^1^H,^15^N correlation spectra and non-uniform sampled 3D BEST-TROSY, HNCO and HNCA experiments recorded in the presence of increasing TFE amounts (0%, 2.5% and 5%).

##### Binding studies

EPI-001 and 1aa binding to Tau-5* was studied by comparing ^15^N chemical shifts in 2D ^1^H,^15^N CP-HISQC^76^ spectra at 37 °C (310 K), using 60 μM Tau-5* in the absence or presence of 60 μM compounds (1:1 ratio). Samples contained NMR buffer (above) at pH 6.6 with 200 mM NaCl and 2% DMSO-d_6_. The CP-HISQC pulse sequence and the pH level of 6.6 were chosen to reduce water exchange of labile amide protons at 37 °C (310 K).

##### Data processing

Data processing was done using qMDD^77^ for non-uniform sampled data, and NMRPipe^78^ for all uniformly collected experiments. Data analysis was performed with CcpNmr Analysis^79^. Helix populations were extracted using the δ2D software^67^.

#### Peptides

FQNLFQ and FQNPFQ synthetic peptides were obtained as lyophilized powders with >95% purity from GenScript with amidated C and acetylated N termini. The lyophilized peptides were solubilized at a final concentration of 5 mM in DMSO. Immediately before each experiment, the stock solutions were diluted to 125 μM in 20 mM HEPES buffer, pH 7.5, with 150 mM NaCl. For aggregation assays, the samples were incubated overnight at 37 °C at 600 r.p.m. agitation. The term aggregate refers to the quasi-irreversible formation of fibrillar species stabilized by strong intermolecular interactions, involving a large conformational change.

#### Synchronous light scattering

Synchronous light scattering was monitored using a JASCO Spectrofluorometer FP-8200. The conditions of the spectra acquisition were: excitation wavelength of 360 nm, emission range from 350 to 370 nm, slit widths of 5 nm, 0.5-nm interval and 1,000 nm min^–1^ scan rate. The peptides were sonicated for 10 min in an ultrasonic bath (Fisher Scientific FB15052) before measurement.

#### Fourier transform infrared spectroscopy

Fourier transform infrared spectroscopy (FT-IR) experiments were performed using a Bruker Tensor 27 FT-IR spectrometer (Bruker Optics) with a Golden Gate MKII ATR accessory. Each spectrum consists of 16 independent scans, measured at a spectral resolution of 4 cm^−1^ within the 1,800–1,500 cm^−1^ range. All spectral data were acquired and normalized using the OPUS MIR Tensor 27 software. Data was afterwards deconvoluted using the Peak Fit 4.12 program. The buffer without peptide was used as a control and subtracted from the absorbance signal before deconvolution.

#### Transmission electron microscopy

The morphology of the aggregated FQNLFQ peptide was evaluated by negative staining using a JEOL JEM-1400Plus Transmission Electron Microscope. Five microliters of peptide solution was placed on carbon-coated copper grids and incubated for 5 min. The grids were then washed and stained with 5 μl of 2% wt/vol uranyl acetate for 5 min. Then, the grids were washed again before analysis. Images and videos were processed with ImageJ.

### Cell imaing

#### Microscopy

PC3 cells were seeded in collagen-I-coated µ-slide four-well glass-bottom plates (Ibidi 80426) at 60% confluency 24 h before transfection. Then, 170 ng of expression vectors encoding WT AR tagged with eGFP (eGFP-AR) or mutant AR proteins were transfected per well using polyethylenimine (PEI) (Polysciences) at a ratio of 1 µg DNA to 3 µl PEI. Four hours after transfection, the medium was changed to RPMI supplemented with 10% charcoal-stripped FBS and cells were cultured for 16 h before imaging. Transiently transfected PC3 cells expressing eGFP-AR were imaged in 3D during 1 min, by taking one image every 15 s, to acquire a baseline readout of AR expression. Cells were then treated immediately with 1 nM of DHT and imaged during 1 h, also by taking an image every 15 s. Time-lapse imaging was performed in an Andor Revolution Spinning Disk Confocal with an Olympus IX81 microscope and a Yokogawa CSU-XI scanner unit. Images were acquired with an Olympus PlanApo N ×60/1.42 Oil objective lens. A stable temperature (37 °C) was maintained during imaging under CO_2_ in a temperature-regulated incubation chamber (EMBL). eGFP was excited with a 488 nm laser, and *Z*-stack images were acquired every 0.45 μm. Time-lapse images were compiled, processed and analyzed with Fiji (ImageJ)^80^. Intensity thresholds were set manually and uniformly to minimize background noise.

FLAG-MTID-AR-WT and PC3 FLAG-MTID-AR-WT-Y22toS cell lines were seeded in 24-well culture plates, on 12-mm sterilized coverslips. The next day, 50 μM biotin (or DMSO for a negative control) and 1 nM DHT were added for 2 h. The culture medium was removed and the cells were washed with PBS. Next, cells were fixed for 15 min with 4% paraformaldehyde. After fixation, cells were washed with PBS and then permeabilized with 0.1% Triton X-100 for 10 min. Coverslips were then washed and blocked with blocking buffer (3% BSA, 0.1%Tween, PBS) for 1 h at 37 °C. Coverslips were incubated with primary antibody—anti-AR (Abcam, ab108341, 1:100)—overnight. The next day, coverslips were washed with PBS, and secondary antibodies were added (1:500): anti-streptavidin antibody conjugated to Alexa Fluor 488 (Thermo Fisher Scientific, S11223) or Alexa Fluor 488-conjugated goat anti-rabbit-IgG (H+L) (Thermo Fisher Scientific, A11008). Fluorescence images were acquired using a Leica TCS SP8 confocal microscope. Images were taken with ×63 oil objectives, and standard LAS-AF software was used.

HEK293T cells in DMEM with 10% FBS were seeded at a density of 40,000 cells per well on glass-bottom chambered coverslips (Ibidi 80827). Sixteen hours later, wells were refreshed with 280 µl seeding medium and transfected with 50 ng of mEGFP expression plasmids, as shown in Extended Data Fig. 2a, using LipoD293 transfection reagent (SignaGen SL100668) according to the manufacturer’s protocol. Forty-eight hours later, wells were refreshed with medium spiked with 10 nM DHT or the equivalent DMSO control (vol/vol). Four hours after treatment, coverslips were imaged on a Zeiss LSM 880 confocal microscope with a Plan-ApoChromat ×63/1.4 Oil DIC objective lens in a CO_2_ incubation chamber set to 37 °C. Images were acquired across two biological replicates.

#### STED microscopy

##### Sample preparation

HEK293T and HeLa eGFP-AR cells in DMEM with 10% FBS were seeded at a density of 40,000 cells per well on glass-bottom chambered coverslips (Ibidi 80827). Sixteen hours later, wells containing HEK293T cells were refreshed with 280 µl seeding medium and transfected with 50 ng of mEGFP expression plasmids, as shown in Fig. 1b, using LipoD293 transfection reagent (SignaGen SL100668), according to the manufacturer’s protocol. Forty-eight hours later, wells were refreshed with medium spiked with 10 nM DHT. Samples were imaged after 4 h of DHT treatment.

LNCaP cells (Clone FGC, ATCC CRL-1740) were seeded in RPMI-1640 5% FBS onto PLL-coated 18-mm no. 1.5 thickness glass coverslips (Sigma P4707, Roth LH23.1) at a density of 100,000 cells per coverslip on a 24-well plate. Sixteen hours later, the media was refreshed and cells were incubated further for another 24 h. For fixation, wells were washed with PBS, then fixed with 1 ml of 4% PFA in PBS for 20 min at room temperature. After a second wash in PBS, cells were permeabilized with 0.5% Triton X-100, PBS (vol/vol) (Sigma 93443) and then stained with anti-AR (AR 441, scbt 7305, 1:50) and STAR 635P secondary antibody (Abberior, ST635P-1001, 1:200). Nuclear translocation of the AR signal was validated by staining LNCaP cells grown in RPMI-1640 5% CSS (Gibco, A3382101), following the same protocol. DNA was counterstained with 1:2,000 Spy555-DNA (Spirochrome, SC201), and samples were mounted onto glass slides with vectashield (Biozol, VEC-H-1900-10).

##### Live-cell STED

HEK293T and HeLa cells were imaged on a Leica Stellaris STED DMI 8 microscope equipped with an okolab incubation chamber set to 37 °C and a constant concentration of CO_2_ (5%). eGFP imaging was performed using a 473-nm stimulation wavelength laser at 20% power and a 592-nm depletion laser at 20% power. Images were taken using a HC PL APO CS2 ×63/1.40 oil objective, with a final resolution of 23 nm pixel^–1^.

##### Stimulated emission depletion fluorescence-lifetime imaging microscopy

Fixed and stained LNCaP cells were imaged on a Leica Stellaris STED DMI 8 microscope. Abberior STAR 635P immunofluorescence imaging was performed using a laser with a stimulation wavelength of 633 nm at 5% power, and a 776-nm depletion laser at 5% power. Images were taken using a HC PL APO CS2 ×63/1.40 oil objective, with a final resolution of 48 nm pixel^–1^. Fluorescence-lifetime imaging microscopy cutoffs and fluorescence-lifetime stimulated emission depletion deconvolution strengths were determined automatically using Leica LAS-X software v2.5.6 to filter background photons with low fluorescence lifetimes (Extended Data Fig. 1d).

#### FRAP assay in live cells

PC3 cells were transfected and prepared for microscopy in identical conditions to those of the live-cell imaging experiments. Before performing FRAP assays, cells were treated with 1 nM DHT. FRAP data for each condition were acquired over the course of approximately 1 h after treatment, and results were combined for each condition because no trend was observed between FRAP data acquired at the beginning versus the end of the hour. FRAP measurements were performed on an Andor Revolution Spinning Disk Confocal microscope with a FRAPPA Photobleaching module and a iXon EMCCD Andor DU-897 camera. Images were taken using a ×100/1.40 Oil U Plan S-Apo objective lens. Pre-bleaching and fluorescence recovery images of the eGFP-AR were acquired using a laser power of 488 nm with an exposure time of 100 ms. Bleaching was done in a 10 × 10 pixel square region of interest (ROI) on top of a droplet, which was repeated five times; the maximum laser power intensity was 488 nm, and the dwell time for bleaching was 40 µs. Twenty pre-bleached images and 200 post-bleached images in total were taken at intervals of 180 ms. Post-bleached images were acquired immediately after the bleaching. Mean gray intensity measurements were quantified in three different ROIs in each FRAP experiment: a bleached region, a background region outside the cells and a region spanning the whole cell were drawn to allow normalization of the gray values. Fiji was used to measure it in each ROI using the plot *z*-axis profile function to extract the intensity data. Exported csv tables were normalized and fitted in EasyFrap software^74^ to extract kinetic parameters, such as T-half and mobile fraction. Double normalization was used to correct for fluorescence bleaching during imaging and for differences in intensity.

### Drug-condensate interactions

#### Drug partition coefficient calculation

Concentrations of EPI-001 in the dense and light phases of WT* AR AD and 8YtoS AR AD were determined using the Agilent Technologies 1200 HPLC instrument, using a Jupiter analytical C4 column from Phenomenex. H2O and ACN:H_2_O (9:1) were used as mobile phases, containing 0.1% TFA.

Samples were prepared on ice in 20 mM sodium phosphate buffer (pH 7.4), 1 mM TCEP and 0.05% (wt/wt) NaN_3_. One equivalent of compound was added to 60 μM of protein from DMSO stocks. The final concentration of DMSO in all samples was 2%. Liquid–liquid phase separation of the protein was induced by adding 1.25 M NaCl, followed by incubation for 5 min at 37 °C and centrifugation at 2,000 r.p.m. for 2 min at 37 °C to separate the light and dense phases. The light phase was transferred to a new microcentrifuge tube, and the dense phase was diluted nine times by adding the buffer containing 4 M urea, which dissolvd the condensates. These solutions were injected in an HPLC system. The corresponding peaks of small molecules were integrated, and concentrations were determined using standard calibration curves that were obtained by measuring four concentrations for each compound.

#### Effect of compounds on AR AD phase separation in vitro

The effects of compounds on AR AD phase separation in vitro were assessed by turbidity (see ‘Turbidity measurements’) and microscopy (see ‘Fluorescence microscopy of in vitro protein condensation’). The samples contained 25 μM WT* AR AD with 1 molar equivalent of the indicated compounds in 20 mM sodium phosphate buffer (pH 7.4), 1 mM TCEP, 0.05% (wt/vol) NaN_3_, 1 M NaCl and 2% DMSO.

### Experiments in cells

#### Luciferase reporter assay in HEK293T

HEK293T cells were co-transfected with an androgen-response element (ARE)-luciferase construct containing a luciferase reporter gene under the control of three AREs (kindly provided by the M. Pennuto lab), along with an empty vector, an AR-expression vector (pEGFP-C1-AR or AR V7) or different mutants in the presence or absence of DHT. HEK293T cells were maintained in DMEM with 10% charcoal-stripped FBS during the assay. Transfections were carried out using PEI, and cells were treated with vehicle or 1 nM DHT 24 h after transfection. Cell extracts were prepared 48 h after transfection, when eGFP-AR mutants are mostly localized to the nucleus, and assayed for luciferase activity using the Promega luciferase detection kit. Luciferase activities were normalized to co-transfected β-galactosidase activity^81^.

#### Luciferase reporter assays in LNCaP

PSA(6.1 kb)-luciferase, V7BS3-luciferase and AR-V7 plasmids and transfections of cells have been described previously^24,52,53,82^. PSA(6.1 kb)-luciferase reporter plasmid (0.25 μg well^–1^) was transiently transfected into LNCaP cells that were seeded in 24-well plates. Twenty-four hours after transfection, cells were pretreated with compounds for 1 h prior to the addition of 1 nM R1881 and incubation for an additional 24 h. For the V7BS3-luciferase reporter, an expression vector encoding AR-V7 (0.05 μg well^–1^) and a filler plasmid (pGL4.26, 0.45 μg well^–1^) were transiently co-transfected with V7BS3-luciferase reporter plasmid (0.25 µg well^–1^) into LNCaP cells in 24-well plates. After 24 h, the cells were treated with the indicated compounds for additional 24 hours. Transfections were completed under serum-free conditions using Fugene HD (Promega). Luciferase activity was measured for 10 s using the Luciferase Assay System (Promega) and normalized to total protein concentration determined by the Bradford assay. Validation of consistent levels of expression of AR-V7 protein was done using western blot analyses.

#### Proliferation assays

LNCaP cells (Clone FGC, ATCC CRL-1740) in RPMI-1640 with 5% FBS were seeded at a density of 4,000 cells well^–1^ into 96-well flat-bottom plates (Greiner, 655075) that had been pre-coated with poly-l-lysine (Sigma P4707). Sixteen hours later, triplicate wells were refreshed with 100 µl of seeding medium spiked with 7× serial dilutions of EPI-001 from 200 µM (Selleckchem lot no. S795502), 7× serial dilutions of 1ae from 50 µM, or DMSO control, at a constant DMSO concentration of 0.5% (vol/vol). Ninety-six hours later, wells were washed with 200 µl PBS and then fixed with 100 µl of 4% PFA in PBS for 20 min at room temperature. After fixation, LNCaP nuclei in each well were counterstained using 100 µl of Hoechst 33342 (Abcam ab228551), diluted to 1:4,000 in PBS, for 20 min at room temperature. After a final wash in PBS, plates were imaged using a Celldiscoverer 7 microscope equipped with a ×20 air objective. Twenty-five tile regions (5 × 5 tiles) were imaged for each technical replicate well (triplicate wells for each dose and compound). Data were acquired across two biological replicates performed on different weeks.

To compare the antiproliferative effects of 1ae and enzalutamide in LNCaP and LNCaP95 cells, LNCaP cells (5,000 cells well^–1^) were plated in 96-well plates in their respective media plus 1.5% dextran-coated charcoal (DCC)-stripped serum. LNCaP cells were pretreated with the compounds for 1 h before they were treated with 0.1 nM R1881 for an additional 3 d. Proliferation and viability were measured using the Alamar blue cell viability assay, following the manufacturer’s protocol (Thermo Fisher Scientific). LNCaP95 cells (6,000 cells well^–1^) were seeded in 96-well plates in RPMI plus 1.5% DCC for 48 h before the addition of compounds and incubation for an additional 48 h. BrdU incorporation was measured using BrdU Elisa kit (Roche Diagnostics).

#### Quantitative real-time polymerase chain reaction

Target primer sequences are listed in Supplementary Table 6. LNCaP cells (Clone FGC, ATCC CRL-1740) in RPMI-1640 with 5% FBS were seeded at a density of 300,000 cells well^–1^ in 6-well plates. Sixteen hours later, wells were refreshed with seeding medium spiked with either EPI-001 or 1ae at doses roughly corresponding to the IC_50_ and IC_10_ values calculated from proliferation assays, indicated in Extended Data Fig. 7a, and 0.5% vol/vol DMSO control. After 6 or 24 h, the medium was removed and cells were collected using 300 µl of TRIzol reagent (Thermo Fisher Scientific 15596026) in each well. RNA was then extracted using a Zymo DirectZol Micro kit (Zymo R2062), according to the manufacturer’s protocol. cDNA was synthesized using 1 µg of RNA, random hexamer primers, and the RevertAid First Strand cDNA Synthesis kit (Thermo Fisher Scientific K1622). cDNA collected from LNCaP cells treated with either EPI-001 or 1ae at each dosage, and time point, were then probed for transcript targets on 384-well plates using the SYBR Green master mix (Thermo Fisher Scientific A25777), and a QuantStudio 7 real-time qPCR machine. For calculation of the fold change (2^−ΔΔCt^ method), Ct values from target regions were normalized to Ct values from control regions from the treatment sample, and were then normalized to the DMSO sample. Data were collected from three biological replicates performed on different days.

#### RNA-seq data generation

LNCaP cells (Clone FGC, ATCC CRL-1740) in RPMI-1640 with 5% FBS were seeded at a density of 300,000 cells well^–1^ into 6-well plates. Sixteen hours later, wells were refreshed with seeding medium spiked with either EPI-001 or 1ae at the doses indicated in Fig. 6c and 0.5% vol/vol DMSO control. After 6 or 24 h, medium was removed and cells were collected using 300 µl of TRIzol (Thermo Fisher Scientific 15596026) in each well. RNA was then extracted using a Zymo DirectZol Micro kit (Zymo R2062), according to the manufacturer’s protocol. Total RNA-seq libraries were then prepared using 1 µg of RNA from each sample and the KAPA RNA HyperPrep Kit with RiboErase (Roche KR1351), according to the manufacturer’s protocol, with ten amplification cycles. Libraries were sequenced on a NovaSeq 6000 with paired-end reads of 100 bp, with a read depth of 50 million fragments per library. Three libraries from three corresponding biological replicates were prepared for each treatment (time, dosage, and compound).

#### Western blot

To compare the levels of AR expression, cells were washed and collected in PBS ×1 and lysed in RIPA buffer ×1 (Thermo Fisher Scientific, 88900) containing phosphatase and protease inhibitors (Roche). Lysates were centrifuged at 15,000*g* to separate soluble and pellet fractions. Total protein was quantified using a BCA assay (Pierce Biotechnology). Proteins were resolved by 4–12% gradient Bis-Tris SDS–PAGE (Invitrogen NP0323), transferred to PVDF membranes and blocked with 5% non-fat milk in TBST for 1 h at room temperature with shaking. The membranes were incubated with the following antibodies: anti-GAPDH (Abcam, ab59164, 1:2,000) and anti-AR (Abcam, ab108341, 1:2,000) as well as RD-680-conjugated anti-mouse (LI-COR, 926-68072, 1:10,000) and CW-800-conjugated anti-rabbit (LI-COR, 926-32211, 1:10,000) secondary antibodies. Membrane fluorescence was read with the Odyssey CLx infrared imaging system (LI-COR).

To determine the effect of 1ae treatment on AR levels, LNCaP cells (ATCC, CRL-1740) were seeded in RPMI-1640 (Thermo Fisher Scientific, 11875093) supplemented with 5% FBS (Thermo Fisher Scientific, 1835030) and 1% penicillin–streptomycin (Thermo Fisher Scientific, 15140122) at a density of 150,000 cells well^–1^ into six-well plates (Thermo Fisher Scientific, 140685). Forty-eight hours later, cycloheximide (Sigma, C7698) was added to a final concentration of 50 µg ml^–1^ and incubated for 3 h before incubation with 1ae (at the indicated concentrations) for 21 h. Cells were washed in PBS (Thermo Fisher Scientific, 11835030) and lysed in RIPA buffer (Thermo Fisher Scientific, 88900) containing protease inhibitor (Abcam, ab274282). Lysates were centrifuged at 15,000*g* to separate soluble and insoluble fractions. Soluble protein was quantified using a BCA assay (Pierce Biotechnology, 23225). Protein extracts (10–70 µg) were electrophoresed in a Bolt 8% Bis-Tris gel (Invitrogen, NW00085BOX) and transferred with PVDF transfer stacks (Invitrogen, PB5210). Membranes were blocked with 3% non-fat milk in TBST for 1 h at room temperature with shaking. Afterwards, membranes were incubated with the following antibodies: anti-GAPDH (Abcam, ab59164, 1:1,000), anti-AR (Abcam, ab108341, 1:1,000), 800-CW-conjugated anti-rabbit (LI-COR, 926-32211, 1:10,000) and RD-680-conjugated anti-mouse (LI-COR, 926-68072, 1:10,000). Imaging was done using the Odyssey CLx, and protein-band intensity was quantified with ImageJ.

#### Lentiviral production for FLAG-BioID-AR cell lines

FLAG-MTID, FLAG-AR-WT-MTID or FLAG-22YtoS-MTID were subcloned from pcDNA3.1(–) (Genscript) into pLenti-CMV-MCS-GFP-SV-puro (Addgene no. 73582) by replacing GFP using XbaI-BamHI digestion. Vectors were co-transfected with lentiviral packaging plasmid vectors REV (cat. no. 12253), RRE (cat. no. 12251) and VSV-G (cat. no. 8454) into 293T cells with PEI (Sigma-Aldrich). Two days after transfection, virus-containing medium was collected and filtered through a 0.45-µm low-protein-binding filtration cartridge. The virus-containing medium was used to directly infect LNCaP/PC3 cells in the presence of polybrene (8 µg ml^–1^) for 48 h, before 1 μg ml^–1^ puromycin was introduced for 72 h to select for stable cell lines. pMDLg/pRRE was a gift from D. Trono (Addgene plasmid no. 12251; http://n2t.net/addgene:12251; RRID: Addgene_12251). pCMV-VSV-G was a gift from B. Weinberg (Addgene plasmid no. 8454; http://n2t.net/addgene:8454; RRID: Addgene_8454). pRSV-Rev was a gift from D. Trono (Addgene plasmid no. 12253; http://n2t.net/addgene:12253; RRID: Addgene_12253).

#### BioID–MS

Prior to BioID experiments, MTID-containing stable cell lines were generated through lentiviral infection and puromycin selection. They were subsequently grown in RPMI-1640 medium modified with l-glutamine without phenol red or biotin (United States Biological, R9002-01) with 10% (vol/vol) charcoal-stripped FBS for 48 h. Cells were seeded, and the next day, 50 μM biotin (IBA; 2-1016-002) and 1 nM DHT were added for 2 h. For small-molecule inhibitors, cells were pretreated for 1 h with either EPI-001 or 1ae, then for 2 h with DHT and biotin. For MS, cells were collected through trypsinization, washed two times in PBS and snap-frozen on dry ice. Cell pellets were lysed in modified RIPA buffer (1% TX-100, 50 mM Tris-HCl, pH 7.5, 150 mM NaCl, 1 mM EDTA, 1 mM EGTA, 0.1% SDS, 0.5% sodium deoxycholate and protease inhibitors) on ice and treated with 250 U benzonase (Millipore), and biotinylated proteins were isolated using streptavidin-sepharose beads (GE Healthcare). Proteins were washed in ammonium bicarbonate and digested with trypsin. Mass spectrometry was performed in the IRB Barcelona Mass Spectrometry and Proteomics facility, as described previously^83^. Data were analyzed using SAINTq^84^.

#### Proximity ligation assay

Protein–protein interactions were studied using a Duolink In Situ Orange Starter Kit Mouse/Rabbit (Sigma, DUO92102), following the manufacturer’s protocol. Briefly, transduced prostate cancer cells were seeded in coverslips and cultured overnight. The next day, they were treated with 50 μM biotin and 1 nM DHT for 2 h or were pretreated initially with small-molecule inhibitors. Slides were washed with cold 1× PBS and fixed in 4% paraformaldehyde for 15 min, washed in PBS and permeabilized using 0.1% Triton X-100 for 10 min and washed then blocked with blocking buffer (3% BSA, 0.1% Tween in PBS) for 1 h at 37 °C. The coverslips were blocked with Duolink Blocking Solution in a pre-heated humidified chamber for 30 min at 37 °C. Primary antibodies to the following proteins were added and incubated overnight at 4 °C: androgen receptor (ER179(2)) (Abacam, no. ab108341, 1:200), Nup153 (Abacam, QE5, no. ab24700, 1:200), Med1 (Abacam, no. ab64965, 1:200) and ARID1a/BAF250A (Cell Signalling, no. 12354, 1/200). Coverslips were then washed with 1× wash buffer A and subsequently incubated with the two PLA probes (1:5, diluted in antibody diluents) for 1 h, then the ligation-ligase solution for 30 min, and the amplification-polymerase solution for 100 min in a pre-heated humidified chamber at 37 °C. Before imaging, slides were washed with 1× wash buffer B and mounted with a cover slip using Duolink In Situ Mounting Medium with DAPI. Fluorescence images were acquired using a Leica TCS SP8 confocal microscope. Images were taken with ×100 oil objectives, using standard LAS-AF software.

#### Gene expression analysis

To analyze tumor gene expression, tumors were flash frozen, and ~100-mg samples were pulverized under liquid nitrogen. Samples were added to 1 ml TRIzol (Invitrogen) and homogenized using a FastPrep-24 tissue homogenizer (MP Biomedicals). Total RNA was extracted using the RNeasy Micro Kit (Qiagen), cleaned using the DNase I Kit, amplification grade (MilliporeSigma), and reverse transcribed using the High-Capacity RNA-to-cDNA Kit (Thermo Fisher Scientific), according to the manufacturers’ protocols. Diluted cDNA and Platinum SYBR Green qPCR SuperMix-UDG with ROX (Invitrogen) were combined with gene-specific primers. Transcript quantification was completed using a QuantStudio 6 RT–qPCR machine, and calculation of the mean normalized expression of target transcripts was done using the 2^−ΔΔCt^ method using the housekeeping gene *SDHA*. To analyze gene expression in PC3 cells expressing AR V7, 2 × 10^5^ cells were plated in duplicate in 6-well plates. After 48 h, total RNA was extracted using the SV Total RNA Isolation System (Promega), following the manufacturer’s instructions.

#### Determination of AR levels in LNCaP treated with 1ae and CHX

LNCaP cells (ATCC, CRL-1740) were seeded in RPMI-1640 (Thermo Fisher Scientific, 11875093) supplemented with 5% FBS (Thermo Fisher Scientific, 1835030) and 1% penicillin–streptomycin (Thermo Fisher Scientific, 15140122) at a density of 150,000 cells well^–1^ into 6-well plates (Thermo Fisher Scientific, 140685). Forty-eight hours later, cycloheximide (Sigma, C7698) was added to a final concentration of 50 µg ml^–1^ and incubated for 3 h before incubation with 1ae (at the indicated concentrations) for 21 h. Cells were washed in PBS (Thermo Fisher Scientific, 11835030) and lysed in RIPA buffer (Thermo Fisher Scientific, 88900) containing protease inhibitor (Abcam, ab274282). Lysates were centrifuged at 15,000*g* to separate the soluble and insoluble fractions. Soluble protein was quantified using the BCA assay (Pierce Biotechnology, 23225). Protein extracts (10–70 µg) were electrophoresed in a Bolt 8% Bis-Tris gel (Invitrogen, NW00085BOX) and transferred using PVDF transfer stacks (Invitrogen, PB5210). Membranes were blocked with 3% non-fat milk in TBST for 1 h at room temperature with shaking. Afterwards, membranes were incubated with the following antibodies: anti-GAPDH (ab59164, 1:1,000), anti-AR (ab108341, 1:1,000), 800-CW-conjugated anti-rabbit (LI-COR 926-32211, 1:10,000) and anti-mouse (LI-COR 926-68072, 1:10,000). Imaging was conducted with Odyssey CLx, and protein-band intensity was quantified with ImageJ.

### Molecular dynamics simulation

A molecular dynamics simulation of the AR Tau-5_R2_R3_ region (residues L391–G446, capped with ACE and NH_2_ groups) in the presence of 1aa was performed using GROMACS 2019.2 (refs. ^85,86^), patched with PLUMED v2.6.0 (ref. ^87^) as described previously^51^ and compared with previously reported simulation results of Tau-5_R2_R3_ in the presence of EPI-002 (ref. ^51^). In brief, an explicit solvent simulation was performed in a cubic box with a length of 7.5 nm and neutralized with a salt concentration of 20 mM NaCl by 8 Na^+^ ions and 5 Cl^−^ ions. The AR Tau-5_R2−R3_ protein was parameterized using the a99SB-disp force field; water molecules were parameterized with the a99SB-disp water model^88^. 1aa was parameterized using the GAFF^89^ for ligand forcefield parameters. The replica exchange with solute tempering (REST2) algorithm^90^ was used to enhance conformational sampling. Sixteen replicas were run in parallel using a temperature ladder ranging from 300–500 K, with all protein and ligand atoms selected as the solute region. Tau-5_R2−R3_ with 1aa was simulated for 5.2 μs per replica, respectively, for a total simulation time of 83.2 μs. Convergence of simulated properties was assessed by a comparison of the conformational sampling of each simulated replica, as previously reported^51^, and statistical errors were calculated using a blocking analysis following Flyvbjerg and Peterson^91^. We define an intermolecular contact between a ligand and a protein residue as occurring in any frame where at least one heavy (non-hydrogen) atom of that residue is found within 6.0 Å of a ligand heavy atom. To calculate a simulated *K*_D_ value for each compound, we defined the bound population of each ligand as the fraction of frames with at least one intermolecular contact between a ligand and Tau-5_R2_R3_.

### Statistics and reproducibility

#### Statistical analysis

Pairwise comparisons shown in Figs. 1h, 2d,g,3e,g, 4k, 5d,f,g,i and 6j,k and Extended Data Figs. 4d,g,h,j,k5d, 6c,j,k,7f and 8a,b were performed using a Student’s *t*-test or Mann–Whitney *U* test in base R or python. Differences were considered significant when adjusted *P* values were lower than 0.0001 (****), 0.001 (***), 0.01 (**) or 0.05 (*).

#### AR ΔNLS image analysis in live cells

A custom-made macro in Fiji was developed to quantify the total number and the size of AR condensates into the cytoplasm as a function of time (Fig. 3e and Extended Data Fig. 4d,g,j,k). This macro also quantifies the total area of the cytosol to normalize the results.

The macro creates *z*intensity projections of the 3D stacks. A manual step of drawing a ROI was integrated into the macro to select the nuclei to be removed so that only the cytoplasm area would be kept for the detection and quantification of the AR condensates. After filtering and thresholding steps, the cytosol area was segmented and quantified. Then a mathematical operation was done between the resulting mask of the cytosol without the nuclei and the *z* maximum intensity projection data to detect and quantify the total number and the area of AR condensates in the cytosol. The quantification was done at three time points after DHT exposure.

#### AR nuclear translocation rate analysis

A custom-made macro in Fiji was developed to quantify the mean gray intensity value in the nuclei area over time (Fig. 2b). The macro creates a *z*-sum projection of the 3D stacks from the time-lapse to improve the quantitativeness of the results. A stackreg plugin is used in the macro to register and correct the *xy*movement of the cells over time; there is a manual step that involves drawing the nuclei area and the cytoplasm area to extract automatically the mean gray values of these ROIs over time.

#### Luciferase reporter assay in HEK293T

For the transcriptional activity assay, reported in Fig. 2g, a general linear model was used to compare differences in the log-transformed ARE-luciferase/β-galactosidase ratio between groups of interest using biological replicates as covariates. For clarity of representation, ARE-luciferase/β-galactosidase ratios are shown in the original scale.

#### Analysis of FRAP data for cell experiments

Mean intensities of bleached areas were corrected both for bleaching due to imaging over time and background noise. The corresponding calculations were performed with EasyFrap by calculating the fluorescence intensity over time (*I*(*t*)). Obtained values were further normalized to the initial fluorescence by dividing *I*(*t*) by the mean gray value of the initial pre-bleaching acquisition images.

#### Granularity analysis

Image analysis was assisted by a macro written at the IRB ADMCF. An individual segmentation mask was obtained for each nucleus (excluding the nucleoli) by simple median filtering, background subtraction and local thresholding. Nuclei exhibiting an insufficient or overly strong level of expression were excluded manually, and the s.d. of the intensity was estimated inside the remaining nuclei in the original images. For the granularity analysis, reported in Fig. 2d, the s.d. values were compared across groups by linear regression. The relationship between s.d. and mean intensity was also compared across groups, and is reported in Extended Data Fig. 3a, by fitting a linear model with the s.d. as the response variable and taking the mean intensity, the group, the interaction between the group and the mean intensity and the biological replicate as explanatory variables. The slope between mean intensity and s.d. was compared for every experimental group against the control through the interaction term of the linear model. Dunnett’s multiple-comparisons correction was used to compare the linear effects of several experimental groups with a common control. Images of HEK239T cells transfected with mEGFP plasmids, described in Extended Data Fig. 2a, were analyzed using ZEN Blue version 3.2. Image fields were segmented for nuclear regions using automatic thresholding (Otsu thresholding) on the mEGFP channel, and the resulting objects were analyzed for mean intensity and standard deviation of pixels. As above, nuclear clustering (or granularity) was assayed as the s.d. of pixels, and nuclear GFP concentration as the mean intensity of pixels in the corresponding nuclear object. Measurements were exported for data wrangling in R to create the plots shown in Extended Data Fig. 2b. Eight to ten image fields were used to assay nuclei from each condition (transfection and treatment).

#### LNCaP dose–response curves

Raw LNCaP nuclei counts from proliferation experiments, assayed as objects detected by automatic Otsu thresholding on Hoechst signal from image fields from each well (aggregate of 25 tile regions), were used to construct dose–response curves for EPI-001 and 1ae (Fig. 6c). Segmentation was performed using ZEN Blue version 3.2 on image data acquired from two biological replicates. Nuclear counts from each well were exported and processed using the DRC package in R^92^ to create the dose–response curves shown in Fig. 6c. Data were modeled with a three-parameter log-logistic function (lower limit 0), and the resulting fit was used to calculate IC_50_ and IC_10_ values for EPI-001 and 1ae (Fig. 6c).

#### In vitro droplet image analysis

For in vitro droplet analysis of AR AD in images with multiple components in Fig. 1, droplets were identified by applying a threshold (3,255) to the channel sum image using Fiji. AR-AD intensity within the identified droplets larger than 0.1 μm across three image fields was extracted and plotted in Fig. 1h. The graph from Fig. 1j was obtained by normalizing each channel’s intensity from the plotted profile of a section of a representative droplet using Fiji. Droplets in Fig. 3f were identified applying a threshold (3,255) to the channel sum image using Fiji.

#### τ-STED image analysis

Composites acquired in τ-STED mode (Extended Data Fig. 1b) were exported as .tiff image fields using Leica LAS-X version 2.5.6 and analyzed using a custom Fiji pipeline (Extended Data Fig. 1c), available at https://github.com/BasuShaon/AR-RNA-seq-STED. In brief, the DNA counterstain was first used to identify and threshold nuclear objects. Clusters within nuclear objects were then detected using the rolling ball algorithm, with the size of the rolling ball set to eight times the limit of detection (48 nanometers), according to a standard protocol^93^. This enabled detection of nuclear AR clusters for cells imaged with the same τ-deconvolution strength. Nuclear AR clusters were pooled from 7 LNCaP nuclei and analyzed for mean intensity and size as indicated in Extended Data Fig. 1b.

#### ChromLogD determination

ChromLogD values were experimentally determined as a measure of hydrophobicity of the 1aa family of compounds. The experimental evaluation was subcontracted to Fidelta. Values of ChromLogD were calculated from the equation:

$${\rm{ChromLogD}}=0.0857\times {\rm{CHI}}-2,$$

In which CHI is a chromatographic hydrophobicity index. CHI values were determined from gradient retention times at pH = 7.4. Chromatograms were measured using the Agilent 1100 HPLC instrument, using a Luna C18 analytical column from Phenomenex. For mobile phases, 50 mM ammonium acetate (in H_2_O) and ACN were used, with 0.1% TFA. The chromatographic separation was optimized for a 5-min run, by using a linear gradient from 0% to 100% ACN in the first 3 min.

#### RNA-sequencing data pre-processing

Paired-end sequencing reads were first quality checked using FASTQC and then aligned to the *Homo sapiens* genome build hg19 using STAR aligner v2.7.5a (ref. ^94^) with standard settings. The first and fourth columns in ReadsPerGene.out.tab STAR output files (GeneIDs and reverse strand reads) were used to build raw count matrices for each sample library.

#### Differential expression analysis

Differential expression analysis between treatment conditions was conducted using the DESeq2 R/bioconductor package, a statistical tool that uses shrinkage estimates to compute fold changes^95^. First, raw count matrices from sample libraries were merged into a single object using the ‘DESeqDataSetFromHTSeqCount’ function with the design set to the treatment condition (time, compound and dosage). The merged count matrix was then fitted to the DESeq statistical model using the ‘DESeq’ function. The fit and merged matrix was then reduced using a variance-stabilizing transformation, ‘vst,’ to visualize principal components one and two (Extended Data Fig. 7b). The fold change values in gene expression and corresponding significance scores were then extracted using the ‘results’ function by querying a contrast between any two conditions (Supplementary Data Table 3). Cutoffs of |log_2_(FC)| > 1 and *P*< 1 × 10^–10^ were used to determine differentially expressed genes in a given contrast (Fig. 6d).

#### Gene set enrichment analysis

Gene set enrichment analysis was performed using R/bioconductor packages fgsea and DOSE^96,97^. Ranked gene lists were first constructed using log_2_(FC) values for the genes in any given DESeq2 contrast by sorting log_2_(FC) values in descending order and filtering out duplicate entries. Ranked lists were then analyzed for the enrichment of 50 hallmark gene sets (collection H) obtained from the molecular signature database msigDB, maintained by the Broad Institute, using the ‘plotEnrichment’ and ‘plotfgseaRes’ functions in fgsea and the ‘GSEA’ function in DOSE (nperm = 10,000, *P*value cutoff < 0.05).

Besides the commonly used gene set enrichment plot for a queried gene pathway (Extended Data Fig. 7c,e) we also show enrichment scores for the top 10 negatively and top 10 positively enriched pathways as a dotplot, with gradient scaling to the normalized enrichment score (red, positive NES; blue, negative NES) and size proportional to the statistical significance (*P*_adj_) of the calculated enrichment (Fig. 6e).

For the analysis of AR V7 mutants (Extended Data Fig. 3j–l), AR target genes upregulated in PC3 cells were identified using a composite of AR GSEA data sets^96^.

#### Mean expression value of genes in hallmark gene sets

Line plots for mean expression values of genes were adapted from refs. ^98,99^. In brief, reads from the merged count matrix were normalized using the equation log_2_(normalized DESeq counts + 1) to create a log_2_ normalized count matrix (Supplementary Data Table 4). Normalized counts for each gene in the matrix were then *z*-score-scaled using the ‘scale_rows’ function from the pheatmap R package. Code integrated with DESeq2 available at https://github.com/BasuShaon/AR-RNA-seq-STED. Values of the genes from the below gene sets were then plotted as indicated in Fig. 6f and Extended Data Fig. 7d as a function of the concentration of EPI-001 and 1ae. MsigDB hallmark pathway set H: http://www.gsea-msigdb.org/gsea/msigdb/genesets.jsp?collection=H. EPI-001 negative DEGs: KLK3, ADAM7, TBX15, FKBP5, PGC, LAMA1, ELL2, CHRNA2, STEAP4, DSC1, UGT2B28, TNS3, BMPR1B, SLC38A4, EAF2, TTN, SLC15A2, CCDC141, HPGD, TMEM100, MAF, F5, TRGC1.

### Reporting summary

Further information on research design is available in the Nature Portfolio Reporting Summary linked to this article.

## Online content

Any methods, additional references, Nature Portfolio reporting summaries, source data, extended data, supplementary information, acknowledgements, peer review information; details of author contributions and competing interests; and statements of data and code availability are available at 10.1038/s41594-023-01159-5.

### Supplementary information

Supplementary Information Synthesis and characterization of small molecules.

Reporting Summary

Peer Review File

Supplementary Video 1 Fluorescence time-lapse video of eGFP-AR condensates in PC3 cells. Cells were treated with 1 nM DHT and imaged with spinning disk microscopy. Scale bar: 10 µm.

Supplementary Video 2 Fluorescence time-lapse video of eGFP-AR-ΔNLS condensates in PC3 cells. Cells were treated with 1 nM DHT. Scale bar: 10 µm.

Supplementary Video 3 Fluorescence time-lapse video of PC3 cells expressing eGFP-AR or the indicated YtoS mutant. Cells were treated with 1 nM DHT and imaged with spinning disk microscopy. Scale bar: 10 µm.

Supplementary Table 1 BioID-MS data corresponding to Fig. 2 and Extended Data Fig. 3. The Bait tab contains the bait IDs used for SAINTq analysis, and the SAINTq output tab is the analysis output. The data summary is a pivot table generated from the output data. The ‘WT Top 75 GO’ and ‘Y22S Top 75 GO’ tabs are the output data from STRING analysis of the top 75 most abundant proteins with a BFDR ≤ 0.02 and a FC ≥ 3 from the *t*_DHT_ = 60 min samples.

Supplementary Table 2 BioID-MS data corresponding to Fig. 5 and Extended Data Fig. 6. The Bait tab contains the bait IDs used for SAINTq analysis and the SAINTq output tab is the analysis output. The data summary is a pivot table generated from the output data. Fold change in small-molecule inhibitor-treated versus DMSO-treated LNCaP MTID-AR-WT cells. The GO tab refers to most depleted proteins versus DMSO with a *P* ≤ 0.05 and a FC ≥ −1.5.

Supplementary Table 3 RNA-Seq DESeq2 log_2_(FC) values by contrasts indicated in Fig. 6d–f and Extended Data Fig. 7.

Supplementary Table 4 RNA-Seq DESeq2 raw count matrix and normalized count matrix used to calculate expression values plotted in Fig. 6 and Extended Data Fig. 7d.

Supplementary Table 5 Gene level RNA-Seq data analysis, GSEA datasets, AR up genes in PC3 AR-V7 and source data used to produce Extended Data Fig. 3j–l.

Supplementary Table 6 Sequences of primers and synthetic genes.

### Source data

Source Data Fig. 1 Statistical source data.

Source Data Fig. 2 Statistical source data.

Source Data Fig. 3 Statistical source data.

Source Data Fig. 4 Statistical source data.

Source Data Fig. 5 Statistical source data.

Source Data Fig. 6 Statistical source data.

Source Data Extended Data Fig. 1 Statistical source data.

Source Data Extended Data Fig. 2 Statistical source data.

Source Data Extended Data Fig. 3 Statistical source data.

Source Data Extended Data Fig. 4 Statistical source data.

Source Data Extended Data Fig. 5 Statistical source data.

Source Data Extended Data Fig. 6 Statistical source data.

Source Data Extended Data Fig. 7 Statistical source data.

Source Data Extended Data Fig. 8 Statistical source data.

Source Data Fig. 3 and Extended Data Figs. 3 and 6 Unprocessed western blots.

## Data Availability

The RNA-sequencing data have been deposited in the NCBI GEO database (https://www.ncbi.nlm.nih.gov/geo/) under accession codes GSE206853 and GSE232849. The NMR assignments for constructs 441–558 and the AR AD have been deposited in the BMRB (https://bmrb.io/) with accession codes 51476 and 51480, respectively. The molecular dynamics simulation trajectories, GROMACS input files and analysis code have been deposited in Zenodo (10.5281/zenodo.8210256). Source data are provided with this paper.

## Code availability

Molecular dynamics simulation analysis code is freely available from Github: https://github.com/paulrobustelli/Basu_Rational_Optimization_AR_inhibitors_NSMB_2023. Code for gene expression analyses is also freely available from Github: https://github.com/BasuShaon/AR-RNA-seq-STED.
