# Supplementary material for: Rational optimization of a transcription factor activation domain inhibitor
Source: Nat Struct Mol Biol. 2023 Dec 4;30(12):1958–69. doi: 10.1038/s41594-023-01159-5 (PMC10716049; doi:10.1038/s41594-023-01159-5)
Supplement: Supplementary file 1 — Synthesis and characterization of small molecules. [file 41594_2023_1159_MOESM1_ESM.pdf]

nature structural & molecular biology

Article https://doi.org/10.1038/s41594-023-01159-5

Rational optimization of a transcription factor activation domain inhibitor

In the format provided by the authors and unedited

Supplementary Document 1: synthesis and characterization of small molecules

The synthetic design was to have a two-carbon atom linkers and kept the functionality untouched. Our approach was designed to check the best geometry between the two nearly symmetrical functionalized fragments of the EPI-001 molecule. We planned four different geometrical scaffolds: compounds **1** displaying a linear arrangement, compounds **2** with a *cis*-configuration of the double bond, compounds **3** with a *trans* alkene configuration and compounds **4** with a flexible alkyl linker (Figure 1).

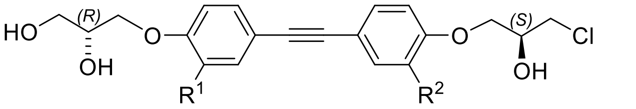

R^1 = H, R^2 = H, **1aa**
R^1 = H, R^2 = Me, **1ab**
R^1 = H, R^2 = OMe, **1ac**
R^1 = H, R^2 = F, **1ad**
R^1 = H, R^2 = Ph, **1ae**
R^1 = H, R^2 = *t*-Bu, **1af**
R^1 = Me, R^2 = H, **1ba**
R^1 = Me, R^2 = Me, **1bb**

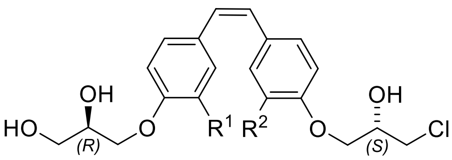

R^1 = H, R^2 = H, **2aa**
R^1 = H, R^2 = Me, **2ab**
R^1 = Me, R^2 = H, **2ba**

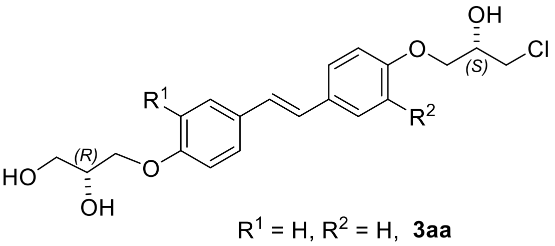

R^1 = H, R^2 = H, **3aa**
R^1 = H, R^2 = Me, **3ab**

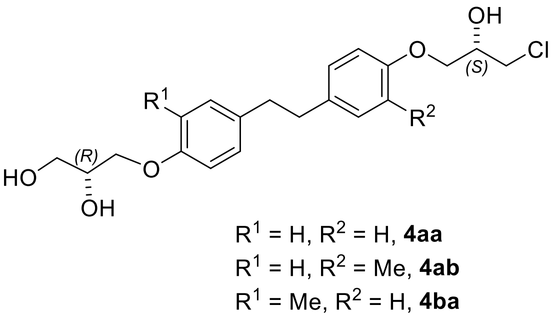

R^1 = H, R^2 = H, **4aa**
R^1 = H, R^2 = Me, **4ab**
R^1 = Me, R^2 = H, **4ba**

Figure 1. Structures and numbering of the new families of compounds synthetized.

The retrosynthetic analysis for the target compounds is shown in Scheme 1. We envisaged that the four geometrical arrangements (**1-4**) could be prepared from the key acetylenic intermediates **5** which, in turn, would be accessible through two Sonogashira reactions involving trimethylsilyl acetylene and the two iodophenol fragments **7a** and **7b**. The protected diol and the masked chlorohydrin functionalities would be introduced by substitution reactions of the appropriate glycydol derivatives with the corresponding phenols. Both functionalities would be uncovered in the last step. This approach should permit the preparation of a large family of acetylenic compounds **1** by simply selecting the corresponding iodophenols. The other geometrical arrangements **2-4** would be synthetized by partial or total reduction of the final alkynes or their intermediates.

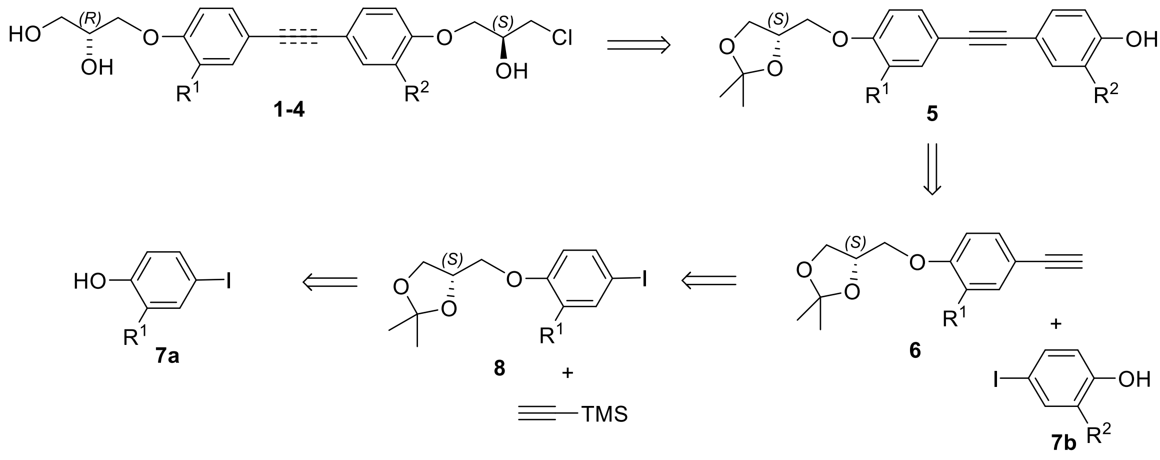

Scheme 1. Retrosynthetic analysis of compounds 1-4.

Structure Activity Relationship (SAR)

The initial acetylene analogue without substitution at the aromatic ring (**1aa**) was synthesized as shown in Scheme 2. The known tosilate **10** was prepared from (*S*)-(2,2-dimethyl-1,3-dioxolan-4-yl)methanol according to literature procedures[1] The starting 4-iodophenol **7a** was reacted with **10** to give intermediate **8a**. The two-carbon linker was installed by a Sonogashira reaction with trimethylsilylacetylene affording compound **9a** in excellent yield[2]. The trimethylsilyl group was removed in basic media and **6a** was used without purification into a second Sonogashira reaction to introduce the second aromatic ring to give **5aa**. Then, the key intermediate **5aa** was reacted with epoxide **11** to give epoxide **12aa** in excellent yield**.** Treatment of **12aa** with cerium trichloride in acetonitrile opened the epoxide and hydrolyzed the acetal affording the final linear product **1aa**.

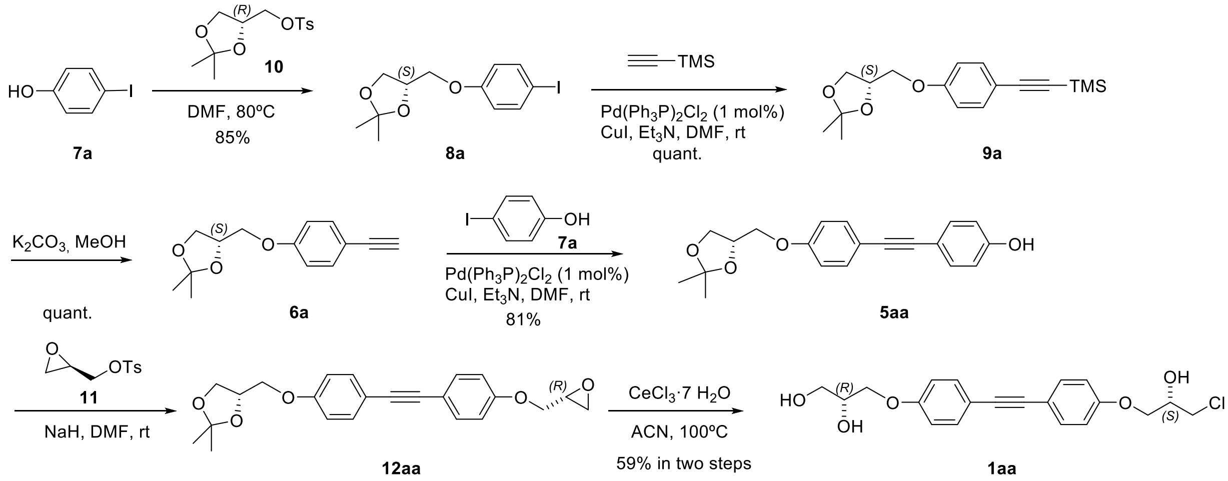

Scheme 2. Synthesis of target compound 1aa.

The partial hydrogenation of epoxide **12aa** using poisoned palladium catalyst ($Pd/BaSO_4$/quinoline)[3] afforded the *cis* alkene **13aa** with variable amounts of the completely hydrogenated product **16aa**. In this reduction the amount of quinoline was critical and the

reaction was difficult to reproduce. Using transfer hydrogenation conditions ($Pd_2(dba)_3$/dppe ($HCOOH/NEt_3$)[4], the reaction afforded the *cis*-olefin but the epoxide was ring-opened yielding the diol. With the *cis*-epoxide **13aa** in hand, compound **2aa** was obtained by treatment with cerium trichloride. Isomerization of epoxide **13aa** to the *trans*-isomer by UV light afforded the epoxide **14aa** with *E* configuration which, after the deprotection and ring opening afforded compound **3aa** (Scheme 3). Both olefinic compounds could be obtained in a more convenient way from the final acetylenic chlorohydrine **1aa**. Transfer hydrogenation of **1aa** using $Pd_2(dba)_3$/dppe as catalyst afforded the (*Z*)-alkene **2aa**. The corresponding (*E*) isomer **3aa** was appropriately prepared by isomerization, heating in a solution of formic acid in dioxane.

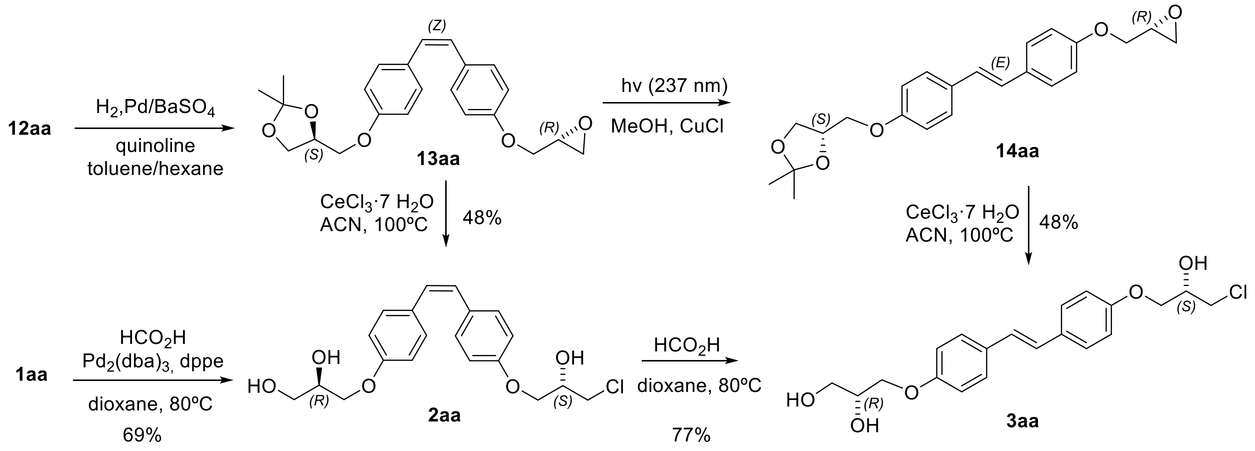

Scheme 3. Synthesis of olefinic compounds 2aa and 3aa.

The flexible-linker compound **4aa** was obtained uneventfully by palladium-catalyzed hydrogenation of intermediate **5aa**. Treatment of **15aa** with tosyl glycidol **11** furnished epoxide **16aa**. As before, acetal deprotection and epoxide ring opening of **16aa** produced the final product **4aa** (Scheme 4). It could be also obtained by direct hydrogenation of **1aa** with Pd/C.

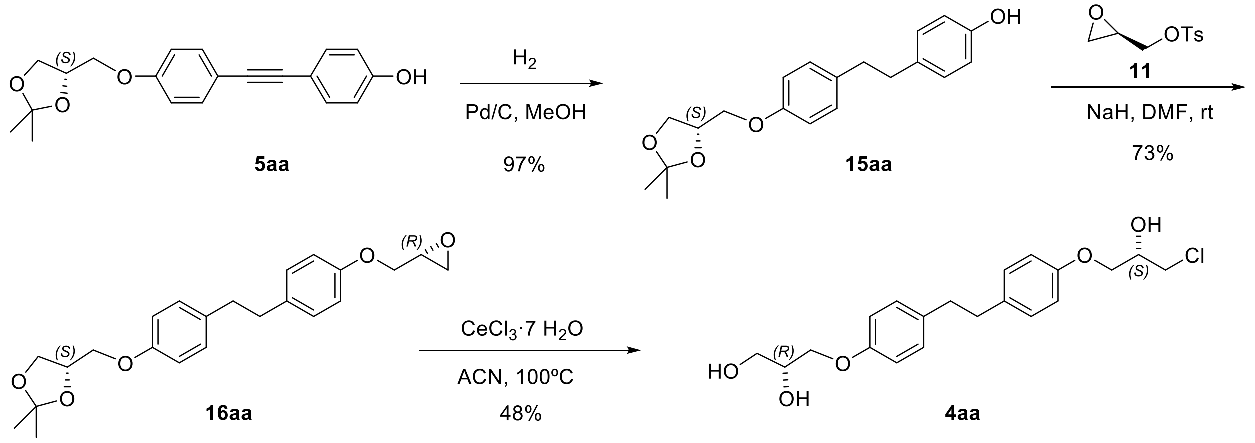

Scheme 4. Synthesis of compound 4aa with a flexible alkyl linker.

The four linker-expanded analogues without further substitution at the aromatic ring (**1-4aa**) were biologically evaluated. The triple bond analogue **1aa** and the (*Z*)-alkene **2aa** gave the best results, and, somewhat surprisingly, even better than ralaniten (EPI-002). To increase the hydrophobicity of the compounds and to double-check the best geometrical arrangements, we designed a second set of compounds (**1-4ab**), with a methyl group in the ring bearing the chlorohydrin group. The synthesis followed the previous schemes but using 2-methyl-4-iodophenol **7b** in the second Sonogashira reaction. The results of the AR transcriptional activity assay showed that compound **1ab** with the linear arrangements had the lowest IC_{50} value. Therefore, the acetylenic core was selected as the best scaffold for the preparation of the second generation of compounds. A family of acetylenic compounds **1ax** varying the group in the *ortho* position to the chlorhydrin was then synthetized following the same synthetic route (see experimental part for details). The following groups were tested: methoxy (**1ac**), fluor (**1ad**), phenyl (**1ae**) and *tert*-butyl (**1af**). Moreover, three compounds with a methyl group at the *ortho* position of the other aromatic ring (**1ba, 2ba** and **4ba**) and a compound with two methyl groups, one in each aromatic ring (**1bb**) were also synthetized (Figure 1).

Materials and methods

Chemistry. General Methods.

All chemical reagents and analytical-grade solvents were obtained from commercial sources and used without further purification. All reactions were monitored by thin layer chromatography (TLC) using silica gel on aluminum sheets (Merck Kieselgel 60). Compound purification was achieved either using an automated chromatography system (PuriFlash® 430, Interchim) and silica (Merck Kieselgel 60, 230−400 mesh ASTM) and the eluents indicated in the procedures for each compound. Melting points (Mp) were determined using a Büchi capillary apparatus and were not corrected. Optical rotations ($[\alpha]_D$) were measured at room temperature (25 ºC) in a 1 mL cell using a Jasco P-2000 iRM800 polarimeter. Concentration is expressed in g/100 mL.

Biological experiments were performed on compounds with a purity of at least 95%. All compounds were routinely checked by ^1H and ^{13}C NMR (Varian Mercury 400 MHz). Chemical shifts (δ) were referenced to internal solvent resonances and reported relative to tetramethylsilane (TMS). The coupling constants (J) are reported in Hertz (Hz). The following abbreviations are used to define multiplicities: s (singlet), d (doublet), t (triplet), q (quartet), dd (doublet of doublets), dq (doublet of quartets), m (multiplet), quint (quintuplet), bs (broad signal). The IR spectra were recorded on a Thermo Nicolet 6700 FT-IR spectrometer using an ATR system, KBr discs or NaCl discs (Film). HRMS spectra were recorded on in an LC/MSD-TOF G1969A (Agilent Technologies) from the *Centres Científics i Tecnològics* of the University of Barcelona or at the Mass spectrometry facility of the IRB Barcelona. The

known (*R*)-(2,2-dimethyl-1,3-dioxolan-4-yl)methyl 4-methylbenzenesulfonate (**10**) and of (*R*)-oxiran-2-ylmethyl 4-methylbenzenesulfonate (**11)** were prepared according to standard procedures.

Abbreviations: DMF: dimethyl formamide. ACN: acetonitrile. Ts: *p*-toluensulfonyl. TMS: trimethylsilyl. dppe: ethylenebis(diphenylphosphine).

Synthesis of compound (1aa)

The sequence described in Scheme 4 was followed.

(*S*)-4-((4-iodophenoxy)methyl)-2,2-dimethyl-1,3-dioxolane (8a) A suspension of Cs_2CO_3 (2.58 g, 7.92 mmol) and 4-iodophenol (871 mg, 3.96 mmol) in 5 mL of anh. DMF was heated under nitrogen up to 80 ºC for 20 min. A solution of (*R*)-(2,2-dimethyl-1,3-dioxolan-4-yl)methyl 4-methylbenzenesulfonate (**10**, 1.05 g, 7.92 mmol) in dry DMF (5 mL) was added over 15 minutes. Then, the reaction was stirred overnight and finally quenched by addition of 100 mL of a saturated solution of $KHCO_3$. The resulting aqueous layer was extracted with EtOAc (2×100 mL) and this organic layer was washed with 10% v/w $CuSO_4$ (2×100 mL). The organic layer was dried over anh. $MgSO_4$, evaporated and purified by column chromatography (40 g silica column equilibrated with 2% Et_3N in hexane and eluted with a gradient of 0-20% EtOAc in hexane) to obtain 1.12 g (85% yield) of the desired product **8a.** $[\alpha]_D$ (*c* 0.98, $CHCl_3$) = +6.95. IR (Film) ν_{max} = 2985, 2933, 1585, 1486, 1371, 1243, 1055 cm^{-1}. ^1H NMR (400 MHz, $CDCl_3$) δ: 7.58 – 7.52 (m, 2H), 6.73 – 6.65 (m, 2H), 4.50 – 4.42 (m, 1H), 4.16 (dd, *J* = 8.5, 6.4 Hz, 1H), 4.01 (dd, *J* = 9.5, 5.4 Hz, 1H), 3.90 (dd, *J* = 9.8, 5.8 Hz, 1H), 3.88 (dd, *J* = 8.6, 5.8 Hz, 1H), 1.45 (bs, 3H), 1.40 (bs, 3H) ppm. ^{13}C NMR (101 MHz, $CDCl_3$) δ: 158.6, 138.4, 117.1, 110.0, 83.4, 74.0, 69.0, 66.9, 26.9, 25.5 ppm. HRMS (ESI): m/z $[M + H]^+$ calculated for $C_{12}H_{16}IO_3$ 335.0139; found: 335.0136.

(*S*)-((4-((2,2-dimethyl-1,3-dioxolan-4-yl)methoxy)phenyl)ethynyl)trimethylsilane (9a). (*S*)-4-((4-iodophenoxy)methyl)-2,2-dimethyl-1,3-dioxolane (**8a**, 727 mg, 2.18 mmol) was dissolved in 25 mL of anh. DMF in a Schlenk flask and CuI (21 mg, 0.11 mmol), $Pd(Ph_3P)_2Cl_2$ (78 mg, 0.11 mmol), trimethylsilylacetylene (0.46 mL, 3.41 mmol) and 16 mL of Et_3N were added to the solution under N_2 atm. The reaction was stirred for 1.5 h (the solution turns black), diluted with EtOAc (50 mL) and washed with brine (100 mL). The aqueous layer was extracted with EtOAc (2×150 mL) and the combined organic layers were dried over anh. $MgSO_4$, filtered and concentrated under vacuum. The crude was purified by column chromatography (40 g silica column equilibrated with 2% Et_3N in hexane and eluted with a gradient of 0-30% EtOAc in hexane) to obtain 685 mg (100% yield) of the expected product **9a** as a brown oil. IR (ATR-FTIR): ν_{max} = 2956, 2360, 2155, 1505, 838 cm^{-1}. ^1H NMR (400

MHz, $CDCl_3$) δ: 7.42 – 7.36 (m, 2H), 6.86 – 6.79 (m, 2H), 4.51 – 4.41 (m, 1H), 4.15 (dd, *J* = 8.5, 6.4 Hz, 1H), 4.04 (dd, *J* = 9.5, 5.4 Hz, 1H), 3.97 – 3.85 (m, 2H), 1.45 (bs, 3H), 1.40 (bs, 3H), 0.23 (s, 9H) ppm. ^{13}C NMR (101 MHz, $CDCl_3$) δ: 158.8, 133.6, 115.9, 114.5, 110.0, 105.2, 92.8, 74.1, 68.9, 66.9, 26.9, 25.5, 0.2 ppm. HRMS (ESI): m/z $[M + H]^+$ calculated for $C_{17}H_{25}O_3Si$ 305.1567; found: 305.1568.

(*S*)-4-((4-ethynylphenoxy)methyl)-2,2-dimethyl-1,3-dioxolane (6a). Potassium carbonate (107 mg, 0.78 mmol) was added to a solution of (*S*)-((4-((2,2-dimethyl-1,3-dioxolan-4-yl)methoxy)phenyl)ethynyl)trimethylsilane (**9a**, 197 mg, 0.65 mmol) in MeOH (2 mL). The solution was stirred at r.t. for 1.5 h. The reaction mixture was partitioned between DCM (10 mL) and H_2O (10 mL) and the aqueous phase was extracted with DCM (2×10 mL). The combined organic phases were dried over anh. $MgSO_4$, filtered and evaporated to obtain **6a** (197 mg, 100%) as an orange solid. The product was used without further purification. Mp = 43 - 44 ºC. $[\alpha]_D$ (*c* 1.02, $CHCl_3$) = +11.53. IR (ATR-FTIR): ν_{max} = 3282, 2982, 2939, 2913, 2891, 2097, 1602, 1507, 1244, 1049, 835 cm^{-1}. ^1H NMR (400 MHz, $CDCl_3$) δ: 7.44 – 7.39 (m, 2H), 6.87 – 6.82 (m, 2H), 4.51 – 4.42 (m, 1H), 4.16 (dd, *J* = 8.5, 6.4 Hz, 1H), 4.05 (dd, *J* = 9.5, 5.4 Hz, 1H), 3.94 (dd, *J* = 9.5, 5.8 Hz, 1H), 3.89 (dd, *J* = 8.5, 5.8 Hz, 1H), 3.00 (s, 1H), 1.46 (bs, 3H), 1.40 (bs, 3H) ppm. ^{13}C NMR (101 MHz, $CDCl_3$) δ: 159.0, 133.7, 114.8, 114.6, 109.9, 83.6, 76.1, 74.0, 68.9, 66.8, 26.9, 25.4 ppm. HRMS (ESI): m/z $[M + H]^+$ calculated for $C_{14}H_{17}O_3$: 233.1172; found: 233.1171.

(*S*)-4-((4-((2,2-dimethyl-1,3-dioxolan-4-yl)methoxy)phenyl)ethynyl)phenol (5aa). The starting material **6a** (173 mg, 0.75 mmol) was dissolved in THF (1 mL) under N_2 atmosphere and CuI (2.8 mg, 0.02 mmol), $Pd(Ph_3P)_2Cl_2$ (10 mg, 0.02 mmol), 4-iodophenol (164 mg, 0.75 mmol) and 1 mL of Et_3N were added. The resultant solution was stirred at r.t. for 18 h. The reaction mixture was then filtered through a Celite® pad and evaporated. The crude was purified by column chromatography on 12 g silica column equilibrated with 2% Et_3N in hexane and eluted with a gradient of 0-50% EtOAc in hexane. Compound-containing fractions were evaporated to obtain 196 mg (81%) of the **5aa** as an orange solid. Mp = 130 - 131 ºC. $[\alpha]_D$ (*c* 0.72, $CHCl_3$) = +7.22. IR (ATR-FTIR): ν_{max} = 3305, 3216, 2913, 1728, 1177 cm^{-1}. ^1H NMR (400 MHz, $CDCl_3$) δ: 7.47 – 7.36 (m, 4H), 6.90 – 6.83 (m, 2H), 6.82 – 6.76 (m, 2H), 4.52 – 4.47 (m, 1H), 4.18 (dd, *J* = 8.5, 6.4 Hz, 1H), 4.07 (dd, *J* = 9.6, 5.4 Hz, 1H), 3.99 – 3.89 (m, 2H), 1.48 (bs, 3H), 1.42 (bs, 3H) ppm. ^{13}C NMR (101 MHz, $CDCl_3$) δ: 158.5, 155.8, 133.2, 133.0, 116.3, 115.6, 114.7, 110.0, 88.2, 87.9, 74.0, 68.9, 66.9, 26.9, 25.5 ppm. HRMS (ESI): m/z $[M + H]^+$ calculated for $C_{20}H_{21}O_4$ 325.1434; found: 325.1438.

(*S*)-2,2-dimethyl-4-((4-((4-(((*R*)-oxiran-2-yl)methoxy)phenyl)ethynyl)phenoxy)methyl)-1,3-dioxolane (12aa). A suspension of NaH (60% dispersion in oil, 105 mg, 2.62 mmol) in 1 mL of anh. DMF was prepared. A solution of (*S*)-4-((4-((2,2-dimethyl-1,3-dioxolan-4-yl)methoxy)phenyl)ethynyl)phenol (**5aa**, 307 mg, 1.31 mmol) in 3 mL of anh. DMF was

added dropwise and the resulting mixture was stirred at rt for 15 min. Then, a solution of (*R*)-oxiran-2-ylmethyl 4-methylbenzenesulfonate (**11**, 448 mg, 1.96 mmol) in 1 mL of anh. DMF was added to the previous suspension and the mixture was stirred at 40 ºC overnight. The reaction was quenched by addition of 2 mL of NH_4Cl saturated solution and the resulting aqueous layer was diluted with H_2O (15 mL) and extracted with EtOAc (3×20 mL). The combined organic extracts were washed with 10% v/w $CuSO_4$ (2×20 mL), dried over anh. $MgSO_4$, filtered and evaporated to give **12aa**. The crude was used in the following step without further purification (456 mg). Mp = 90 - 91 ºC. $[\alpha]_D$ (*c* 1.01 $CHCl_3$) = +6.46. IR (KBr): ν_{max} = 2923, 1605, 1516, 1242, 836 cm^{-1}. ^1H NMR (400 MHz, $CDCl_3$) δ: 7.47 – 7.40 (m, 4H), 6.91 – 6.84 (m, 4H), 4.48 (quint, *J* = 5.8 Hz, 1H), 4.24 (dd, *J* = 11.0, 3.1 Hz, 1H), 4.17 (dd, *J* = 8.5, 6.4 Hz, 1H), 4.07 (dd, *J* = 9.5, 5.4 Hz, 1H), 4.00 – 3.87 (m, 3H), 3.36 (ddt, *J* = 5.7, 4.1, 2.8 Hz, 1H), 2.92 (dd, *J* = 4.9, 4.1 Hz, 1H), 2.77 (dd, *J* = 4.9, 2.6 Hz, 1H), 1.47 (bs, 3H), 1.41 (bs, 3H) ppm. ^{13}C NMR (101 MHz, $CDCl_3$) δ: 158.5, 158.4, 133.0, 133.0, 116.4, 116.2, 114.7, 114.7, 109.9, 88.2, 88.1, 74.0, 68.9, 68.9, 66.8, 50.1, 44.7, 26.9, 25.5 ppm. HRMS (ESI): m/z $[M + H]^+$ calculated for $C_{23}H_{25}O_5$ 381.1697; found 381.1697.

(*R*)-3-(4-((4-((*S*)-3-chloro-2-hydroxypropoxy)phenyl)ethynyl)phenoxy)propane-1,2-diol (1aa). (*S*)-2,2-dimethyl-4-((4-((4-(((*R*)-oxiran-2-yl)methoxy)phenyl)ethynyl)phenoxy)methyl)-1,3-dioxolane (**12aa**, 66 mg, 0.17 mmol) was dissolved in ACN (3 mL). Then, $CeCl_3 \cdot 7H_2O$ (160 mg, 0.43 mmol) was added and the mixture refluxed at 110 ºC overnight. The reaction mixture was allowed to cool down to rt and evaporated. The paste was dissolved in MeOH, filtered through a Celite® pad and evaporated. The crude was purified by column chromatography (12 g silica column, eluted with a gradient of 30-100% EtOAc in hexane) to yield compound **1aa** (38 mg, 59% yield in two steps) as a white solid. Mp = 141 - 142 ºC. $[\alpha]_D$ = (*c* 0.58, $CHCl_3$/MeOH 1:1) = –0.12. IR (KBr): ν_{max} = 3351, 2924, 1517, 1248, 1037, 832 cm^{-1}. ^1H NMR (400 MHz, CD_3OD) δ: 7.43 – 7.39 (m, 4H), 6.97 – 6.93 (m, 4H), 4.17 – 4.05 (m, 4H), 4.01 – 3.94 (m, 2H), 3.77 (dd, *J* = 11.3, 4.9 Hz, 1H), 3.71 – 3.63 (m, 3H) ppm. ^{13}C NMR (101 MHz, CD_3OD) δ: 160.3, 160.0, 133.8, 133.8, 117.5, 117.2, 115.8, 115.7, 88.8, 88.7, 71.7, 70.9, 70.4, 70.2, 64.1, 46.7 ppm. HRMS (ESI): m/z $[M + H]^+$ calculated for $C_{20}H_{22}ClO_5$ 377.1150; found 377.1149.

Synthesis of compound 2aa

The sequences of Scheme 5 were followed.

(*S*)-2,2-dimethyl-4-((4-((*Z*)-4-(((*R*)-oxiran-2-yl)methoxy)styryl)phenoxy)methyl)-1,3-dioxolane (13aa). In a 25 mL round bottom flask with a magnetic stirrer, (*S*)-2,2-dimethyl-4-((4-((4-(((*R*)-oxiran-2-yl)methoxy)phenyl)ethynyl)phenoxy)methyl)-1,3-dioxolane (**12aa**, 350 mg, 0.92 mmol) was dissolved in a 1:1 mixture of hexanes and toluene (8 mL). Quinoline

(0.11 mL, 0.92 mmol) and Pd/$BaSO_4$ (7 mol%) were added to the starting material and the suspension was put under H_2 (balloon). Stirring was kept for 3 h and the crude was then filtered through a Celite® pad. The solvent was removed under vacuum and the product was purified by column chromatography (12 g silica column, equilibrated with 2% Et_3N in hexane and eluted with a gradient of 0-100% EtOAc in hexane). The product was isolated as a mixture (*Z* isomer and starting epoxide) that was not further purified. ^{1}H NMR (400 MHz, $CDCl_3$) δ: 7.20 – 7.16 (m, 4H), 6.80 – 6.75 (m, 4H), 6.45 (s, 2H), 4.47 (quint, *J* = 5.9 Hz, 1H), 4.22 - 4.14 (m, 2H), 4.04 (dd, *J* = 9.5, 5.4 Hz, 1H), 3.96 – 3.88 (m, 3H), 3.35 (m, 1H), 2.90 (dd, *J* = 5.0, 4.1 Hz, 1H), 2.75 (dd, *J* = 4.9, 2.6 Hz, 1H), 1.46 (s, 3H), 1.40 (s, 3H) ppm. ^{13}C NMR (101 MHz, $CDCl_3$) δ: 157.7, 157.6, 130.7, 130.5, 130.2, 130.2, 128.7, 128.6, 114.5, 114.4, 109.9, 74.2, 68.9, 68.8, 67.0, 50.3, 44.9, 27.0, 25.5 ppm. HRMS (ESI): m/z $[M + H]^+$ calculated for $C_{23}H_{27}O_5$ 383.1859; found 383.1858.

(*R*)-3-(4-((*Z*)-4-((*S*)-3-chloro-2-hydroxypropoxy)styryl)phenoxy)propane-1,2-diol (2aa). *Procedure A.* (*Z*)-2,2-dimethyl-4-((4-(4-(oxiran-2-yl)methoxy)styryl)phenoxy)methyl)-1,3-dioxolane (**13aa**, 73 mg, 0.19 mmol) was dissolved in 5 mL of ACN and $CeCl_3·7H_2O$ (179 mg, 0.48 mmol) was added. The suspension was refluxed at 100 ºC overnight. After this time, the mixture was concentrated, redissolved in MeOH and filtered through a Celite® pad. The solvent was removed under vacuum and the crude was purified by column chromatography (eluted with a gradient of 0-10% MeOH in DCM). The final product was isolated as a white solid (36 mg, 48% yield) that turned out to be a mixture of isomers (75 % of *Z* and 25 % of *E*).

Procedure B. A flame-dried Schlenk tube was charged with the starting alkyne (*R*)-3-(4-((4-((*S*)-3-chloro-2-hydroxypropoxy)phenyl)ethynyl)phenoxy)propane-1,2-diol (**1aa**, 43 mg, 0.11 mmol), $Pd_2(dba)_3$ (5.4 mg, 5 mol%), dppe (2.3 mg, 5 mol%) and 0.2 mL of dioxane. The Schlenk was purged with N_2 and the suspension was stirred at room temperature for 15 min. Then, 9 µL (0.23 mmol) of HCO_2H were injected and the reaction was heated to 80 ºC for 2 h. After removal of the solvent under vacuum, the crude was purified by column chromatography (eluted with a gradient of 30-100% EtOAc in hexane) to afford the *Z* alkene (30 mg, 69%) as a major product (with 10% of the *E* alkene). $[\alpha]_D$ (*c* 0.39, DMSO) = −2.39. IR (ATR-FTIR): ν_{max} = 3363, 2929, 2874, 2364, 1603, 1507, 1242, 1034, 832, 734 cm^{-1}. ^{1}H NMR (400 MHz, CD_3OD) δ: 7.17 – 7.13 (m, 4H), 6.83 – 6.79 (m, 4H), 6.45 (s, 2H) 4.14 – 4.08 (m, 1H), 4.06 – 3.99 (m, 3H), 3.97 – 3.92 (m, 2H), 3.76 (dd, *J* = 11.3, 5.0 Hz, 1H), 3.71 – 3.61 (m, 3H) ppm. ^{13}C NMR (101 MHz, CD_3OD) δ: 159.4, 159.1, 131.8, 131.5, 131.1, 131.1, 129.6, 129.4, 115.3, 115.3, 71.8, 71.0, 70.3, 70.0, 64.2, 46.7 ppm. HRMS (ESI): m/z $[M + H]^+$ calculated for $C_{20}H_{24}ClO_5$ 379.1312; found 379.1310.

<u>Synthesis of compound (3aa)</u>

The sequences of Scheme 5 were followed

(*S*)-2,2-dimethyl-4-((4-((*E*)-4-(((*R*)-oxiran-2-yl)methoxy)styryl)phenoxy)methyl)-1,3-dioxolane (14aa). A mixture of the *Z* and the *E* isomers (**13aa** and **14aa**) with a small amount of the totally reduced product (**16aa**) (37 mg) was dissolved in 3 mL of MeOH and irradiated with a set of 237 nm wavelength lamps in the Rayonet® reactor in the presence of CuCl (3 mg) in a quartz flask. After 1 h, the solvent was concentrated and the crude was purified by column chromatography (4 g silica column, equilibrated with 2% Et_3N in hexane and eluted with a gradient of 0-30% EtOAc in hexane) to obtain 15 mg of the *E* isomer (**14aa**) (with some totally reduced product (**16aa**)). ^{1}H NMR (400 MHz, $CDCl_3$) δ: 7.44 – 7.40 (m, 4H), 6.93 (s, 2H), 6.92 – 6.88 (m, 4H), 4.49 (quint, *J* = 5.9 Hz, 1H), 4.24 (dd, *J* = 11.0, 3.2 Hz, 1H), 4.18 (dd, *J* = 8.5, 6.4 Hz, 1H), 4.08 (dd, *J* = 9.5, 5.4 Hz, 1H), 4.00 – 3.89 (m, 3H), 3.36 (dddd, *J* = 5.7, 4.1, 3.2, 2.7 Hz, 1H), 2.92 (dd, *J* = 4.9, 4.1 Hz, 1H), 2.77 (dd, *J* = 4.9, 2.7 Hz, 1H), 1.47 (s, 3H), 1.41 (s, 3H) ppm. ^{13}C NMR (101 MHz, $CDCl_3$) δ: 158.2, 158.1, 131.1, 131.0, 127.6, 127.6, 126.5, 126.4, 115.0, 114.9, 109.9, 74.2, 69.0, 69.0, 67.0, 50.3, 44.9, 27.0, 25.5 ppm. HRMS (ESI): m/z $[M + H]^+$ calculated for $C_{23}H_{27}O_5$ 383.1853; found 383.1850.

(*R*)-3-(4-((*E*)-4-((*S*)-3-chloro-2-hydroxypropoxy)styryl)phenoxy)propane-1,2-diol (3aa). *Procedure A.* (*S*)-2,2-dimethyl-4-((4-((*E*)-4-(((*R*)-oxiran-2-yl)methoxy)styryl)phenoxy)methyl)-1,3-dioxolane (**14aa**, 15.3 mg, 0.04 mmol) was dissolved in 1 mL of acetonitrile. Then, $CeCl_3 \cdot 7H_2O$ (37.7 mg, 0.1 mmol) was added. The suspension was refluxed at 100 ºC overnight. After this time, the mixture was concentrated, re-dissolved in MeOH and filtered through a Celite® pad. The solvent was removed under vacuum and purified by column chromatography (eluted with a gradient of 0-10% MeOH in DCM) to obtain the final product (7.2 mg, 48% yield).

Procedure B. Compound **2aa** (or a mixture of *Z* and *E* isomers **2aa** and **3aa**) (35 mg, 0.09 mmol) was dissolved in 0.2 mL of dioxane and treated with aqueous formic acid (30 µL, 25 % in water, 0.19 mmol) at 80 ºC for 18 h. Then the solvent was evaporated and the resulting crude was chromatographed (0-100% EtOAc in hexane and AcOEt/MeOH 90:10 in a 4 g column) to yield 27 mg (77%) of **3aa** as a white solid. $[\alpha]_D$ (*c* 0.45, MeOH) = –6.37. IR (ATR-FTIR): ν_{max} = 3358, 2930, 1604, 1510, 1457, 1246, 1175, 1032, 832 cm^{-1}. ^{1}H NMR (400 MHz, CD_3OD) δ: 7.46 – 7.43 (m, 4H), 6.97 (s, 2H), 6.96 – 6.92 (m, 4H), 4.16 – 4.10 (m, 1H), 4.09 – 4.04 (m, 3H), 4.00 – 3.94 (m, 2H), 3.78 (dd, *J* = 11.3, 4.9 Hz, 1H), 3.72 – 3.63 (m, 3H) ppm. ^{13}C NMR (101 MHz, $CDCl_3$) δ: 158.2, 157.8, 130.3, 129.9, 127.4, 126.0, 125.7, 114.8, 114.7, 69.9, 69.6, 69.0, 68.6, 62.7, 46.7 ppm. HRMS (ESI): m/z $[M + H]^+$ calculated for $C_{20}H_{24}ClO_5$ 379.1307; found 379.1302.

Synthesis of compound (4aa)

The sequences of Scheme 6 and the direct reduction of (**1aa**) were followed.

(*S*)-4-(4-((2,2-dimethyl-1,3-dioxolan-4-yl)methoxy)phenethyl)phenol (15aa). To a solution of (*S*)-4-((4-((2,2-dimethyl-1,3-dioxolan-4-yl)methoxy) phenyl)ethynyl)phenol (**5aa**, 103 mg, 0.32 mmol) in 8 mL of MeOH and 1.5 mL of DCM, 34 mg of Pd over C were added. The mixture was purged with N_2 and with H_2 and stirred under nitrogen (balloon) at rt overnight. The resulting suspension was then filtered through a Celite® pad, washed with DCM and evaporated to obtain 101 mg (97% yield) of **15aa**. Mp = 116 - 118 ºC. IR (ATR-FTIR): ν_{max} = 2990, 2923, 2847, 1611, 1510, 1452, 1370, 1241, 1212, 1050, 1031, 823 cm^{-1}. ^1H NMR (400 MHz, CD_3OD) δ: 7.05 – 7.00 (m, 2H), 6.96 – 6.91 (m, 2H), 6.85 – 6.78 (m, 2H), 6.68 – 6.64 (m, 2H), 4.45 – 4.39 (m, 1H), 4.13 (dd, *J* = 8.4, 6.5 Hz, 1H), 3.95 (dd, *J* = 5.4, 4.3 Hz, 2H), 3.84 (dd, *J* = 8.4, 6.2 Hz, 1H), 3.66 (qd, *J* = 11.3, 5.2 Hz, 1H), 2.80 – 2.71 (m, 4H), 1.41 (s, 3H), 1.36 (s, 3H) ppm. ^{13}C NMR (101 MHz, CD_3OD) δ: 156.9, 153.9, 153.9, 134.6, 134.58, 134.0, 134.0, 129.7, 129.5, 115.3, 114.5, 109.9, 109.9, 74.2, 69.0, 67.0, 37.4, 37.4, 26.9, 25.5 ppm. HRMS (ESI): m/z $[M + H]^+$ calculated for $C_{20}H_{25}O_4$ 329.1747; found: 329.1756.

(*S*)-2,2-dimethyl-4-((4-(4-(((*R*)-oxiran-2-yl)methoxy)phenethyl)phenoxy)methyl)-1,3-dioxolane (16aa). A suspension of NaH (60% dispersion in oil, 25 mg, 0.61 mmol) in 0.5 mL of anh. DMF was prepared and cooled down to 0 ºC. A solution of (*S*)-4-(4-((2,2-dimethyl-1,3-dioxolan-4-yl)methoxy)phenethyl)phenol (**15aa**, 101 mg, 0.31 mmol) in 1 mL of anh. DMF was added dropwise and the resulting mixture was stirred at rt for 15 min. Then, a solution of (*R*)-oxiran-2-ylmethyl 4-methylbenzenesulfonate (**11**, 140 mg, 0.61 mmol) in 1 mL of anh. DMF was added to the previous suspension and the mixture was stirred at rt overnight. The reaction was quenched by addition of 20 mL of NH_4Cl saturated solution and the resulting aqueous layer was extracted with EtOAc (3×20 mL). The combined organic extracts were washed with 10% v/w $CuSO_4$ (2×20 mL), dried over anh. $MgSO_4$, filtered and evaporated. The crude was purified by column chromatography (12 g silica column equilibrated with 2% Et_3N in hexane and eluted with a gradient of 0-30% EtOAc in hexane) to obtain 86 mg (73% yield) of the expected product **16aa**. Mp = 73 - 74 ºC. IR (ATR-FTIR): ν_{max} = 2987, 2932, 1611, 1513, 1251 cm^{-1}. ^1H NMR (400 MHz, $CDCl_3$) δ: 7.08 – 7.03 (m, 4H), 6.85 – 6.80 (m, 4H), 4.47 (quint, *J* = 6.0 Hz, 1H), 4.21 – 4.15 (m, 2H), 4.04 (dd, *J* = 9.5, 5.4 Hz, 1H), 3.97 – 3.88 (m, 4H), 3.35 (dddd, *J* = 5.8, 4.1, 3.2, 2.6 Hz, 1H), 2.90 (dd, *J* = 5.0, 4.1 Hz, 1H), 2.82 (s, 4H), 2.75 (dd, *J* = 5.0, 2.6 Hz, 1H), 1.46 (bs, 3H), 1.41 (bs, 3H) ppm. ^{13}C NMR (101 MHz, $CDCl_3$) δ: 157.0, 156.9, 134.6, 134.5, 129.6, 129.5, 114.6, 114.5, 109.9, 74.2, 69.0, 69.0, 67.1, 50.4, 44.9, 37.4, 37.4, 27.0, 25.5 ppm. HRMS (ESI): m/z $[M + H]^+$ calculated for $C_{23}H_{29}O_5$ 385.2010; found 385.2020.

(*R*)-3-(4-(4-((*S*)-3-chloro-2-hydroxypropoxy)phenethyl)phenoxy)propane-1,2-diol (4aa). *Procedure A.* The starting material ((*R*)-3-(4-(4-((*S*)-3-chloro-2-hydroxypropoxy)phenethyl)phenoxy)propane-1,2-diol, (**16aa**, 75 mg, 0.19 mmol) was dissolved in 5 mL of ACN. Then, $CeCl_3 \cdot 7H_2O$ (182 mg, 0.49 mmol) was added and the mixture was refluxed at 100 ºC overnight. The reaction mixture was allowed to cool down to rt and evaporated. The paste was dissolved in MeOH, filtered through a Celite® pad and evaporated. The crude was purified by column chromatography (4 g silica column, eluted with a gradient of 50-100% EtOAc in hexane). Compound-containing fractions were evaporated to obtain the expected product **4aa** (36 mg, 48% yield) as a white solid.

Procedure B. A solution of ((*R*)-3-(4-((4-((*S*)-3-chloro-2-hydroxypropoxy)phenyl)ethynyl)phenoxy)propane-1,2-diol (**1aa**, 38 mg, 0.10 mmol) in 2.5 mL of MeOH was prepared and 5.4 mg of Pd 10% over C were added to this solution. The mixture was purged with N_2 and with H_2 and stirred at r.t. overnight. The resulting suspension was then filtered through a Celite® pad, washed with MeOH and evaporated to obtain 40 mg (100%) of the expected product. Mp = 133 - 134 ºC. $[\alpha]_D$ (*c* 0.37, MeOH) = +9.28. IR (ATR-FTIR): ν_{max} = 3338, 3029, 2921, 2844, 1608, 1510, 1240, 1036, 824, 810 cm^{-1}. ^1H NMR (400 MHz, $CDCl_3$) δ: 7.05 – 7.01 (m, 4H), 6.81 – 6.71 (m, 4H), 4.15 (quint, *J* = 5.2 Hz, 1H), 4.07 – 4.01 (m, 3H), 3.98 – 3.96 (m, 2H), 3.85 – 3.79 (m, 1H), 3.76 – 3.65 (m, 3H), 2.79 (s, 4H) ppm. ^{13}C NMR (101 MHz, $CDCl_3$) δ: 156.8, 156.3, 134.7, 134.5, 129.6, 129.5, 114.5, 114.5, 70.4, 69.8, 69.4, 68.8, 63.5, 46.0, 37.3 ppm. HRMS (ESI): m/z $[M + H]^+$ calculated for $C_{20}H_{26}ClO_5$ 381.1463; found 381.1462.

Synthesis of compound (1ab)

(*S*)-4-((4-((2,2-dimethyl-1,3-dioxolan-4-yl)methoxy)phenyl)ethynyl)-2-methylphenol (5ab). Following the procedure described for **5aa**, starting from 475 mg of **6a** and 2-methyl-4-iodophenol, compound **5ab** was obtained in 88% yield after 18 h reaction. Mp = 124 - 125 ºC. $[\alpha]_D$ (*c* 0.97, $CHCl_3$) = +8.97. IR (ATR-FTIR): ν_{max} = 3385, 2969, 2926, 2891, 1607, 1512, 1049, 835, 823 cm^{-1}. ^1H NMR (400 MHz, $CDCl_3$) δ: 7.45 – 7.38 (m, 2H), 7.30 (dd, *J* = 2.1, 0.9 Hz, 1H), 7.24 (ddd, *J* = 8.2, 2.1, 0.6 Hz, 1H), 6.89 – 6.83 (m, 2H), 6.72 (d, *J* = 8.2 Hz, 1H), 5.01 (s, 1H), 4.49 (quint, *J* = 5.8 Hz, 1H), 4.17 (dd, *J* = 8.5, 6.4 Hz, 1H), 4.06 (dd, *J* = 9.5, 5.4 Hz, 1H), 3.96 (dd, *J* = 9.5, 5.8 Hz, 1H), 3.91 (dd, *J* = 8.5, 5.8 Hz, 1H), 2.24 (s, 3H), 1.47 (bs, 3H), 1.41 (bs, 3H) ppm. ^{13}C NMR (101 MHz, $CDCl_3$) δ: 158.4, 154.1, 134.4, 133.0, 130.7, 124.2, 116.5, 115.8, 115.1, 114.7, 110.0, 88.4, 87.6, 74.1, 68.9, 66.9, 26.9, 25.5, 15.7 ppm. HRMS (ESI): m/z $[M + H]^+$ calculated for $C_{21}H_{23}O_4$ 339.1591; found 339.1587.

(*R*)-3-(4-((4-((*S*)-3-chloro-2-hydroxypropoxy)-3-methylphenyl)ethynyl)phenoxy)propane-1,2-diol (1ab). Following the procedures

described for the preparation of **1aa** but starting from 200 mg of **5ab** (two steps), compound **1ab** was obtained in 83% yield. Mp = 89 - 90 ºC. $[\alpha]_D$ (*c* 0.41, MeOH) = −1.53. IR (ATR-FTIR): v_{max} = 3274, 2921, 2853, 1606, 1510, 1456, 1238, 1109, 1037, 833, 813 cm^1. ^1H NMR (400 MHz, CD_3OD) δ: 7.41 – 7.38 (m, 2H), 7.29 – 7.25 (m, 2H), 6.95 – 6.91 (m, 2H), 6.87 (d, *J* = 8.4 Hz, 1H), 4.17 (quint, *J* = 5.2 Hz, 1H), 4.09 – 4.04 (m, 3H), 4.00 – 3.94 (m, 2H), 3.80 (dd, *J* = 11.3, 4.9 Hz, 1H), 3.73 – 3.63 (m, 3H), 2.21 (s, 3H) ppm. ^{13}C NMR (101 MHz, CD_3OD) δ: 160.1, 156.0, 134.5, 133.7, 131.4, 128.1, 117.2, 117.0, 115.6, 112.1, 89.0, 88.5, 71.6, 70.9, 70.3, 70.1, 64.0, 46.8, 16.2 ppm. HRMS (ESI): m/z $[M + H]^+$ calculated for $C_{21}H_{24}ClO_5$ 391.1307, found 391.1306.

<u>Synthesis of compound (2ab)</u>

(*R*)-3-(4-((*Z*)-4-((*S*)-3-chloro-2-hydroxypropoxy)-3-methylstyryl)phenoxy)propane-1,2-diol (2ab). In a flame dried Schlenk flask, a solution of the starting alkyne (*R*)-3-(4-((4-((*S*)-3-chloro-2-hydroxypropoxy)-3-methylphenyl)ethynyl)phenoxy)propane-1,2-diol (**1ab**, 75 mg, 0.19 mmol), $Pd_2(dba)_3$ (9 mg, 0.01 mmol), dppb (16 mg, 0.04 mmol) and Et_3N (67 µL, 0.48 mmol) was prepared in 0.2 mL of anhydrous dioxane under N_2 atmosphere. After stirring the solution at room temperature during 15 minutes, 15 µL (0.39 mmol) of formic acid were added. The resulting mixture was heated at 80 ºC and stirred 18 h. Then the solvent was removed under vacuum and the crude was purified by column chromatography (25 g silica/ $AgNO_3$ column, eluted with DCM/MeOH 100:0 to 90:10) to yield 24 mg (32%) of **2ab** as a colourless oil. $[\alpha]_D$ (*c* 0.39, DMSO) = –2.33. IR (ATR-FTIR): v_{max} = 3376, 2925, 2875, 1603, 1510, 1246, 1037, 735 cm^{-1}. ^1H NMR (400 MHz, CD_3OD) δ: 7.17 – 7.13 (m, 2H), 7.03 – 7.00 (m, 2H), 6.82 – 6.78 (m, 2H), 6.74 (d, *J* = 9.0 Hz, 1H), 6.42 (s, 2H), 4.14 (quint, *J* = 5.1 Hz, 1H), 4.04 – 4.00 (m, 3H), 3.98 – 3.92 (m, 2H), 3.79 (dd, *J* = 11.3, 4.8 Hz, 1H), 3.72 – 3.64 (m, 3H), 2.11 (s, 3H) ppm. ^{13}C NMR (101 MHz, CD_3OD) δ: 159.4, 157.2, 132.3, 131.6, 131.4, 131.1, 129.5, 129.2, 128.5, 127.5, 115.2, 111.9, 71.8, 71.0, 70.3, 70.1, 64.2, 47.0, 16.3 ppm. HRMS (ESI): m/z $[M + H]^+$ calculated for $C_{21}H_{26}ClO_5$: 393.1463; found: 393.1457.

<u>Synthesis of compound (3ab)</u>

(*R*)-3-(4-((*E*)-4-((*S*)-3-chloro-2-hydroxypropoxy)-2-methylstyryl)phenoxy)propane-1,2-diol (3ab). Compound **2ab** or a mixture of **2ab** and **3ab** (*Z* and *E* isomers) (38 mg, 0.10 mmol) was dissolved in 0.2 mL of dioxane and treated with aqueous formic acid (30 µL, 25% in water, 0.19 mmol) at 80 ºC for 5 h. Then the solvent was evaporated and the resulting crude was chromatographed (0-100% EtOAc in hexane, 4 g column) to yield 28 mg (73%) of **3ab** as a white solid. $[\alpha]_D$ (*c* 1.04, MeOH) = +0.51. IR (ATR-FTIR): v_{max} = 3372, 2924, 2879, 1606, 1509, 1252, 1039, 962, 822 cm^{-1}. ^1H NMR (400 MHz, CD_3OD) δ: 7.44 – 7.41 (m, 2H),

7.32 – 7.31 (m, 1H), 7.29 – 7.26 (m, 1H), 6.94 – 6.91 (m, 4H), 6.86 (d, *J* = 8.4 Hz, 1H), 4.16 (quint, *J* = 5.1 Hz, 1H), 4.09 – 4.04 (m, 3H), 4.00 – 3.95 (m, 2H), 3.81 (dd, *J* = 11.3, 4.8 Hz, 1H), 3.74 – 3.63 (m, 3H), 2.24 (s, 3H) ppm. ^{13}C NMR (101 MHz, CD_3OD) δ: 159.8, 157.6, 132.2, 132.0, 129.4, 128.4, 128.0, 127.4, 127.1, 126.2, 115.8, 112.4, 71.8, 71.1, 70.4, 70.2, 64.2, 47.0, 16.4 ppm. HRMS (ESI): m/z $[M + H]^+$ calculated for $C_{21}H_{26}ClO_5$: 393.1463; found: 393.1454.

Synthesis of compound (4ab)

((*R*)-3-(4-(4-((*S*)-3-chloro-2-hydroxypropoxy)-3-methylphenethyl)phenoxy)propane-1,2-diol (4ab)

A suspension of Pd/C (9.7 mg, 5% mol) and alkyne **1ab** (71 mg, 0.18 mmol) in 5 mL of MeOH was purged with N_2 and H_2. The resulting mixture was vigorously stirred overnight under hydrogen (1 bar). Then the reaction was filtered through a Celite® pad and evaporated *in vacuo* to obtain 71 mg (99%) of the pure product. ^{1}H NMR (400 MHz, CD_3OD) δ: 7.05 – 7.01 (m, 2H), 6.91 – 6.86 (m, 2H), 6.84 – 6.81 (m, 2H), 6.75 (d, *J* = 8.2 Hz, 1H), 4.16 – 4.10 (m, 1H), 4.02 – 3.90 (m, 5H), 3.81 – 3.62 (m, 4H), 2.80 – 2.72 (m, 4H), 2.17 (s, 3H) ppm. ^{13}C NMR (101 MHz, CD_3OD) δ: 158.5, 156.2, 135.5, 135.3, 131.9, 130.4, 127.7, 127.5, 115.3, 112.3, 71.8, 71.1, 70.3, 70.2, 64.2, 47.0, 38.4, 38.4, 16.4 ppm. HMRS (ESI): m/z $[M + H]^+$ calculated for $C_{21}H_{28}ClO_5$: 395.1620; found: 395.1629.

Synthesis of compound (1ac)

(*S*)-4-((4-((2,2-dimethyl-1,3-dioxolan-4-yl)methoxy)phenyl)ethynyl)-2-methoxyphenol (5ac). Following the procedure described for **5aa**, starting from 671 mg of **6a** and 2-methoxy-4-iodophenol, compound **5ac** was obtained after 5 h of reaction in 75% yield. Mp = 124 - 125 ºC. $[\alpha]_D$ (*c* 0.99, $CHCl_3$) = +7.14. IR (ATR-FTIR): ν_{max} = 3355, 2977, 2939, 2913, 2857, 1603, 1517, 1251, 1229, 1014, 825 cm^{-1}. ^{1}H NMR (400 MHz, $CDCl_3$) δ: 7.46 – 7.40 (m, 2H), 7.07 (dd, *J* = 8.2, 1.8 Hz, 1H), 7.01 (d, *J* = 1.8 Hz, 1H), 6.88 (dd, *J* = 8.5, 1.7 Hz, 3H), 5.72 (s, 1H), 4.48 (quint, *J* = 5.9 Hz, 1H), 4.17 (dd, *J* = 8.5, 6.4 Hz, 1H), 4.07 (dd, *J* = 9.5, 5.4 Hz, 1H), 3.96 (dd, *J* = 9.5, 5.9 Hz, 1H), 3.91 (dd, *J* = 8.5, 5.8 Hz, 1H), 3.91 (s, 3H), 3.88 (m, 4H), 1.47 (bs, 3H), 1.41 (bs, 3H) ppm. ^{13}C NMR (101 MHz, $CDCl_3$) δ: 158.5, 146.3, 146.2, 133.0, 125.6, 116.3, 115.2, 114.7, 114.7, 113.8, 110.0, 88.5, 87.5, 74.1, 68.9, 66.9, 56.1, 26.9, 25.5 ppm. HRMS (ESI): m/z $[M + H]^+$ calculated for $C_{21}H_{23}O_5$ 355.1540, found 355.1539.

(*R*)-3-(4-((4-((*S*)-3-chloro-2-hydroxypropoxy)-3-methoxyphenyl)ethynyl)phenoxy)propane-1,2-diol (1ac). Following the procedure described for the preparation of **1aa** but starting from 100 mg of **5ac** (two steps), compound

1ac was obtained in 79% yield. Mp = 134 - 135 ºC. [α]$_D$ (*c* 0.87, MeOH) = −2.99. IR (ATR-FTIR): $ν_{max}$ = 3317, 2922, 2857, 1606, 1517, 1246, 1221, 1026, 833 cm^{-1}. ^{1}H NMR (400 MHz, CD_3OD) δ: 7.43 – 7.40 (m, 2H), 7.08 – 7.06 (m, 2H), 6.97 – 6.93 (m, 3H), 4.17 – 4.06 (m, 4H), 4.01 – 3.95 (m, 2H), 3.86 (s, 3H), 3.80 (dd, *J* = 11.3, 4.8 Hz, 1H), 3.72 – 3.62 (m, 3H) ppm. ^{13}C NMR (101 MHz, CD_3OD) δ: 160.4, 150.7, 149.9, 133.8, 125.8, 118.2, 117.1, 116.1, 115.8, 115.0, 88.8, 88.8, 71.7, 71.4, 71.0, 70.4, 64.1, 56.6, 46.8 ppm. HRMS (ESI): m/z [M + H]$^+$ calculated for $C_{21}H_{24}ClO_6$ 407.1256; found 407.1256.

Synthesis of compound (1ad)

(*S*)-4-((4-((2,2-dimethyl-1,3-dioxolan-4-yl)methoxy)phenyl)ethynyl)-2-fluorophenol (5ad). Following the procedure described for **5aa**, starting from 701 mg of **6a** and 2-fluorophenol, compound **5ad** was obtained in 79% yield after 6 h of reaction. Mp = 128 - 129 ºC. [α]$_D$ (*c* 0.99, $CHCl_3$) = +10.52. IR (ATR-FTIR): $ν_{max}$ = 3205, 2990, 2930, 2896, 2870, 1611, 1577, 1519, 1326, 1062, 827 cm^{-1}. ^{1}H NMR (400 MHz, $CDCl_3$) δ: 7.46 – 7.40 (m, 2H), 7.23 (dd, *J* = 11.1, 1.9 Hz, 1H), 7.19 (ddd, *J* = 8.4, 2.0, 1.1 Hz, 1H), 6.99 – 6.92 (m, 1H), 6.91 – 6.85 (m, 2H), 5.41 (d, *J* = 3.9 Hz, 1H), 4.49 (quint, *J* = 5.8 Hz, 1H), 4.18 (dd, *J* = 8.5, 6.4 Hz, 1H), 4.07 (dd, *J* = 9.5, 5.4 Hz, 1H), 3.96 (dd, *J* = 9.5, 5.8 Hz, 1H), 3.91 (dd, *J* = 8.5, 5.8 Hz, 1H), 1.47 (bs, 3H), 1.41 (bs, 3H) ppm. ^{13}C NMR (101 MHz, $CDCl_3$) δ: 158.7, 150.6 (d, J_F = 238.3 Hz), 144.1 (d, J_F = 14.3 Hz), 133.1, 128.7 (d, J_F = 3.1 Hz), 118.7 (d, J_F = 19.3 Hz), 117.5 (d, J_F = 2.4 Hz), 116.2 (d, J_F = 8.2 Hz), 115.9, 114.7, 110.1, 88.4, 87.2 (d, J_F = 2.9 Hz), 74.1, 68.9, 66.9, 26.9, 25.5 ppm. HRMS (ESI): m/z [M + H]$^+$ calculated for $C_{20}H_{20}FO_4$ 343.1340; found 343.1347.

(*R*)-3-(4-((4-((*S*)-3-chloro-2-hydroxypropoxy)-3-fluorophenyl)ethynyl)phenoxy)propane-1,2-diol (1ad). Following the procedure described for the preparation of **1aa** but starting from **5ad** (two steps), compound **1ad** was obtained in 39% yield. Mp = 116 - 118 ºC. [α]$_D$ (*c* 0.53, MeOH) = −4.72. IR (ATR-FTIR): $ν_{max}$ = 3376, 3243, 2917, 2848, 2509, 2410, 1519, 1321, 1296, 1270, 1244, 1014 cm^{-1}. ^{1}H NMR (400 MHz, CD_3OD) δ: 7.45 – 7.39 (m, 2H), 7.25 – 7.21 (m, 2H), 7.10 (t, *J* = 8.7 Hz, 1H), 6.97 – 6.94 (m, 2H), 4.19 – 4.13 (m, 3H), 4.11 – 4.06 (m, 1H), 4.01 – 3.95 (m, 2H), 3.78 (dd, *J* = 11.2, 4.7 Hz, 1H), 3.72 – 3.63 (m, 3H) ppm. ^{13}C NMR (101 MHz, CD_3OD) δ: 160.6, 153.4 (d, J_F = 246.2 Hz), 148.3 (d, J_F = 11.0 Hz), 133.9, 129.1 (d, J_F = 3.5 Hz), 119.8 (d, J_F = 19.9 Hz), 118.2 (d, J_F = 8.5 Hz), 116.7, 116.3 (d, J_F = 2.3 Hz), 115.8, 89.7, 87.5 (d, J_F = 2.7 Hz), 71.7, 71.4, 70.8, 70.4, 64.1, 46.6 ppm. HRMS (ESI): m/z [M + H]$^+$ calculated for $C_{20}H_{21}ClFO_5$ 395.1056; found 395.1057.

Synthesis of compound (1ae)

(*S*)-5-((4-((2,2-dimethyl-1,3-dioxolan-4-yl)methoxy)phenyl)ethynyl)-[1,1'-biphenyl]-2-ol (5ae). Following the procedure described for **5aa,** starting from 421 mg of **6a** and 5-iodo-[1,1'-biphenyl]-2-ol, compound **5ae** was obtained in 95% yield after 18 h of reaction. Mp = 101 - 102 ºC. $[\alpha]_D$ (*c* 1.02, $CHCl_3$) = +5.98. IR (ATR-FTIR): ν_{max} = 3312, 2990, 2926, 2866, 1607, 1508, 1272, 1224, 1056, 1036, 822, 698 cm^{-1}. ^1H NMR (400 MHz, $CDCl_3$) δ: 7.54 – 7.39 (m, 9H), 6.97 – 6.94 (m, 1H), 6.90 – 6.85 (m, 2H), 5.34 (s, 1H), 4.48 (quint, *J* = 5.8 Hz, 1H), 4.17 (dd, *J* = 8.5, 6.4 Hz, 1H), 4.07 (dd, *J* = 9.6, 5.4 Hz, 1H), 3.96 (dd, *J* = 9.5, 5.9 Hz, 1H), 3.90 (dd, *J* = 8.5, 5.8 Hz, 1H), 1.47 (bs, 3H), 1.41 (bs, 3H) ppm. ^{13}C NMR (101 MHz, $CDCl_3$) δ: 152.8, 152.8, 136.6, 133.7, 132.9, 132.3, 129.2, 129.1, 128.6, 128.5, 128.0, 116.3, 116.2, 115.9, 114.6, 109.9, 88.1, 88.0, 74.0, 68.7, 66.7, 26.8, 25.4 ppm. HRMS (ESI): m/z [M + H]$^+$ calculated for $C_{26}H_{25}O_4$ 401.1747; found 401.1743.

(*R*)-3-(4-((6-((*S*)-3-chloro-2-hydroxypropoxy)-[1,1'-biphenyl]-3-yl)ethynyl)phenoxy)propane-1,2-diol (1ae) Following the procedure described for the preparation of **1aa** but starting from **5ae** (two steps), compound **1ae** was obtained in 85% yield. Mp = 89 - 91 ºC. $[\alpha]_D$ (*c* 0.47, MeOH) = −0.77. IR (ATR-FTIR): ν_{max} = 3321, 2922, 2853, 1616, 1509, 1238, 1031, 1001, 768, 699 cm^{-1}. ^1H NMR (400 MHz, $CDCl_3$) δ: 7.51 – 7.48 (m, 2H), 7.44 – 7.36 (m, 6H), 7.33 – 7.28 (m, 1H), 7.03 (d, *J* = 8.5 Hz, 1H), 6.94 – 6.91 (m, 2H), 4.10 – 4.02 (m, 4H), 4.00 – 3.95 (m, 2H), 3.71 – 3.58 (m, 3H), 3.54 – 3.49 (m, 1H) ppm. ^{13}C NMR (101 MHz, $CDCl_3$) δ: 160.1, 156.6, 138.9, 134. 6, 133.8, 132.9, 132.6, 130.5, 128.9, 128.2, 117.7, 117.0, 115.6, 113.8, 89.1, 88.6, 71.6, 70.5, 70.3, 70.2, 64.0, 46.8 ppm. HRMS (ESI): m/z [M + H]$^+$ calculated for $C_{26}H_{26}ClO_5$ 453.1463; found 453.1477.

Synthesis of compound (1af)

(*S*)-2-(*tert*-butyl)-4-((4-((2,2-dimethyl-1,3-dioxolan-4-yl)methoxy)phenyl)ethynyl)phenol (5af). Following the procedure described for **5aa**, starting from 700 mg of **6a** and 2-(*tert*-butyl)-4-iodophenol, compound **5af** was obtained in 71% yield after 18 h of reaction. Mp = 111 - 113 ºC. $[\alpha]_D$ (*c* 0.98, $CHCl_3$) = +6.26. IR (ATR-FTIR): ν_{max} = 3373, 2960, 2922, 2887, 2866, 1606, 1510, 1404, 1368, 1243, 1202, 1053, 828 cm^{-1}. ^1H NMR (400 MHz, $CDCl_3$) δ: 7.48 – 7.40 (m, 3H), 7.23 (dd, *J* = 8.1, 2.0 Hz, 1H), 6.90 – 6.84 (m, 2H), 6.63 (d, *J* = 8.2 Hz, 1H), 5.14 (s, 1H), 4.49 (quint, *J* = 5.8 Hz, 1H), 4.18 (dd, *J* = 8.5, 6.4 Hz, 1H), 4.07 (dd, *J* = 9.5, 5.4 Hz, 1H), 3.96 (dd, *J* = 9.6, 5.9 Hz, 1H), 3.91 (dd, *J* = 8.5, 5.8 Hz, 1H), 1.47 (s, 3H), 1.41 (s, 12H) ppm. ^{13}C NMR (101 MHz, $CDCl_3$) δ: 158.4, 154.5, 136.5, 133.0, 130.9, 130.5, 116.8, 116.5, 115.5, 114.7, 110.0, 88.8, 87.4, 74.1, 68.9, 66.9, 34.8, 29.6, 26.9, 25.5 ppm. HRMS (ESI): m/z [M + H]$^+$ calculated for $C_{24}H_{29}O_4$ 381.2060; found 381.2060.

(*R*)-3-(4-((3-(*tert*-butyl)-4-((*S*)-3-chloro-2-hydroxypropoxy)phenyl)ethynyl)phenoxy)propane-1,2-diol (1af) Following the

procedure described for the preparation of **1aa** but starting from 95 mg of **5af** (two steps), compound **1af** was obtained in 59% yield**.** Mp = 116 - 118 °C. $[\alpha]_D$ (*c* 0.43, MeOH) = −3.14. IR (ATR-FTIR): ν_{max} = 3327, 2948, 2857, 1606, 1511, 1241, 1228, 1036, 811 cm^{-1}. ^1H NMR (400 MHz, $CDCl_3$) δ: 7.43 – 7.29 (m, 3H), 7.31 (dd, *J* = 8.4, 2.1 Hz, 1H), 6.95 – 6.91 (m, 3H), 4.23 (quint, *J* = 5.2 Hz, 1H), 4.15 – 4.05 (m, 3H), 4.01 – 3.96 (m, 2H), 3.81 (dd, *J* = 11.3, 4.9 Hz, 1H), 3.74 (dd, *J* = 11.3, 5.5 Hz, 1H), 3.69 – 3.63 (m, 2H), 1.40 (s, 9H) ppm. ^{13}C NMR (101 MHz, $CDCl_3$) δ: 160.1, 158.6, 139.4, 133.7, 131.5, 130.9, 117.3, 116.9, 115.7, 113.3, 89.3, 88.4, 71.6, 70.9, 70.3, 70.1, 64.1, 47.2, 35.7, 30.3 ppm. HRMS (ESI): m/z $[M + H]^+$ calculated for $C_{24}H_{30}ClO_5$ 433.1776; found 433.1778.

Synthesis of compound (1ba)

(*S*)-4-((4-((2,2-dimethyl-1,3-dioxolan-4-yl)methoxy)-3-methylphenyl)ethynyl)phenol (5ba). Following the procedure described for **5aa**, starting from 457 mg of **6b** and 4-iodophenol**,** compound **5ba** was obtained in 87% yield after 16 h of reaction. Mp = 134 - 136. $[\alpha]_D$ (*c* 0.92, $CHCl_3$) = +19.28. IR (Film): ν_{max} = 3298, 2089, 1608, 1513, 1238, 1041, 835, 805 cm^{-1}. ^1H NMR (400 MHz, $CDCl_3$) δ: 7.39 – 7.34 (m, 2H), 7.35 – 7.28 (m, 2H), 6.80 – 6.77 (m, 2H), 6.77 – 6.73 (m, 1H), 6.03 (s, 1H), 4.51 (qd, *J* = 6.0, 4.7 Hz, 1H), 4.19 (dd, *J* = 8.5, 6.4 Hz, 1H), 4.08 (dd, *J* = 9.7, 4.7 Hz, 1H), 4.02 – 3.94 (m, 2H), 2.20 (s, 2H), 1.49 (d, *J* = 0.8 Hz, 3H), 1.43 (d, *J* = 0.8 Hz, 3H) ppm. ^{13}C NMR (101 MHz, $CDCl_3$) δ: 156.7, 155.9, 133.9, 133.2, 130.5, 127.1, 115.9, 115.8, 115.6, 111.0, 110.0, 88.1, 87.9, 74.2, 68.5, 66.8, 26.8, 25.5, 16.2 ppm. HRMS (ESI): m/z $[M + H]^+$ calculated for $C_{21}H_{22}O_4$ 338.1513; found: 338.1506.

(*R*)-3-(4-((4-((*S*)-3-chloro-2-hydroxypropoxy)phenyl)ethynyl)-2-methylphenoxy)propane-1,2-diol (1ba). Following the procedure described for the preparation of **1aa** but starting from 116 mg of **5ba** (two steps), compound **1ba** was obtained in 64% yield. Mp = 135 - 137 °C. $[\alpha]_D$ (*c* 0.93, MeOH) = +0.54. IR (Film): ν_{max} = 3315, 2922, 2873, 2199, 1605, 1508, 1239, 1032, 808 cm^{-1}. ^1H NMR (400 MHz, $CDCl_3$) δ: 7.42 – 7.38 (m, 2H), 7.29 – 7.25 (m, 2H), 6.96 – 6.92 (m, 2H), 6.88 (d, *J* = 8.4 Hz, 1H), 4.16 – 3.97 (m, 6H), 3.79 – 3.65 (m, 4H), 2.22 (s, 3H) ppm. ^{13}C NMR (101 MHz, $CDCl_3$) δ: 159.9, 158.4, 134.5, 133.8, 131.4, 128.2, 117.6, 116.7, 115.8, 112.1, 89.1, 88.3, 71.8, 70.9, 70.3, 70.2, 64.2, 46.7, 16.2 ppm. HRMS (ESI): m/z $[M + H]^+$ calculated for $C_{21}H_{24}ClO_5$ 391.1307; found: 391.1306.

Synthesis of compound (1bb)

(*S*)-4-((4-iodo-3-methylphenoxy)methyl)-2,2-dimethyl-1,3-dioxolane (8b). A mixture of 4-iodo-2-methylphenol (1.05 g, 4.47 mmol) and Cs_2CO_3 (2.92 g, 8.95 mmol) in 10 mL of anh.

DMF was heated up to 80 ºC under N_2 atm. for 15 min. Then, a solution of (*R*)-(2,2-dimethyl-1,3-dioxolan-4-yl)methyl 4-methylbenzenesulfonate (**10**, 1.18 g, 8.95 mmol) in dry DMF (5 mL) was added dropwise. The reaction was stirred overnight at 80 ºC and quenched by addition of 50 mL of a saturated $KHCO_3$ solution. The resulting aqueous layer was extracted with EtOAc (3×50 mL). The extracted organic layers were concentrated and washed with 10% v/w $CuSO_4$ (2×20 mL). The organic layer was dried over anh. $MgSO_4$, filtered and evaporated. The resulting crude was purified by column chromatography (25 g column, eluted with a gradient of 0-100% EtOAc in hexane) to obtain 1.36 g (87% yield) of the desired product **8b** as a yellow oil. $[\alpha]_D$ (*c* 1.28, $CHCl_3$) = +16.57. IR (Film) ν_{max} = 2985, 2933, 2980, 1588, 1490, 1246, 1055, 842 cm^{-1}. ^1H NMR (400 MHz, $CDCl_3$) δ: 7.45 – 7.39 (m, 2H), 6.60 – 6.56 (m, 1H), 4.46 (qd, *J* = 6.1, 4.7 Hz, 1H), 4.16 (dd, *J* = 8.4, 6.3 Hz, 1H), 4.03 (dd, *J* = 9.6, 4.7 Hz, 1H), 3.97 – 3.89 (m, 2H), 2.17 (s, 2H), 1.46 (d, *J* = 0.7 Hz, 3H), 1.40 (d, *J* = 0.7 Hz, 3H) ppm. ^{13}C NMR (101 MHz, $CDCl_3$): δ: 156.7, 139.3, 135.6, 129.8, 113.4, 109.80, 83.3, 74.1, 68.7, 66.9, 26.9, 25.5, 16.0 ppm. HRMS (ESI): m/z $[M + H]^+$ calculated for $C_{13}H_{18}IO_3$ 349.0295; found: 349.0297.

(*S*)-((4-((2,2-dimethyl-1,3-dioxolan-4-yl)methoxy)-3-methylphenyl)ethynyl)trimethylsilane (9b). A suspension of the starting material ((*S*)-4-((4-iodo-3-methylphenoxy)methyl)-2,2-dimethyl-1,3-dioxolane (**8b**, 357 mg, 1.02 mmol), CuI (2.0 mg, 0.01 mmol) and $Pd(Ph_3P)_2Cl_2$ (7.2 mg, 0.01 mmol) was prepared in 3 mL of anh. THF. Then, trimethylsilylacetylene (0.22 mL, 1.54 mmol) and 3 mL of Et_3N were added and the resulting mixture was stirred for 2.5 h. The solution was filtered through a Celite® pad and concentrated under vacuum. The resulting crude was purified by column chromatography (25 g column, eluted with a gradient of 0-100% EtOAc in hexane) to afford 324 mg (99% yield) of **9b** as a yellow oil. $[\alpha]_D$ (*c* 1.05, $CHCl_3$) = +19.68. IR (Film): ν_{max} = 2986, 2958, 2897, 2149, 1501, 1229, 843 cm^{-1}. ^1H NMR (400 MHz, $CDCl_3$) δ: 7.29 – 7.25 (m, 2H), 6.74 – 6.70 (m, 1H), 4.47 (qd, *J* = 6.1, 4.6 Hz, 1H), 4.16 (dd, *J* = 8.4, 6.3 Hz, 1H), 4.07 (dd, *J* = 9.6, 4.6 Hz, 1H), 3.98 – 3.92 (m, 2H), 2.18 (s, 2H), 1.46 (d, *J* = 0.7 Hz, 3H), 1.40 (d, *J* = 0.7 Hz, 3H), 0.23 (s, 9H) ppm. ^{13}C NMR (101 MHz, $CDCl_3$) δ: 157.1, 134.5, 131.1, 127.0, 115.4, 110.8, 109.8, 105.5, 92.4, 74.1, 68.6, 67.0, 26.9, 25.6, 16.1, 0.2 ppm. HRMS (ESI): m/z $[M + H]^+$ calculated for $C_{18}H_{27}O_3Si$ 319.1724; found: 319.1718.

(*S*)-4-((4-ethynyl-2-methylphenoxy)methyl)-2,2-dimethyl-1,3-dioxolane (6b). To a solution of (*S*)-((4-((2,2-dimethyl-1,3-dioxolan-4-yl)methoxy)-3-methylphenyl)ethynyl)trimethylsilane (**9b**, 1.18 g, 3.69 mmol) in 10 mL of MeOH, 611 mg (4.43 mmol) of K_2CO_3 were added. The resulting suspension was stirred for 1.5 h, then diluted with H_2O (30 mL) and extracted with DCM (3x30 mL). The organic extracts were dried over anh. $MgSO_4$ and concentrated in vacuo to obtain **6b** (909 mg, 100% yield). $[\alpha]_D$ (c 0.97, $CHCl_3$) = +18.96. IR (ATR-FTIR): ν_{max} = 3289, 2985, 2930, 2880, 1603, 1501, 1371,

1253, 1213, 1127, 1052, 842, 810 cm^{-1}. ^{1}H NMR (400 MHz, $CDCl_3$) δ: 7.32 – 7.27 (m, 2H), 6.75 (d, *J* = 8.3 Hz, 1H), 4.48 (qd, *J* = 6.1, 4.6 Hz, 1H), 4.17 (dd, *J* = 8.4, 6.4 Hz, 1H), 4.08 (dd, *J* = 9.6, 4.6 Hz, 1H), 3.96 (ddd, *J* = 7.1, 6.0, 1.8 Hz, 2H), 2.97 (s, 1H), 2.19 (s, 3H), 1.46 (s, 3H), 1.41 (s, 3H) ppm. ^{13}C NMR (101 MHz, $CDCl_3$) δ: 157.2, 134.5, 131.2, 127.1, 114.3, 110.9, 109.8, 83.9, 75.8, 74.1, 68.6, 66.9, 26.8, 25.5, 16.1 ppm. HRMS (ESI): m/z $[M + H]^+$ calculated for $C_{15}H_{19}O_3$ 247.1329; found: 247.1323.

(*S*)-4-((4-((2,2-dimethyl-1,3-dioxolan-4-yl)methoxy)-3-methylphenyl)ethynyl)-3-methylphenol (5bb). Following the procedure described for **5aa**, starting from 243 mg of **6b** and 2-methyl-4-iodophenol, compound **5bb** was obtained in 89% yield after 2.5 h of reaction. Mp = 107 - 109 ºC. $[\alpha]_D$ (*c* 0.76, MeOH) = +23.23. IR (Film): v_{max} = 2985, 2933, 1585, 1486, 1371, 1243, 1055 cm^{-1}. ^{1}H NMR (400 MHz, $CDCl_3$) δ: 7.32 – 7.28 (m, 3H), 7.23 (dd, *J* = 8.3, 2.1 Hz, 1H), 6.79 – 6.75 (m, 1H), 6.72 (d, *J* = 8.2 Hz, 1H), 4.89 (s, 1H), 4.49 (qd, *J* = 6.1, 4.6 Hz, 1H), 4.18 (dd, *J* = 8.4, 6.3 Hz, 1H), 4.09 (dd, *J* = 9.6, 4.6 Hz, 1H), 3.98 (dd, *J* = 6.0, 1.3 Hz, 1H), 3.96 (d, *J* = 5.9 Hz, 1H), 2.24 (s, 3H), 2.21 (s, 3H), 1.47 (s, 3H), 1.41 (s, 3H) ppm. ^{13}C NMR (101 MHz, $CDCl_3$) δ: 156.7, 154.0, 134.4, 134.0, 130.7, 130.4, 127.2, 124.1, 115.9, 115.1, 111.0, 109.9, 88.0, 87.9, 74.2, 69.0, 67.0, 26.9, 25.6, 16.2, 15.7 ppm. HRMS (ESI): m/z $[M + H]^+$ calculated for $C_{22}H_{25}O_4$ 353.1747; found: 353.1748.

(*R*)-3-(4-((4-((*S*)-3-chloro-2-hydroxypropoxy)-3-methylphenyl)ethynyl)-2-methylphenoxy) propane-1,2-diol (1bb). Following the procedure described for the preparation of **1aa** but starting from 65 mg of **5bb** (two steps), compound **1bb** was obtained in 81% yield**.** $[\alpha]_D$ (*c* 1.02, MeOH) = +1.82. ^{1}H NMR (400 MHz, CD_3OD) δ: 7.29 – 7.24 (m, 4H), 6.88 (d, *J* = 8.3 Hz, 2H), 4.20 – 4.15 (m, 1H), 4.10 – 4.05 (m, 3H), 4.03 – 3.97 (m, 2H), 3.80 (dd, *J* = 11.2, 4.9 Hz, 1H), 3.76 – 3.65 (m, 3H), 2.23 (s, 6H) ppm. ^{13}C NMR (101 MHz, CD_3OD) δ: 158.4, 158.0, 134.5, 134.5, 131.4, 128.2, 117.2, 116.9, 112.2, 112.1, 88.8, 88.6, 71.8, 71.0, 70.3, 70.2, 64.2, 46.9, 16.2, 16.2 ppm. HRMS (ESI): m/z $[M + H]^+$ calculated for $C_{22}H_{26}ClO_5$ 405.1463; found: 405.1463.

Synthesis of compound (2ba)

(*S*)-2,2-dimethyl-4-((2-methyl-4-((*Z*)-4-(((*R*)-oxiran-2-yl)methoxy)styryl)phenoxy)methyl)-1,3-dioxolane (13ba). To a solution of (*S*)-2,2-dimethyl-4-((2-methyl-4-((4-(((R)-oxiran-2-yl)methoxy)phenyl)ethynyl)phenoxy)methyl)-1,3-dioxolane (**12ba**, 77 mg, 0.19 mmol) in 6 mL of toluene was added quinoline (23 µL, 0.19 mmol), Pd/$BaSO_4$ (29 mg, 0.01 mmol) and 6 mL of hexane. The suspension was put under H_2 (balloon). The stirring was kept for 45 minutes and after that time, the suspension was filtered through a Celite® pad and the solvent was removed under vacuum. The resulting crude was chromatographed (0-20% EtOAc in hexane) to obtain **13ba** (54 mg, 70% aprox.)

unpurified with a 10% of the totally reduced product **16ba**. ^{1}H NMR (400 MHz, $CDCl_3$) δ: 7.21 – 7.18 (m, 2H), 7.05 – 7.02 (m, 2H), 6.79 – 6.76 (m, 2H), 6.67 (d, *J* = 8.1 Hz, 1H), 6.42 (s, 2H), 4.50 – 4.44 (m, 1H), 4.22 – 4.15 (m, 2H), 4.06 (dd, *J* = 9.6, 4.6 Hz, 1H), 3.98 – 3.92 (m, 3H), 3.35 (ddt, *J* = 5.7, 4.1, 2.9 Hz, 1H), 2.90 (dd, *J* = 5.0, 4.1 Hz, 1H), 2.75 (dd, *J* = 4.9, 2.7 Hz, 1H), 2.13 (s, 3H), 1.47 (s, 3H), 1.41 (s, 3H) ppm. ^{13}C NMR (101 MHz, $CDCl_3$) δ: 157.5, 155.8, 131.5 130.8, 130.2, 130.1, 128.8, 128.3, 127.4, 126.6, 114.4, 110.9, 109.7, 74.2, 68.8, 68.6, 67.0, 50.3, 44.9, 26.9, 25.6, 16.3 ppm.

(*R*)-3-(4-((*Z*)-4-((*S*)-3-chloro-2-hydroxypropoxy)styryl)-2-methylphenoxy)propane-1,2-diol (2ba). To a solution of (*S*)-2,2-dimethyl-4-((2-methyl-4-((*Z*)-4-(((*R*)-oxiran-2-yl)methoxy)styryl)phenoxy)methyl)-1,3-dioxolane (**13ba,** 52 mg 0.13 mmol)) in 3 mL of ACN was added $CeCl_3·7H_2O$ (123 mg, 0.33 mmol). The resulting suspension was stirred at 100 °C overnight and then filtered through a Celite® pad. After concentrating the crude, it was purified by column chromatography (4 g column, equilibrated with 2% Et_3N in DCM and eluted with a gradient of 0-20% MeOH in DCM) to obtain 25 mg (49%) of the expected product impurified with 10% of the totally reduced product. ^{1}H NMR (400 MHz, $CDCl_3$) δ: 7.16 – 7.13 (m, 2H), 7.00 – 6.98 (m, 2H), 6.75 – 6.70 (m, 2H), 6.63 (d, *J* = 8.1 Hz, 1H), 6.38 (s, 2H), 4.14 (quint, *J* = 5.2 Hz, 1H), 4.05 – 3.93 (m, 5H), 3.78 – 3.63 (m, 4H), 2.09 (s, 3H) ppm. ^{13}C NMR (101 MHz, $CDCl_3$) δ: 157.3, 155.7, 131.4, 130.7, 130.2, 130.0, 128.8, 128.2, 127.4, 126.4, 114.2, 110.8, 70.5, 69.7, 69.0, 68.7, 63.7, 45.9, 16.1 ppm.

Synthesis of compound (4ba)

(*S*)-4-(4-((2,2-dimethyl-1,3-dioxolan-4-yl)methoxy)-3-methylphenethyl)phenol (15ba) A suspension of Pd/C (50%, 6.7 mg, 5% weight) and the starting alkyne (((*S*)-4-((4-((2,2-dimethyl-1,3-dioxolan-4-yl)methoxy)-3-methylphenyl)ethynyl)phenol, (**5ba**, 67 mg, 0.20 mmol) was prepared and purged with N_2 and H_2. The resulting mixture was vigorously stirred overnight. Then the reaction was filtered through a Celite® pad and evaporated to obtain 68 mg (100%) of the pure product **15ba** as a colorless oil. $[\alpha]_D$ (*c* 0.83, $CDCl_3$) = +18.68. IR (Film): ν_{max} = 3360, 2931, 2076, 1735, 1610, 1512, 1456, 1223, 1047, 830, 810 cm^{-1}. ^{1}H NMR (400 MHz, $CDCl_3$) δ: 7.05 – 7.01 (m, 2H), 6.96 – 6.90 (m, 2H), 6.76 – 6.71 (m, 2H), 4.67 (s, 1H), 4.50 – 4.44 (m, 1H), 4.17 (dd, *J* = 8.4, 6.3 Hz, 1H), 4.07 (dd, *J* = 9.6, 4.6 Hz, 1H), 3.98 – 3.90 (m, 2H), 2.83 – 2.75 (m, 4H), 2.20 (s, 3H), 1.47 (s, 3H), 1.41 (s, 1H) ppm. ^{13}C NMR (101 MHz, $CDCl_3$) δ: 155.0, 153.9, 134.3, 134.2, 131.1, 129.6, 126.8, 126.6, 115.3, 111.3, 109.8, 74.3, 68.8, 67.1, 37.5, 26.9, 25.6, 16.3 ppm. HRMS (ESI): m/z $[M + H]^+$ calculated for $C_{21}H_{27}O_4$: 343.1904; found: 343.1905.

(*S*)-2,2-dimethyl-4-((2-methyl-4-(4-(((R)-oxiran-2-yl)methoxy)phenethyl)phenoxy)methyl)-1,3-dioxolane (16ba). (*S*)-4-(4-((2,2-dimethyl-

1,3-dioxolan-4-yl)methoxy)-3-methylphenethyl)phenol (**15ba**, 52 mg, 0.15 mmol) was dissolved in 0.5 mL of anh. DMF and added, under N_2 atm. via cannula (+ 0.5 mL of anh. DMF to wash), to a suspension of NaH (60% dispersion in oil, 12 mg, 0.31 mmol) in 0.5 mL of anh. DMF. The resulting mixture was stirred for 15 minutes. Then a solution of of (*R*)-oxiran-2-ylmethyl 4-methylbenzenesulfonate (35 mg, 0.15 mmol) in 0.5 mL of anh. DMF (+ 0.5 mL to wash) was added and the mixture was left to stir overnight. The reaction was quenched by adding 20 mL of H_2O. The resulting aqueous layer was extracted with EtOAc (3x20 mL), the combined organic layers were dried over anh. $MgSO_4$ and evaporated and the resulting crude was purified by column chromatography (4 g column, eluted with hexane/EtOAc 100:0 to 0:100). The desired product **16ba** was isolated as a yellow oil (43 mg, 70%). $[\alpha]_D$ (*c* 1.31, $CDCl_3$) = +11.58. IR (Film): ν_{max} = 2986, 2923, 1507, 1241, 1218, 1040, 808 cm^{-1}. ^1H NMR (400 MHz, $CDCl_3$) δ: 7.10 – 7.06 (m, 2H), 6.95 (d, *J* = 2.3 Hz, 1H), 6.92 (dd, *J* = 8.0, 2.4 Hz, 1H), 6.86 – 6.82 (m, 2H), 6.72 (d, *J* = 8.2 Hz, 1H), 4.47 (qd, *J* = 6.2, 4.6 Hz, 1H), 4.21 – 4.15 (m, 2H), 4.07 (dd, *J* = 9.5, 4.6 Hz, 1H), 3.35 (dddd, *J* = 5.8, 4.1, 3.3, 2.7 Hz, 1H), 2.90 (dd, *J* = 5.0, 4.1 Hz, 1H), 2.84 – 2.75 (m, 4H), 2.76 (dd, *J* = 5.0, 2.7 Hz, 1H), 2.20 (s, 3H), 1.47 (s, 3H), 1.41 (s, 3H) ppm. ^{13}C NMR (101 MHz, $CDCl_3$) δ: 156.9, 155.1, 134.8, 134.3, 131.1, 129.5, 126.8, 126.6, 114.6, 111.3, 109.7, 74.3, 69.0, 68.9, 67.1, 50.4, 44.9, 37.5, 37.4, 26.9, 25.6, 16.3 ppm.

(*R*)-3-(4-(4-((S)-3-chloro-2-hydroxypropoxy)phenethyl)-2-methylphenoxy)propane-1,2-diol (4ba). A suspension of $CeCl_3 \cdot 7H_2O$ (106 mg, 0.29 mmol) and the starting material (**16ba**, (*S*)-2,2-dimethyl-4-((2-methyl-4-(4-(((R)-oxiran-2-yl)methoxy)phenethyl)phenoxy)methyl)-1,3-dioxolane (46 mg, 0.11 mmol) in 3 mL of ACN was heated to 100 ºC overnight and then filtered through a Celite® pad. The resulting crude was purified by column chromatography (4 g column, eluted with a mixture of DCM/MeOH 100:0 to 80:20) to obtain the desired product **4ba** (40 mg, 89%) as a white solid. Mp = 107 - 109 ºC. $[\alpha]_D$ (*c* 1.00, MeOH) = +23.23. IR (Film): ν_{max} = 3281, 2934, 1736, 1510, 1245, 1214, 1042, 1031, 821, 801 cm^{-1}. ^1H NMR (400 MHz, $CDCl_3$) δ: 7.05 – 7.02 (m, 2H), 6.90 – 6.88 (m, 2H), 6.84 – 6.80 (m, 2H), 6.75 (d, *J* = 8.1 Hz, 1H), 4.13 – 4.08 (m, 1H), 4.01 – 3.93 (m, 4H), 3.77 – 3.71 (m, 2H), 3.68 – 3.64 (m, 2H), 2.80 – 2.72 (m, 5H), 2.18 (s, 3H) ppm. ^{13}C NMR (101 MHz, $CDCl_3$) δ: 158.3, 156.6, 135.8, 135.1, 131.9, 130.5, 127.7, 127.5, 115.4, 112.1, 72.0, 71.0, 70.4, 70.1, 64.4, 46.8, 38.5, 16.4 ppm. HRMS (ESI): m/z $[M + H]^+$ calculated for $C_{21}H_{28}ClO_5$: 395.1620; found: 395.1617.

<u>Synthesis of compound (1'bb).</u>

(*R*)-1-azido-3-(4-((4-(((*S*)-2,2-dimethyl-1,3-dioxolan-4-yl)methoxy)-3-methylphenyl)ethynyl)-2-methylphenoxy)propan-2-ol (17bb)

A solution of the starting material **12bb** ((*S*)-2,2-dimethyl-4-((2-methyl-4-((3-methyl-4-(((*R*)-oxiran-2-yl)methoxy)phenyl)ethynyl)phenoxy)methyl)-1,3-dioxolane, 46 mg, 0.11 mmol), sodium azide (88 mg, 1.36 mmol), 4-nitrobenzoic acid (22 mg, 0.11 mmol) and 15-crown-5 (25 μL, 0.12 mmol) was prepared under N_2 atm. in 2 mL of dry DMF and heated up to 100 ºC. When no starting material was observed, 2 mL of saturated solution of $NaHCO_3$ were added dropwise and the resulting mixture was diluted with water (15 mL) and extracted with EtOAc (3x15 mL). The combined organic extracts were dried over anh. $MgSO_4$ and concentrated *in vacuo* to obtain 45 mg (87%) of the expected azide **17bb**. Mp = 119 - 120 ºC. IR: v_{max} = (Film): 3417, 2922, 2091, 1737, 1606, 1504, 1240, 846, 808 cm^{-1}. ^{1}H NMR (400 MHz, CD_3OD) δ: 7.33 – 7.30 (m, 4H), 6.78 – 6.76 (m, 2H), 4.49 (qd, *J* = 6.1, 4.6 Hz, 1H), 4.23 – 4.16 (m, 2H), 4.09 (dd, *J* = 9.6, 4.6 Hz, 1H), 4.04 (d, *J* = 5.3 Hz, 2H), 3.99 – 3.95 (m, 2H), 3.60 – 3.50 (m, 2H), 2.22 (s, 3H), 2.21 (s, 3H), 1.47 (s, 3H), 1.41 (s, 3H) ppm.^{13}C NMR (101 MHz, CD_3OD) δ: 156.8, 156.3, 134.1, 134.0, 130.5, 130.5, 127.2, 127.0, 116.3, 115.8, 111.1, 111.0, 109.8, 88.3, 87.9, 74.2, 69.5, 69.2, 68.6, 66.9, 53.7, 26.9, 25.6, 16.2 ppm. HRMS (ESI): m/z $[M + H]^+$ calculated for $C_{25}H_{30}O_5N_3$: 452.2180; found: 452.2182.

(*R*)-3-(4-((4-((*R*)-3-amino-2-hydroxypropoxy)-3-methylphenyl)ethynyl)-2-methylphenoxy)propane-1,2-diol (1'bb)

A solution of the starting azide (**17bb**, 36 mg, 0.08 mmol) and PPh_3 (36 mg, 0.14 mmol) in THF (1mL) was prepared and 11 μL of H_2O were added. The resulting mixture was stirred 18 h, evaporated and purified by column chromatography (eluted with a gradient of 0-20% MeOH in DCM). The product was used without further purification.

The starting crude amine (55 mg, 0.13 mmol) was dissolved in 0.5 mL of MeOH and 0.5 mL of a 1.25 M solution of HCl in MeOH were added. The mixture was stirred 1 h, diluted with water (10mL), and extracted with DCM (2x10 mL). The resulting aqueous layer was concentrated *in vacuo* to obtain 37 mg (68%) of the expected hydrochloride **1'bb**.

^{1}H NMR (400 MHz, CD_3OD) δ: 7.32 – 7.26 (m, 4H), 6.94 – 6.90 (m, 2H), 4.27 – 4.21 (m, 1H), 4.14 – 4.00 (m, 5H), 3.78 – 3.68 (m, 2H), 3.30 – 3.26 (m, 1H), 3.11 (dd, *J* = 12.7, 9.0 Hz, 1H), 2.25 (bs, 6H) ppm. ^{13}C NMR (101 MHz, CD_3OD) δ: 158.4, 157.8, 134.6, 134.5, 131.4, 131.4, 128.2, 128.1, 117.5, 116.8, 112.2, 112.1, 88.9, 88.5, 71.8, 70.9, 70.3, 67.4, 64.2, 43.5, 16.2 ppm. HRMS (ESI): m/z $[M + H]^+$ calculated for $C_{22}H_{28}NO_5$: 386.1962; found: 386.1961.

Synthesis of compound (1'ab)

(*R*)-1-azido-3-(4-((4-(((*S*)-2,2-dimethyl-1,3-dioxolan-4-yl)methoxy)phenyl)ethynyl)-2-methylphenoxy)propan-2-ol (17ab)

A solution of the starting material (*S*)-2,2-dimethyl-4-((2-methyl-4-((3-methyl-4-(((*R*)-oxiran-2-yl)methoxy)phenyl)ethynyl)phenoxy)methyl)-1,3-dioxolane (**12ab,** 60 mg, 0.15 mmol), sodium azide (119 mg, 1.84 mmol), 4-nitrobenzoic acid (26 mg, 0.15 mmol) and 15-crown-5 (34 µL, 0.16 mmol) was prepared under N_2 atm. in 2 mL of dry DMF. The reaction was heated up to 100 ºC and stirred until no starting material was observed. Then 2 mL of saturated solution of $NaHCO_3$ were added dropwise and the resulting mixture was diluted with water (15 mL) and extracted with EtOAc (3x15 mL). The combined organic extracts were dried over anh. $MgSO_4$ and concentrated in vacuo to obtain 69 mg (100%) of the desired product **17ab**. Mp = 85 - 87 ºC. $[\alpha]_D$ (*c* 0.95, $CHCl_3$) = +19.57. IR (Film): ν_{max} = 2919, 2870, 2097, 1509, 1246, 833 cm^{-1}. ^1H NMR (400 MHz, $CDCl_3$) δ: 7.45 – 7.41 (m, 2H), 7.33 – 7.31 (m, 2H), 6.89 – 6.86 (m, 2H), 6.77 (d, *J* = 9.0 Hz, 1H), 4.48 (quint, *J* = 5.9 Hz, 1H), 4.23 – 4.15 (m, 2H), 4.08 – 4.02 (m, 3H), 3.95 (dd, *J* = 9.5, 5.8 Hz, 1H), 3.90 (dd, *J* = 8.5, 5.8 Hz, 1H), 3.60 – 3.50 (m, 2H), 2.21 (s, 3H), 1.46 (s, 3H), 1.41 (s, 3H) ppm. ^{13}C NMR (101 MHz, $CDCl_3$) δ: 158.5, 156.3, 134.0, 133.0, 130.5, 127.0, 116.3, 116.1, 114.7, 111.1, 110.0, 88.2, 88.0, 74.0, 69.5, 69.2, 68.9, 66.9, 53.7, 26.9, 25.5, 16.2 ppm. HRMS (ESI): m/z $[M + H]^+$ calculated for C24H28O5N3: 438.2024; found: 438.2025.

(*R*)-3-(4-((4-((*R*)-3-amino-2-hydroxypropoxy)-3-methylphenyl)ethynyl)phenoxy)propane-1,2-diol (1'ab)

A solution of the starting azide (**17ab**, 30 mg, 0.07 mmol) and PPh_3 (31 mg, 0.12 mmol) in THF (1mL) was prepared and 11 µL of H_2O were added. The resulting mixture was stirred 18 h, evaporated and purified by column chromatography (eluted with a gradient of 0-20% MeOH in DCM). The product was used without further purification.

The starting crude amine (32 mg, 0.07 mmol) was dissolved in 0.5 mL of MeOH and 0.5 mL of a 1.25 M solution of HCl in MeOH were added. The mixture was stirred 1 h, diluted with water (10mL), and extracted with DCM (2x10 mL). The resulting aqueous layer was concentrated in vacuo to obtain 23 mg (79%) of the expected hydrochloride **1'ab**. ^1H NMR (400 MHz, CD_3OD) δ: 7.44 – 7.35 (m, 2H), 7.32 – 7.24 (m, 2H), 6.97 – 6.92 (m, 2H), 6.90 (d, *J* = 8.4 Hz, 1H), 4.28 – 4.18 (m, 1H), 4.13 – 3.94 (m, 5H), 3.73 – 3.62 (m, 2H), 3.26 (dd, *J* = 12.9, 3.2 Hz, 1H), 3.08 (dd, *J* = 12.8, 9.1 Hz, 1H), 2.23 (s, 3H) ppm. ^{13}C NMR (101 MHz, CD_3OD) δ: 160.3, 157.9, 134.6, 133.8, 131.5, 128.1, 117.3, 117.2, 115.7, 112.2, 88.8, 88.6, 71.7, 70.9, 70.4, 67.4, 64.1, 43.5, 16.2 ppm. HRMS (ESI): m/z $[M + H]^+$ calculated for $C_{21}H_{26}NO_5$: 372.1805; found: 372.1807.

ABBREVIATIONS

DMF: dimethyl formamide. ACN: acetonitrile. Ts: *p*-toluensulfonyl. TMS: trimethylsilyl. dppe: ethylenebis(diphenylphosphine)

References

1. Imamura, Y. et al. An imaging agent to detect androgen receptor and its active splice variants in prostate cancer. *JCI Insight* 1, (2016).

2. Kojima, T. & Hiraoka, S. Selective alternate derivatization of the hexaphenylbenzene framework through a thermodynamically controlled halogen dance. *Org. Lett.* 16, 1024–1027 (2014).

3. Cram, D. J. & Allinger, N. L. Macro Rings. XIII. Synthesis and Properties of 1,7-Cyclododecadiyne and Related Compounds1. *J. Am. Chem. Soc.* 78, 2518–2524 (1956).

4. Shen, R. et al. Facile regio- and stereoselective hydrometalation of alkynes with a combination of carboxylic acids and group 10 transition metal complexes: selective hydrogenation of alkynes with formic acid. J*. Am. Chem. Soc.* 133, 17037–17044 (2011).
