# Supplementary material for: Rational optimization of a transcription factor activation domain inhibitor
Source: Nat Struct Mol Biol. 2023 Dec 4;30(12):1958–69. doi: 10.1038/s41594-023-01159-5 (PMC10716049; doi:10.1038/s41594-023-01159-5)
Supplement: Supplementary file 2 — Reporting Summary [file 41594_2023_1159_MOESM2_ESM.pdf]

nature portfolio

Corresponding author(s): Marianne D. Sadar, Antoni Riera, Denes Hnisz and Xavier Salvatella

Last updated by author(s): Sep 4, 2023

Reporting Summary

Nature Portfolio wishes to improve the reproducibility of the work that we publish. This form provides structure for consistency and transparency in reporting. For further information on Nature Portfolio policies, see our Editorial Policies and the Editorial Policy Checklist.

Statistics

For all statistical analyses, confirm that the following items are present in the figure legend, table legend, main text, or Methods section.

n/a	Confirmed	
☐	☒	The exact sample size (*n*) for each experimental group/condition, given as a discrete number and unit of measurement
☐	☒	A statement on whether measurements were taken from distinct samples or whether the same sample was measured repeatedly
☐	☒	The statistical test(s) used AND whether they are one- or two-sided<br>*Only common tests should be described solely by name; describe more complex techniques in the Methods section.*
☒	☐	A description of all covariates tested
☒	☐	A description of any assumptions or corrections, such as tests of normality and adjustment for multiple comparisons
☐	☒	A full description of the statistical parameters including central tendency (e.g. means) or other basic estimates (e.g. regression coefficient) AND variation (e.g. standard deviation) or associated estimates of uncertainty (e.g. confidence intervals)
☐	☒	For null hypothesis testing, the test statistic (e.g. *F*, *t*, *r*) with confidence intervals, effect sizes, degrees of freedom and *P* value noted<br>*Give P values as exact values whenever suitable.*
☒	☐	For Bayesian analysis, information on the choice of priors and Markov chain Monte Carlo settings
☒	☐	For hierarchical and complex designs, identification of the appropriate level for tests and full reporting of outcomes
☐	☒	Estimates of effect sizes (e.g. Cohen's *d*, Pearson's *r*), indicating how they were calculated

Our web collection on statistics for biologists contains articles on many of the points above.

Software and code

Policy information about availability of computer code

Data collection	NMR data was acquired with TOPSPIN 4.0.8 (Bruker) and processed with NMRPipe 212_64 and qMDD 3.2. FTIR data was acquired with OPUS MIR Tensor 27. MD simulations were run with GROMACS 2019.270-72 patched with PLUMED v2.6.0. RNA-seq data was quality-checked with FASTQC 0.11.9 and aligned to the Homo sapiens genome hg19 with STAR aligner v.2.7.5a with standard settings.
Data analysis	NMR data was analyzed with CcpNmr Analysys 2.4.2 and delta2D (online). FTIR data was analyzed with Peak Fit 4.2.Live cell microscopy data was analyzed with Fiji imaging software (ImageJ 1.52p), LAS-AF and LAS-X (2.5.6, Leica). FRAP data was analyzed with EasyFRAP (online). BioID-MS data was analyzed with SAINTq. The images used for the granularity were analyzed with ZEN Blue 3.2. The LNCaP dose response curves were processed with the DRC package in R. MD trajectories were analyzed with python packages MDtraj, numpy and pyblock. The differential expression analysis was conducted with the DESeq2 R/bioconductor package and the gene set enrichment with packages fgsea and DOSE.

For manuscripts utilizing custom algorithms or software that are central to the research but not yet described in published literature, software must be made available to editors and reviewers. We strongly encourage code deposition in a community repository (e.g. GitHub). See the Nature Portfolio guidelines for submitting code & software for further information.

Data

Policy information about availability of data

All manuscripts must include a data availability statement. This statement should provide the following information, where applicable:

- Accession codes, unique identifiers, or web links for publicly available datasets
- A description of any restrictions on data availability
- For clinical datasets or third party data, please ensure that the statement adheres to our policy

The RNA-sequencing data has been deposited in the NCBI GEO database (https://www.ncbi.nlm.nih.gov/geo/) with accession codes GSE206853 and GSE232849. The NMR assignments for constructs 441-558 and the AR AD have been deposited in the BMRB (https://bmrb.io/) with accession codes 51476 and 51480, respectively. The molecular dynamics simulation trajectories, GROMACS input files, and analysis code have been deposited in Zenodo (https://doi.org/10.5281/zenodo.8210256). The raw data used to produce all figures is available as Source Data files.

Human research participants

Policy information about studies involving human research participants and Sex and Gender in Research.

Reporting on sex and gender	Not relevant to this study
Population characteristics	Not relevant to this study
Recruitment	Not relevant to this study
Ethics oversight	Not relevant to this study

Note that full information on the approval of the study protocol must also be provided in the manuscript.

Field-specific reporting

Please select the one below that is the best fit for your research. If you are not sure, read the appropriate sections before making your selection.

☒ Life sciences ☐ Behavioural & social sciences ☐ Ecological, evolutionary & environmental sciences

For a reference copy of the document with all sections, see nature.com/documents/nr-reporting-summary-flat.pdf

Life sciences study design

All studies must disclose on these points even when the disclosure is negative.

Sample size	No statistical method was used to determine sample size. Sample size was determined by the number of relevant experimental observations e.g. number of cells, and always larger than 1.
Data exclusions	No data was excluded in the studies.
Replication	Biological replicates were used in all experiments in cells.
Randomization	Randomization was considered not necessary in this study.
Blinding	Blinding was considered not necessary in this study.

Reporting for specific materials, systems and methods

We require information from authors about some types of materials, experimental systems and methods used in many studies. Here, indicate whether each material, system or method listed is relevant to your study. If you are not sure if a list item applies to your research, read the appropriate section before selecting a response.

Materials & experimental systems

n/a	Involved in the study
☐	☒ Antibodies
☐	☒ Eukaryotic cell lines
☒	☐ Palaeontology and archaeology
☐	☒ Animals and other organisms
☒	☐ Clinical data
☒	☐ Dual use research of concern

Methods

n/a	Involved in the study
☒	☐ ChIP-seq
☒	☐ Flow cytometry
☒	☐ MRI-based neuroimaging

Antibodies

Antibodies used	Androgen Receptor [ER179(2)] Abcam Cat#ab108341<br>Streptavidin antibody, Alexa Fluor™ 488 conjugate ThermoScientific Cat #S11223<br>Alexa Fluor 488 Goat anti-Rabbit IgG (H+L) ThermoScientific Cat #A11008<br>Androgen Receptor (441) SCBT Cat #sc-7305<br>STAR 635P Abberior Cat #ST635P-1001<br>Goat anti-Rabbit IgG (H+L) Secondary Antibody, HRP Invitrogen Cat #65-6120<br>Goat anti-Mouse IgG (H+L) Secondary Antibody, HRP Invitrogen Cat #G-21040<br>Nup153 antibody (QE5) Abcam Cat #ab24700<br>Med1 antibody Abcam Cat #ab64965<br>ARID1A/BAF250A (D2A8U) CellSignal #12354<br>GADPH Abcam Cat#ab59164<br>anti-mouse RD-680 conjugated LI-COR CatR #926-68072<br>anti-rabbit CW-800 conjugated LI-COR CatR #926-32211
Validation	All antibodies used in this study are commercial, validated by the manufacturer for the relevant applications and widely used, as described in the following websites:<br><br>https://www.abcam.com/products/primary-antibodies/androgen-receptor-antibody-er1792-chip-grade-ab108341.html<br>https://www.thermofisher.com/order/catalog/product/es/en/S11223<br>https://www.thermofisher.com/antibody/product/Goat-anti-Rabbit-IgG-H-L-Cross-Adsorbed-Secondary-Antibody-Polyclonal/A-11008<br>https://www.scbt.com/p/ar-antibody-441<br>https://abberior.shop/abberior-STAR-635P<br>https://www.thermofisher.com/antibody/product/Goat-anti-Rabbit-IgG-H-L-Secondary-Antibody-Polyclonal/65-6120<br>https://www.thermofisher.com/antibody/product/Goat-anti-Mouse-IgG-H-L-Cross-Adsorbed-Secondary-Antibody-Polyclonal/G-21040<br>https://www.abcam.com/products/primary-antibodies/nup153-antibody-qe5-ab24700.html<br>https://www.abcam.com/products/primary-antibodies/trap220med1-antibody-ab64965.html<br>https://www.cellsignal.com/products/primary-antibodies/arid1a-baf250a-d2a8u-rabbit-mab/12354<br>https://www.abcam.com/products/primary-antibodies/gapdh-antibody-ff26a-ab59164.html<br>https://www.licor.com/bio/reagents/irdye-680rd-donkey-anti-mouse-igg-secondary-antibody<br>https://www.licor.com/bio/reagents/irdye-800cw-goat-anti-rabbit-igg-secondary-antibody

Eukaryotic cell lines

Policy information about cell lines and Sex and Gender in Research

Cell line source(s)	HEK293T cells ATCC CRL-3216<br>PC3 cells ATCC CRL-1435<br>LNCaP clone FGC ATCC CRL-1740<br>LNCaP95-D3 Leung et al., 2021 (generated by the laboratory of M. D. Sadar)<br>Hela AR-eGFP 24Q Piol et al., 2023 (generated by the laboratory of M. Pennuto)
Authentication	None of the cells were authenticated
Mycoplasma contamination	All cell lines tested negative
Commonly misidentified lines<br>(See ICLAC register)	No commonly misidentified cell lines were used in this study.

Animals and other research organisms

Policy information about studies involving animals; ARRIVE guidelines recommended for reporting animal research, and Sex and Gender in Research

Laboratory animals	Six to eight-weeks-old male mice (NOD-scid IL2Rgammanull).

Wild animals	No wild animals were used in this study.
Reporting on sex	Findings apply to only male animals
Field-collected samples	Not field collected samples were used in this study.
Ethics oversight	University of British Columbia Animal Care Committee (A18-0077)

Note that full information on the approval of the study protocol must also be provided in the manuscript.
