# Supplementary figures and images for: Rational optimization of a transcription factor activation domain inhibitor
Source: Nat Struct Mol Biol. 2023 Dec 4;30(12):1958–69. doi: 10.1038/s41594-023-01159-5 (PMC10716049; doi:10.1038/s41594-023-01159-5)

Fig. 3G

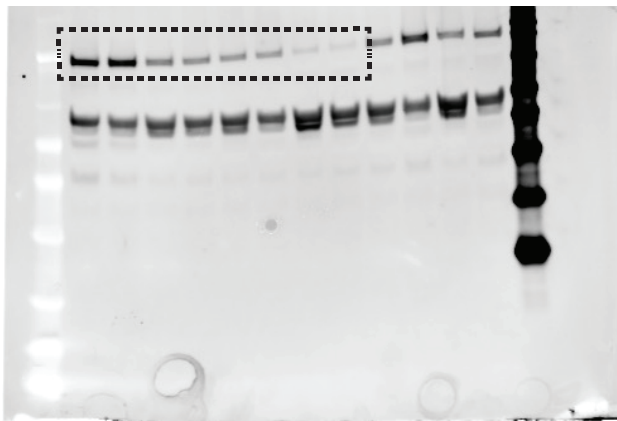

Extended Data Fig. 3C

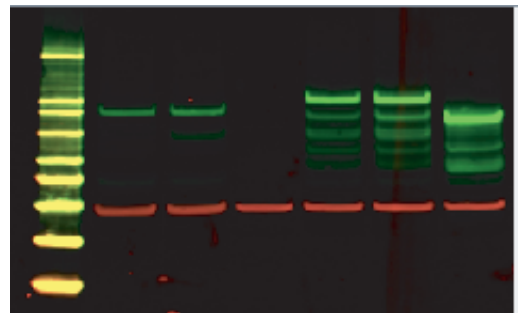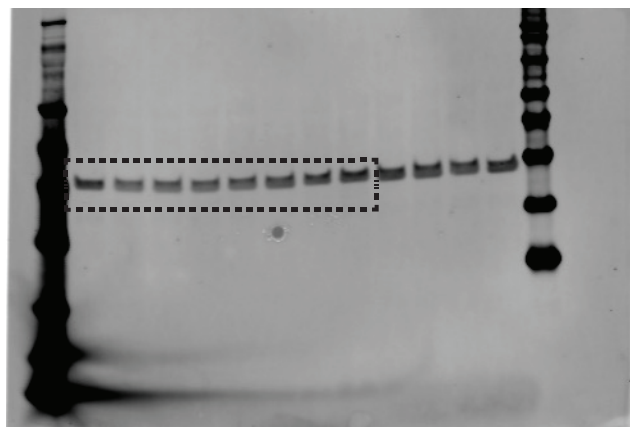

Extended Data Fig. 6A

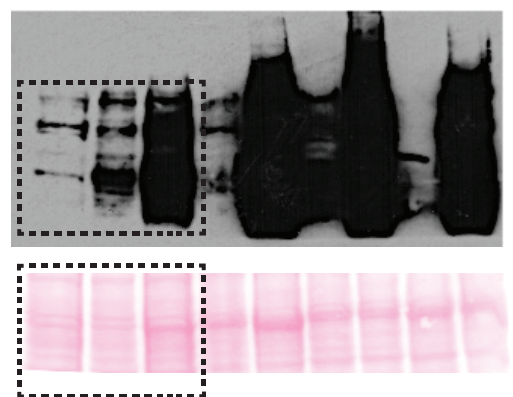

Extended Data Fig. 3E

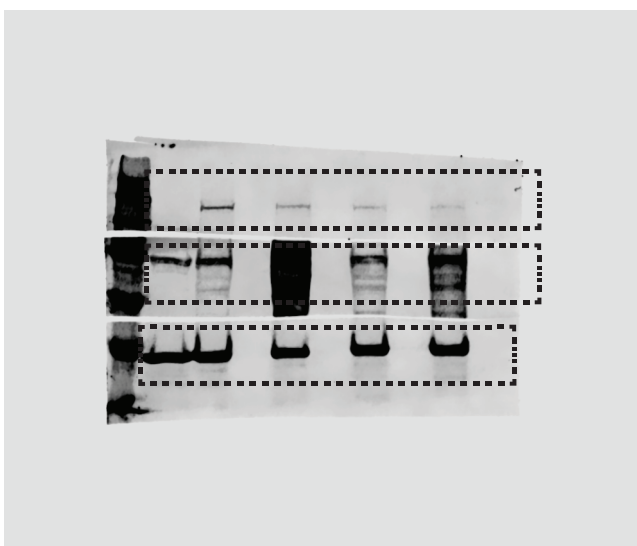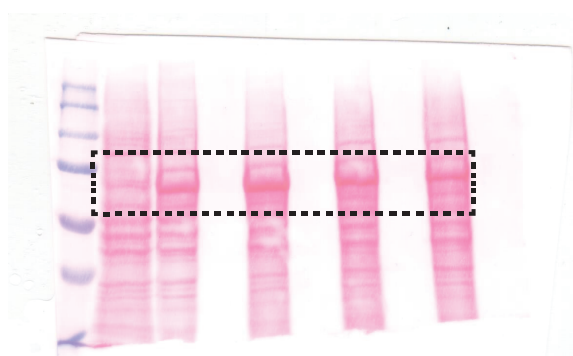

Supplement: Supplementary file 27 — Unprocessed western blots. [file 41594_2023_1159_MOESM27_ESM.pdf]
