# Supplementary material for: Rational optimization of a transcription factor activation domain inhibitor
Source: Nat Struct Mol Biol. 2023 Dec 4;30(12):1958–69. doi: 10.1038/s41594-023-01159-5 (PMC10716049; doi:10.1038/s41594-023-01159-5)
Supplement: Supplementary file 3 — Peer Review File [file 41594_2023_1159_MOESM3_ESM.pdf]

Peer Review Information

Journal: Nature Structural & Molecular Biology
Manuscript Title: Rational optimization of a transcription factor activation domain inhibitor
Corresponding author name(s): Xavier Salvatella, Denes Hnisz, Antoni Riera, Marianne D. Sadar

Reviewer Comments & Decisions:

Decision Letter, initial version:

Message: 17th Apr 2023

Dear Dr. Salvatella,

Thank you again for submitting your manuscript "Rational optimization of a transcription factor activation domain inhibitor". I sincerely apologize for the delay in responding, which resulted from the difficulty in obtaining suitable referee reports. Nevertheless, we now have comments (below) from the 4 reviewers who evaluated your paper. In light of those reports, we remain interested in your study and would like to see your response to the comments of the referees, in the form of a revised manuscript.

You will see that while all the reviewers appreciate the work, Reviewers #1 and #4 highlight the importance of linking the phase separation and the AR targeting aspects of the study, by performing additional experiments to investigate the effects of drug targeting on AR condensates. Editorially, we agree that this will strengthen the impact of the manuscript. Additionally, in response to Reviewer #3, please include discussion of the observed mild potency in the xenograft model. Please be sure to address/respond to all concerns of the referees in full in a point-by-point response and highlight all changes in the revised manuscript text file. If you have comments that are intended for editors only, please include those in a separate cover letter.

We are committed to providing a fair and constructive peer-review process. Do not hesitate to contact us if there are specific requests from the reviewers that you believe are technically impossible or unlikely to yield a meaningful outcome.

We expect to see your revised manuscript within 6 weeks. If you cannot send it within this time, please contact us to discuss an extension; we would still consider your revision, provided that no similar work has been accepted for publication at NSMB or published elsewhere.

natureresearch

As you already know, we put great emphasis on ensuring that the methods and statistics reported in our papers are correct and accurate. As such, if there are any changes that should be reported, please submit an updated version of the Reporting Summary along with your revision.

Please follow the links below to download these files:

Reporting Summary:
https://www.nature.com/documents/nr-reporting-summary.pdf

Please note that the form is a dynamic 'smart pdf' and must therefore be downloaded and completed in Adobe Reader.

When submitting the revised version of your manuscript, please pay close attention to our href="https://www.nature.com/nature-portfolio/editorial-policies/image-integrity">Digital Image Integrity Guidelines. and to the following points below:

-- that unprocessed scans are clearly labelled and match the gels and western blots presented in figures.
-- that control panels for gels and western blots are appropriately described as loading on sample processing controls
-- all images in the paper are checked for duplication of panels and for splicing of gel lanes.

Finally, please ensure that you retain unprocessed data and metadata files after publication, ideally archiving data in perpetuity, as these may be requested during the peer review and production process or after publication if any issues arise.

Please note that all key data shown in the main figures as cropped gels or blots should be presented in uncropped form, with molecular weight markers. These data can be aggregated into a single supplementary figure item. While these data can be displayed in a relatively informal style, they must refer back to the relevant figures. These data should be submitted with the final revision, as source data, prior to acceptance, but you may want to start putting it together at this point.

SOURCE DATA: we urge authors to provide, in tabular form, the data underlying the graphical representations used in figures. This is to further increase transparency in data reporting, as detailed in this editorial (http://www.nature.com/nsmb/journal/v22/n10/full/nsmb.3110.html). Spreadsheets can be submitted in excel format. Only one (1) file per figure is permitted; thus, for multi-paneled figures, the source data for each panel should be clearly labeled in the Excel file; alternately the data can be provided as multiple, clearly labeled sheets in an Excel file. When submitting files, the title field should indicate which figure the source data pertains to. We encourage our authors to provide source data at the revision stage, so that they are part of the peer-review process.

Data availability: this journal strongly supports public availability of data. All data used in accepted papers should be available via a public data repository, or alternatively, as Supplementary Information. If data can only be shared on request, please explain why in your Data Availability Statement, and also in the correspondence with your editor. Please

note that for some data types, deposition in a public repository is mandatory - more information on our data deposition policies and available repositories can be found below: https://www.nature.com/nature-research/editorial-policies/reporting-standards#availability-of-data

We require deposition of coordinates (and, in the case of crystal structures, structure factors) into the Protein Data Bank with the designation of immediate release upon publication (HPUB). Electron microscopy-derived density maps and coordinate data must be deposited in EMDB and released upon publication. Deposition and immediate release of NMR chemical shift assignments are highly encouraged. Deposition of deep sequencing and microarray data is mandatory, and the datasets must be released prior to or upon publication. To avoid delays in publication, dataset accession numbers must be supplied with the final accepted manuscript and appropriate release dates must be indicated at the galley proof stage.

While we encourage the use of color in preparing figures, please note that this will incur a charge to partially defray the cost of printing. Information about color charges can be found at http://www.nature.com/nsmb/authors/submit/index.html#costs

Nature Structural & Molecular Biology is committed to improving transparency in authorship. As part of our efforts in this direction, we are now requesting that all authors identified as ‘corresponding author’ on published papers create and link their Open Researcher and Contributor Identifier (ORCID) with their account on the Manuscript Tracking System (MTS), prior to acceptance. This applies to primary research papers only. ORCID helps the scientific community achieve unambiguous attribution of all scholarly contributions. You can create and link your ORCID from the home page of the MTS by clicking on ‘Modify my Springer Nature account’. For more information please visit please visit www.springernature.com/orcid.

Please use the link below to submit your revised manuscript and related files:

[redacted]

Note: This URL links to your confidential home page and associated information about manuscripts you may have submitted, or that you are reviewing for us. If you wish to forward this email to co-authors, please delete the link to your homepage.

We look forward to seeing the revised manuscript and thank you for the opportunity to review your work.

Sincerely,
Sara

Sara Osman, Ph.D.
Associate Editor
Nature Structural & Molecular Biology

Referee expertise:

Referee #1: Drug discovery, condensates

Referee #2: NMR, condensates

Referee #3: Hormone receptor function

Referee #4: LLPS

Reviewers' Comments:

Reviewer #1:
Remarks to the Author:
Summary and high-level comments

Authors show that full-length androgen receptor (AR) and the disease-relevant splice variant AR-V7 can form nuclear clusters when ectopically expressed in cells. While this has been previously described (see Xie et al, Nat Chem Biol, 2022, DOI: 10.1038/s41589-022-01151-y and Thiyagarajan, PNAS, 2023, DOI: 10.1073/pnas.2211832120), the authors go beyond what currently reported in the literature by using NMR to probe the N-terminal activation domain of AR and identify residues required for intermolecular interactions. The authors demonstrate a role for tyrosine residues in AR and AR-V7 phase separation using in-cell and purified methods. Similarly, the authors provide data supporting a role for helical propensity in promoting phase-separation of the activation domain of AR in purified settings and a cytoplasmic AR construct in cells. The authors then discuss the optimization of a small molecule targeting the AR activation domain, building on De Mol et al, ACS Chem Biol, 2016. The authors use a luciferase reporter system to measure AR activity as well as proliferation in AR and AR-V7 cell line models. Finally, the authors use RNAseq to evaluate transcriptional changes caused by compound 1ae (reported in this manuscript) and EPI-001, and compared the anti-tumor activity of 1ae and enzalutamide in a tumor xenograft mouse model.

The manuscript is well written, the experiments are technically sound, and their interpretation is well aligned with conclusions presented, with exceptions described below.

One important limitation of the manuscript is that the two main topics presented, AR condensates (Fig. 1-3) and small molecule modulators of AR (Fig. 4-6), are disconnected, with no experiments addressing the potential link between AR condensates and activity of the inhibitor compounds discussed. This significantly limits the impact and broad applicability of the work presented. The importance of this work would increase significantly if authors could address experimentally if and how the AR inhibitors discussed affect AR condensates. In absence of such data, the work presented provides limited advance for either field.

The authors conclude the abstract stating “These results establish a generalizable framework to target small molecules to the activation domains of oncogenic transcription factors”. It is unclear what elements of this work are indeed generalizable and no experiments seem to directly support this claim.

Moreover, the authors should further describe how their work complements studies recently published on AR condensates and small molecule modulators of AR. See Xie et al,

Nat Chem Biol, 2022 (DOI: 10.1038/s41589-022-01151-y) which is cited in the manuscript, and Thiyagarajan et al, PNAS, 2023 (DOI: 10.1073/pnas.2211832120) which is not – publication of the latter was recent.

Major comments

The authors use ectopic expression of eGFP-tagged AR to study phase-separation of AR in cell systems. It is known that concentration / expression levels can affect phase-separation behavior. It would be important to establish how the levels of eGFP-tagged AR used compare to endogenous AR and splice variants in disease-relevant cell models.

Page 4, last paragraph – the authors introduce the L26P (WT*) mutation to improve solubility of the AR constructs used, presumably easing their use in experiments in purified settings. It would be important to discuss or test how this mutation may affect full-length AR function in cells. Based on Fig. 3C, 3G, and Supplementary Fig. 4H, it appears that this mutation affects AR condensate properties in purified settings.

Page 7, bottom – the authors discuss the link between hormone binding and AR phase separation. Data presented in Fig. 3F and 3G and Supplementary Fig. 4I-K are not directly connected (L26 mutation versus FQNLF deletion) and overall do not meaningfully advance our understanding for the role of hormone binding in AR phase separation. This paragraph should be rephrased or supplemented with in-cell and/or biophysical data describing how hormone binding promotes AR LBD and AD interactions and phase separation. It is not obvious how this relates to the role of Y residues and helical propensity described in the manuscript.

Page 7 and 8 – Supplementary Fig. 5I shows that compound 1ae was significantly more potent in LNCaP (full-length AR) compared to LNCaP95 (AR-V7) cells. The authors should discuss this and provide a more direct and quantitative comparison of potency in the activity reporter for the analogs presented in AR versus AR-V7 cell models (IC50 numbers for both, rather than bar graphs for AR-V7).

Page 9 – the authors state that "Compound 1ae is a potent inhibitor of AR-dependent transcription and tumor growth". While they provide data supporting a role for compound 1ae in AR-dependent transcription and tumor growth, the authors do not provide data supporting its specificity beyond the lack of activity in a non-AR-driven reporter (Supplementary Fig. 5H). It would be important to show that the anti-tumor effects of 1ae are indeed specific to AR-dependent tumor growth.

Minor comments

It would be important to clarify if native (belonging to endogenous AR) or an ectopic nuclear localization signal (NLS) was used to generate constructs for in-cell data. If an ectopic NLS was used, the relevance of the results for describing the behavior of endogenous AR and splice variants should be addressed.

Page 4, second paragraph – the authors use the word "sticky" to describe residues identified by NMR. The same is true in Fig. 1C. It would be helpful to better define what it is meant rather than use ambiguous language.

Page 5, second paragraph – the authors state "These results collectively reveal that AR

phase separation is driven by tyrosine residues within the AR AD”. Based on the data presented, it would be more accurate to state that the Y residues described contribute to phase-separation of WT* AR AD. Figure 1 does not include full-length AR, nor the truly WT receptor, and does not address whether Y residues are sufficient for phase-separation. Thus, the use of “driven” seems incorrect, or at least imprecise.

Page 7 – the authors study the role of helicity in in-cell phase-separation of cytoplasmic AR constructs. It is not clear why absence of DNA binding and NLS in these constructs was deemed necessary (hence their cytoplasmic localization), given that the data presented thus far address the role of nuclear AR condensates. This point should be clarified.

Similarly, the authors should specifically address (at least with text) the potential functional relevance of cytoplasmic versus nuclear condensates and the possible relationship between the two.

Page 9 – it would be important to clarify how compound 1ae decreases AR levels as revealed by Western blot analysis (Fig. 6G).

Page 10 – the authors state “These results suggest that 1ae has in vivo antitumor activity in CRPC xenograft models, and outperforms enzalutamide”. It would be important to mention that the latter point only applies to one of the two models tested.

There is a typo in Reporting summary / Animals and other research organisms / Reporting on sex, “make” seems incorrect.

Reviewer #2:
Remarks to the Author:
This is a highly interesting manuscript in which the authors combine phase separation/condensation experiments in vitro and in cells with NMR-based structural/interaction analysis to better understand the cluster formation of the Androgen receptor transcription factor and then use this information to optimize a small molecule to interfere with its phase separation. Additionally, it is shown that the optimized small molecule inhibits androgen receptor-dependent transcriptional programs, and has antitumorigenic effect in models of castration-resistant prostate cancer in cells and in vivo. The data are of high quality, the manuscript is well written and nicely illustrated. Overall, this is a highly interesting and well performed study providing unique insight into transcription-associated phase separation and small molecules targeting biomolecular condensation.

I just have a few minor suggestions for improvement:

What is in the region from 450-470, which is strongly broadened? Is it important for phase separation and transcriptional activation?

Fig. 1e: should be mM instead of uM

Please specify “weak” helical propensity for the two motifs 232DNAKELCKA240 and 351LDEAAAYQS359, i.e. % of helical propensity

Reviewer #3:
Remarks to the Author:
In this manuscript, Basu, Cristoval, and colleagues describe a compound targeting the intrinsically disordered activation domain of the androgen receptor. The compound exhibit moderate activity in vivo in a model of castration-resistant PCa (driven by AR-v7, lacking the LBD domain). The author concludes targeting the IRD is a generalizable strategy. The manuscript is interesting and well done. I would argue that if the authors wanted to highlight the strategy and its potential generalization, the choice of targeting AR seems a bit odd, considering the plethora of therapies developed against this target (including AR-v7). I would have expected an additional TF targeted following this strategy (something truly untargetable) since drugs against hormone receptors make up a sizable portion of all FDA-approved treatments. Furthermore, the activity in the LNCAP95 xenograft is not impressive. At a minimum, I would expect a second xenograft model mimicking a LBD-ablated AR.

Reviewer #4:
Remarks to the Author:
This work identified a sticky region in the activation domain (AD) of androgen receptor (AR) using structure studies and phase separation assays. The authors modified an existing drug to better target this sticky region and found a version that further reduces AR mediated transcription and inhibits tumor growth. This is a very nice example illustrating how phase separation can be used as drug targets. Overall, this study is well carried out. My major concern is that it is not clear whether the drug disrupts/targets AR phase separation. The authors did not show how AR condensates in vitro and in cells respond to the drugs. It is possible that the drug affects other aspects of AR function other than phase separation. For example, the sticky region in the AD is important for both AR phase separation and nuclear import. To decouple these two effects, the authors did use the AR-V7 that localizes to the nucleus to measure effect of drugs on transcription. My problem with this control is that for the WT the major target can still be nuclear import. Data to show how much nuclear transport of the WT is affected by the drugs would be informative. Without these data to determine whether and how much AR phase separation is affected by the drug, the BioID and NMR data that reveal AR self-interaction and interaction with other transcription factors would be sufficient to guide the drug design and interpret the drug effect and hence there is no need for the phase separation studies.
Other minor concerns:

1. Two types of AR condensates are reported: one in the cytoplasm and one in the nucleus. More discussion about similarities and difference in the formation, possible compositions, and functions of those two condensates would be useful. Does the drug disrupt one or both?
2. What is the relevant importance of the drug target region to phase separation comparing to other regions? Since other regions of AR are known to be important for its phase separation, instead of using AD and mutants for in vitro phase separation, using the full-length and mutants would be more relevant.
3. Also when testing the importance of LBD and AD binding for phase separation, why not use the full-length protein and its truncations? And test direct effect of hormone binding

on phase separation in vitro? This would be better than adding LBD to AD. If it’s due to technical difficulty, the authors should point out and discuss the limitations. For example, the AD shows LCST behavior, the full protein may not.
4. Figure 1G, RNAPII-CTD droplets are smaller for the 22YtoS mutant. Is that representative? In other words, does AD passively partition or actively affect RNAPII-CTD phase behavior? This information would be useful for understanding the nature of AR condensates at transcription sites.

Author Rebuttal to Initial comments

Response to reviewers

Reviewer #1

Summary and high-level comments

Authors show that full-length androgen receptor (AR) and the disease-relevant splice variant AR-V7 can form nuclear clusters when ectopically expressed in cells. While this has been previously described (see Xie et al, Nat Chem Biol, 2022, DOI: 10.1038/s41589-022-01151-y and Thiyagarajan, PNAS, 2023, DOI: 10.1073/pnas.2211832120), the authors go beyond what currently reported in the literature by using NMR to probe the N-terminal activation domain of AR and identify residues required for intermolecular interactions.

We were pleased to read that Reviewer #1 considers that our work goes beyond what is reported in the literature.

The authors demonstrate a role for tyrosine residues in AR and AR-V7 phase separation using in-cell and purified methods. Similarly, the authors provide data supporting a role for helical propensity in promoting phase-separation of the activation domain of AR in purified settings and a cytoplasmic AR construct in cells. The authors then discuss the optimization of a small molecule targeting the AR activation domain, building on De Mol et al, ACS Chem Biol, 2016. The authors use a luciferase reporter system to measure AR activity as well as proliferation in AR and AR-V7 cell line models. Finally, the authors use RNAseq to evaluate transcriptional changes caused by compound 1ae (reported in this manuscript) and EPI-001, and compared the anti-tumor activity of 1ae and enzalutamide in a tumor xenograft mouse model. The manuscript is well written, the experiments are technically sound, and their interpretation is well aligned with conclusions presented, with exceptions described below.

We were also pleased to read that Reviewer #1 considers the experiments technically sound and the interpretation of the results well aligned with the conclusions. We trust that, with the additional work introduced in the revised manuscript, this Reviewer will consider the work suitable for publication in *Nat. Struct. Mol. Biol.*

One important limitation of the manuscript is that the two main topics presented, AR condensates (Fig. 1-3) and small molecule modulators of AR (Fig. 4-6), are disconnected, with no experiments addressing the potential link between AR condensates and activity of the inhibitor compounds discussed. This significantly limits the impact and broad applicability of the work presented. The importance of this work would increase significantly if authors could address experimentally if and how the AR inhibitors discussed affect AR condensates. In absence of such data, the work presented provides limited advance for either field.

To address this concern of the Reviewer, we have carried out experiments that show that the AR AD condensates described in the first part of the manuscript (Figs. 1-3) are the target of the AR modulators described in the second (Figs. 4-6) and, in addition, that characterising the mechanism of condensation was indeed key for improving the potency, as an AR inhibitor, of the drug-like small molecule initially identified by phenotypic screening (EPI-001).

More specifically, the results that we added to the revised version of the manuscript show that EPI-001 targets AR AD condensates by partitioning in them, shifting the cloud point (Fig. 4A,J), and that changes in its chemical structure aimed at optimising its interaction with aromatic residues (Fig. 4), which are the drivers of condensation (Figs. 1-3), shift the cloud point to a larger extent (Fig. 4J), leading to an increase in potency of AR inhibition (Fig. 4).

In addition, to establish a link between the driving forces of phase separation (Figs. 1-3) and small molecule partitioning, we measured how decreasing the aromatic character of the AR AD by mutation of 8 Tyr residues to Ser in AR mutant 8YtoS, that decreases its propensity to phase separate (Figs. 1D-F and 2A), alters the partitioning of small molecules in the condensates (Fig. 4A): we obtained a lower partitioning of the AR modulators in 8YtoS, in agreement with our hypothesis. This new data directly links the sequence features that drive AR condensates and the ability of small molecules to partition into AR condensates, as requested by the Reviewer.

Finally, we would like to highlight that knowledge that the AR AD condensates are stabilised by hydrophobic interactions between aromatic residues in the helices of Tau-5 (Figs. 1-2) was instrumental for obtaining compound 1ae (Fig. 4-6): it informed our decision to alter the relative position of the aromatic groups in the scaffold (Fig. 4) as well as our choice of substituents for its optimisation as AR inhibitor (Fig. 4H,I). Indeed compound 1ae, bearing an additional aromatic ring and therefore with the capacity to better interact with the target, is the most potent inhibitor (Fig. 5,6).

In summary, our results indicate that knowledge that AR must phase separate to perform its function, of the residue types involved in the phase transition and of the secondary structure that the relevant region of sequence adopts in the condensate can guide drug development, linking the two parts of the manuscript. We acknowledge that the link was too implicit in the original version and, in the revised manuscript, in addition to describing our new results, we explicitly discuss it in the discussion. We would like to thank Reviewer #1 for this request, as it has indeed led to a more compelling manuscript.

The authors conclude the abstract stating “These results establish a generalizable framework to target small molecules to the activation domains of oncogenic transcription factors”. It is unclear what elements of this work are indeed generalizable and no experiments seem to directly support this claim.

Intrinsically disordered targets have not been considered amenable to rational optimisation, understood as the process whereby knowledge of the conformation and the functional interactions of the target is used to modify the initial hits to maximise potency. The work that we present in the manuscript shows that knowledge that the intrinsically disordered phase separates, of the structure adopted upon phase separation, and of the nature of the interactions that drive the transition can be helpful for hit optimisation.

What we meant in this sentence is that, given the results that we present in the manuscript for the AR, a similar approach may be useful for developing modulators of other transcription factors by optimising the chemical structures of compounds identified by phenotypic screening or by developing new screening assays. We agree with Reviewer #1 that this statement is too broad for the abstract, which we have modified as follows: “These results

show that it is possible to rationally design small molecules that target the activation domains of oncogenic transcription factors".

Moreover, the authors should further describe how their work complements studies recently published on AR condensates and small molecule modulators of AR. See Xie et al, Nat Chem Biol, 2022 (DOI: 10.1038/s41589-022-01151-y) which is cited in the manuscript, and Thiyagarajan et al, PNAS, 2023 (DOI: 10.1073/pnas.2211832120) which is not – publication of the latter was recent.

We have added a sentence to the discussion where we explicitly show how the work that we present complements the work presented in the two articles mentioned by Reviewer #1. In it, we emphasize that our approach is complementary to that presented in Xie et al (2022) Nat Chem Biol – based on phenotypic screening for compounds that inhibit condensation – and to that presented in Thiyagarajan et al (2023) PNAS – based on targeting Cys residues in AR AD with covalent inhibitors bearing an electrophilic warhead – because we use a rational approach to improve how the inhibitors interact with their target.

Major comments

The authors use ectopic expression of eGFP-tagged AR to study phase-separation of AR in cell systems. It is known that concentration / expression levels can affect phase-separation behavior. It would be important to establish how the levels of eGFP-tagged AR used compare to endogenous AR and splice variants in disease-relevant cell models.

We agree with Reviewer #1 that this is a relevant point. To ensure the levels of eGFP-AR were the lowest possible, we transfected cells by using PEI rather than lipofectamine and selected for analysis the cells with the lowest expression levels, based on fluorescence emission. Since the efficiency of transfection is heterogeneous determining the levels of eGFP-AR in the cells selected for analysis is challenging but we nevertheless estimated it by measuring the expression levels of a population of cells by Western blotting: on average the levels of eGFP-AR were higher than those obtained for AR in LNCaP (expressing AR) and LNCaP95 cells (expressing AR and AR-v7) but, we think, in the physiological range for the cells selected. These new results are discussed in the relevant section of the manuscript and presented in Supplementary Fig. 3A-C.

Page 4, last paragraph – the authors introduce the L26P (WT*) mutation to improve solubility of the AR constructs used, presumably easing their use in experiments in purified settings. It would be important to discuss or test how this mutation may affect full-length AR function in cells. Based on Fig. 3C, 3G, and Supplementary Fig. 4H, it appears that this mutation affects AR condensate properties in purified settings.

We indeed used the L26P mutation to facilitate the handling of the purified activation domain and, especially, the quantitative analysis of how changes in helical character affect phase separation propensity (Fig. 3C). In the absence of this mutation the droplets lose their liquid character rather quickly, which can make these quantifications inaccurate by confounding changes in phase separation propensity with changes in aggregation propensity.

We introduced it because, according to predictions, it decreases the aggregation propensity of the domain. To show this we have measured how it alters its aggregation as well as the secondary structure and morphology of the aggregates formed by the relevant region of sequence - the motif ^{23}FQNLF^{27} (Supplementary Fig. 2E-H). In addition, we have measured how the mutation preserves the liquid character of the droplets (Supplementary Fig. 2J,K).

The motif harboring this mutation, ^{23}FQNLF^{27}, changes conformation upon AR activation: it is initially disordered but folds into an α-helix upon interaction with the LBD after androgen binding[1]. As proline is a helix-breaker we expect mutation L26P, in addition to preventing aggregation, to prevent this interaction and therefore used this mutant to investigate whether the interaction increases the phase separation propensity of AR in Fig. 3.

We acknowledge that the effect of L26P on the interaction with the LBD, although plausible, has not been shown experimentally and have therefore carried out biophysical experiments to test this hypothesis by removing the motif altogether (Fig. 3F,G). In summary, in the revised manuscript we only use mutation L26P to facilitate the handling of the purified AD (and its mutants) and do not use it to investigate the interdomain interaction. Finally, we would like to clarify that mutation L26P was not introduced in any of the mutants studied in cells; this has also been clarified in the text.

Page 7, bottom – the authors discuss the link between hormone binding and AR phase separation. Data presented in Fig. 3F and 3G and Supplementary Fig. 4I-K are not directly connected (L26 mutation versus FQNLF deletion) and overall do not meaningfully advance our understanding for the role of hormone binding in AR phase separation. This paragraph should be rephrased or supplemented with in-cell and/or biophysical data describing how hormone binding promotes AR LBD and AD interactions and phase separation. It is not obvious how this relates to the role of Y residues and helical propensity described in the manuscript.

We acknowledge that the connection between the results shown in the original Fig. 3F, G and those presented in original Supplementary Fig. 3I-K was indirect. As explained in our response to the previous point of this Reviewer we have addressed it by carrying out additional biophysical experiments. We also acknowledge that we did not present in a sufficiently detailed manner our interpretation of the results described in these figures, from which we conclude that hormone binding causes the phase separation of AR as shown in Supplementary Fig. 4.

We have addressed the latter point by rephrasing this paragraph. In it, we now explain that androgen binding to the LBD causes a collective, allosteric conformational change that exposes two surface patches in this domain that cause AR to oligomerize. It triggers the heterotypic interaction between the ^{23}FQNLF^{27} in the AD and activation function 2 in the LBD[1,2], and the homotypic interaction between a recently identified dimerization interface in the LBD[3].

Both dimerization interfaces increase the propensity of the receptor to phase separate because each of them multiplies by two the number of Tyr valencies that can contribute to the phase separation of the receptor. This effect, due to a decrease in the entropy of the phase transition caused by tethering, is predicted by theory[4], has been reproduced in

coarse-grained molecular simulations of liquid-liquid phase separation[5] and is the basis of the OptoDroplet technology used to increase the phase separation of proteins in cells by fusing them to domains that oligomerize upon exposure to light[6].

It is worth emphasizing that the two dimerization interfaces are independent of each other, indicating that androgen binding can lead to oligomers of a higher order than dimers and that the heterotypic interaction between the AD and the LBD can give rise to one-dimensional arrays of AR molecules, increasing the multivalency of AR even further. We acknowledge that, as stated by the Reviewer, this interpretation is anything but trivial: therefore, to support it, we have improved the scheme illustrating it in Fig. 3H.

Reviewer #1 also makes a valid point about the mechanism by which the helicity of certain regions of sequence of the AD contributes to phase separation propensity. We do not have a complete understanding of this but would like to suggest that helicity facilitates phase separation by exposing aromatic side chains, thus facilitating their involvement in intermolecular interactions as suggested by the Huang lab[7]; indeed, a detailed analysis of the regions of sequence with some helical propensity in Tau-5 shows that they are indeed rich in aromatic residues. We have added a sentence to the Discussion to mention this possibility.

In summary, therefore, we conclude that the aromatic residues of the AD are indispensable for AR phase separation because they provide the key inter-molecular interactions that stabilize AR condensates, but that androgen binding triggers it by causing the receptor to form oligomers with increased multivalency, relative to monomeric AR, and therefore high propensity to phase separate. We would like to thank Reviewer #1 for prompting us to explain this better in the revised manuscript as we are confident this will be appreciated by the readers of *Nat. Struct. Mol. Biol.,* likely keen on mechanistic detail.

Page 7 and 8 – Supplementary Fig. 5I shows that compound 1ae was significantly more potent in LNCaP (full-length AR) compared to LNCaP95 (AR-V7) cells. The authors should discuss this and provide a more direct and quantitative comparison of potency in the activity reporter for the analogs presented in AR versus AR-V7 cell models (IC50 numbers for both, rather than bar graphs for AR-V7).

The bar graphs represented in panels E to G the original Supplementary Figure 5 were provided to show the lack of a dose-response inhibition of AR-V7 transcriptional activity by compounds 1ab (original Supplementary Fig. 5F) and 1bb (original Supplementary Fig. 5G) as measured using the V7BS3-luciferase reporter, specific to AR-V7. We were therefore unable to determine IC_{50} values for these compounds, which were among the most potent inhibitors of FL-AR (Fig. 4H). For compound 1ae, instead, we obtained the expected dose-response inhibition (original supplementary Fig. 5E), leading to an IC_{50} value of 4.1μM, which is higher than that obtained by using the PSA(6.1 kb)-luciferase, specific to full-length AR (1.54 μM, Fig. 4H): these results are therefore consistent with those shown in Supplementary Fig. 5I. To make this clearer to our readers we mention explicitly the value of IC_{50} obtained for 1ae in the relevant part of the Results section of the revised manuscript, have replaced the bar plot (original Supplementary Fig. 5E) by a dose-response inhibition curve (current Fig. 4H), and discuss that these results support that unlike enzalutamide, that targets the LBD, 1ae interacts with the AR AD, present in both isoforms.

Page 9 – the authors state that “Compound 1ae is a potent inhibitor of AR-dependent transcription and tumor growth”. While they provide data supporting a role for compound 1ae in AR-dependent transcription and tumor growth, the authors do not provide data supporting its specificity beyond the lack of activity in a non-AR-driven reporter (Supplementary Fig. 5H). It would be important to show that the anti-tumor effects of 1ae are indeed specific to AR-dependent tumor growth.

We have included to the revised manuscript gene expression data demonstrating in vivo on-target activity of 1ae for both AR-FL and AR-V7 in LNCaP and LNCaP95-D3 xenografts, respectively. While both enzalutamide and 1ae are capable of inhibiting androgen-induced genes in LNCaP xenografts, only 1ae is able to block AR-V7 mediated gene transcription in LNCaP95-D3 xenografts and, importantly, de-repressing the AR-V7 repressed gene B4GALT1[8]: the ability of compound 1ae to de-repress an AR-V7-repressed gene provides evidence that compound 1ae does generally decrease gene expression due to cytotoxicity. Importantly, in both models, neither enzalutamide nor 1ae have any effect on the housekeeping gene ALAS1. These data, represented in Supplementary Figure 8, are consistent with compound 1ae having on-target activity against AR-V7 and AR-FL.

Additionally, we would like to highlight that the RNA-seq data shown in Figure 6D also provides evidence for on-target activity of 1ae. The most significantly affected genes which are downregulated by 1ae treatment are canonical AR-regulated genes, including KLK2, KLK3, NKX3.1, TMPRSS2 and FKBP5. 1ae also has a near identical global gene signature compared to EPI-001, a validated AR-NTD antagonist with respect to the pathways which are enriched or repressed following treatment (Fig. 6E), and includes the androgen response pathway. In addition to the new Supplementary Fig. 8 we have added text describing the new results to the Results section as well as text emphasizing this to our readers in the Discussion; we thank Reviewer 1 for this request as it has led to a more compelling manuscript.

Minor comments

It would be important to clarify if native (belonging to endogenous AR) or an ectopic nuclear localization signal (NLS) was used to generate constructs for in-cell data. If an ectopic NLS was used, the relevance of the results for describing the behavior of endogenous AR and splice variants should be addressed.

The constructs generated for the in-cell data contain the endogenous NLS (^{628}RKLKK^{632}) found between the DBD and the hinge that separates it from the LBD; we have made this clear to our readers and indicated the presence of the NLS in Figure 1A.

Page 4, second paragraph – the authors use the word “sticky” to describe residues identified by NMR. The same is true in Fig. 1C. It would be helpful to better define what it is meant rather than use ambiguous language.

We acknowledge that the use of the term “sticky” may be seen as inappropriate. We took the liberty of using it by reference to the stickers-and-spacers framework put forward by Mittag and Pappu[9]. In this context this word identifies residues that are involved in interactions that

drive the collapse of the monomer when they are intramolecular – that allows their identification by NMR – and that drive condensation when they are intermolecular. We use the term neither in the main text nor in the figure of the revised manuscript.

Page 5, second paragraph – the authors state "These results collectively reveal that AR phase separation is driven by tyrosine residues within the AR AD". Based on the data presented, it would be more accurate to state that the Y residues described contribute to phase-separation of WT* AR AD. Figure 1 does not include full-length AR, nor the truly WT receptor, and does not address whether Y residues are sufficient for phase-separation. Thus, the use of "driven" seems incorrect, or at least imprecise.

Reviewer #1 is right. We have data in cells, obtained with the full-length protein devoid of the L26P mutation, allowing us to conclude this (Fig. 2A-C), but this sentence is misplaced as the relevant paragraph refers to the results reported in Figure 1. We have corrected this in the revised manuscript and thank Reviewer #1 for highlighting this error.

Page 7 – the authors study the role of helicity in in-cell phase-separation of cytoplasmic AR constructs. It is not clear why absence of DNA binding and NLS in these constructs was deemed necessary (hence their cytoplasmic localization), given that the data presented thus far address the role of nuclear AR condensates. This point should be clarified.

We acknowledge that this point merits further explanation. We studied the effect of mutations affecting helicity on the formation of cytosolic condensates because the quantitative analysis of the number and size of the condensates is greatly facilitated by cytosolic localization due, presumably, to the absence of interactions with chromatin. Based on the results obtained in vitro we anticipated that the differences due to these mutations would be modest, as shown in Fig. 2C,D, and therefore not resolvable by measuring the granularity of nuclear AR condensates. We have added an explanation of this to the revised manuscript and thank Reviewer #1 for bringing this up.

Similarly, the authors should specifically address (at least with text) the potential functional relevance of cytoplasmic versus nuclear condensates and the possible relationship between the two.

This is another point that merits further explanation. Our results indicate that decreasing the ability of the AR to condense by mutation of Tyr residues to Ser has two major functional consequences – one providing information on the role of the cytosolic condensates and another one on the role of their nuclear counterparts: it decreases the rate at which the full-length receptor translocates to the nucleus and decreases transcriptional activity. Our data indicate that the latter is due to a decrease in the ability of the mutated to form heterotypic condensates with RNA Pol II (Fig. 1G-J), establishing a functional role for the nuclear AR condensates.

Why the same mutation causes a decrease in the rate of nuclear translocation is less clear, however. The permeability barrier of nuclear pore complexes is a hydrogel formed by the intrinsically disordered regions of nucleoporin proteins (FG-Nups), that are rich in phenylalanine residues. In analogy with the results reported in Fig. 1G-J we speculate that the Tyr to Ser mutation decreases the ability of the AR AD condensates to co-phase

separate with FG-Nups; this would establish that the role of AR cytosolic condensation is to facilitate nuclear translocation. We will investigate this interesting hypothesis in future work and have added text to explain this in the Discussion part of the revised manuscript.

Page 9 – it would be important to clarify how compound 1ae decreases AR levels as revealed by Western blot analysis (Fig. 6G).

We have repeated the experiments and observed that the decrease of AR levels in the presence of cycloheximide only occurs at 1ae concentrationss higher than that leading to an inhibitory effect (Figs. 4 and 6). We conclude, therefore, that changes in AR levels are not part of the mechanism of action of 1ae, added text to clarify this and replaced Fig. 6G.

Page 10 – the authors state “These results suggest that 1ae has in vivo antitumor activity in CRPC xenograft models, and outperforms enzalutamide”. It would be important to mention that the latter point only applies to one of the two models tested.

We have corrected this in the revised manuscript and thank the reviewer for pointing this out.

There is a typo in Reporting summary / Animals and other research organisms / Reporting on sex, “make” seems incorrect.

We have corrected this in the revised manuscript and thank the reviewer for pointing this out.

<u>Reviewer #2</u>

This is a highly interesting manuscript in which the authors combine phase separation/condensation experiments in vitro and in cells with NMR-based structural/interaction analysis to better understand the cluster formation of the Androgen receptor transcription factor and then use this information to optimize a small molecule to interfere with its phase separation. Additionally, it is shown that the optimized small molecule inhibits androgen receptor-dependent transcriptional programs, and has antitumorigenic effect in models of castration-resistant prostate cancer in cells and in vivo. The data are of high quality, the manuscript is well written and nicely illustrated. Overall, this is a highly interesting and well performed study providing unique insight into transcription-associated p hase separation and small molecules targeting biomolecular condensation.

We were indeed pleased to read the positive comments that Reviewer #2 made about the work and the manuscript and are grateful for the suggestions for improvement.

I just have a few minor suggestions for improvement:

What is in the region from 450-470, which is strongly broadened? Is it important for phase separation and transcriptional activation?

The region 450-470 is a polyglycine tract. The resonances corresponding to these residues overlap and we could therefore not obtain a residue-specific assignment. We have added text to the figure caption of the revised manuscript to make this clear to our readers.

Fig. 1e: should be mM instead of uM

We have corrected this in the revised manuscript and thank the reviewer for pointing this out.

Please specify “weak” helical propensity for the two motifs 232DNAKELCKA240 and 351LDEAAAYQS359, i.e. % of helical propensity

We have added the details requested to the revised manuscript.

Reviewer #3

In this manuscript, Basu, Cristoval, and colleagues describe a compound targeting the intrinsically disordered activation domain of the androgen receptor. The compound exhibit moderate activity in vivo in a model of castration-resistant PCa (driven by AR-v7, lacking the LBD domain). The author concludes targeting the IRD is a generalizable strategy. The manuscript is interesting and well done.

We were glad to read that Reviewer #3 found the manuscript interesting and well done.

I would argue that if the authors wanted to highlight the strategy and its potential generalization, the choice of targeting AR seems a bit odd, considering the plethora of therapies developed against this target (including AR-v7). I would have expected an additional TF targeted following this strategy (something truly untargetable) since drugs against hormone receptors make up a sizable portion of all FDA-approved treatments.

This is a fair point. We would like to emphasise, however, that the goal of this work was to optimize the chemical structure of an already existing drug-like small molecule based on how the intrinsically disorder protein that it targets performs its function. Given that EPI-001 is the only such molecule that has reached clinical trials we considered that its optimization represented a challenge worth tackling. We are indeed working on using a related approach for the discovery of drugs targeting the AD of AR as well as the IDRs of other drug targets and will eventually report our results.

Furthermore, the activity in the LNCAP95 xenograft is not impressive. At a minimum, I would expect a second xenograft model mimicking a LBD-ablated AR.

The Reviewer makes another fair point. We would like to note, however, that the goal of this work was not to identify a highly potent inhibitor but, rather, to show how knowledge of how a drug-like small molecule interacts with an intrinsically disordered target and, especially, of how the disordered target performs its function, can be used to increase potency. We would like to highlight that, in response to a point raised by Reviewer #1, in the revised version we provide evidence of on-target activity *in vivo*, which emphasises further that the approach that we have used in this work is effective. In the revised version we acknowledge that the inhibitory activity of 1ae is not outstanding and explain this concept.

Reviewer #4

This work identified a sticky region in the activation domain (AD) of androgen receptor (AR) using structure studies and phase separation assays. The authors modified an existing drug to better target this sticky region and found a version that further reduces AR mediated transcription and inhibits tumor growth. This is a very nice example illustrating how phase separation can be used as drug targets. Overall, this study is well carried out.

We were pleased to read that Reviewer #4 found that our study is well carried out and represents a very nice example of how phase separation can be used for drug discovery.

My major concern is that it is not clear whether the drug disrupts/targets AR phase separation. The authors did not show how AR condensates in vitro and in cells respond to the drugs. It is possible that the drug affects other aspects of AR function other than phase separation. For example, the sticky region in the AD is important for both AR phase separation and nuclear import. To decouple these two effects, the authors did use the AR-V7 that localizes to the nucleus to measure effect of drugs on transcription. My problem with this control is that for the WT the major target can still be nuclear import. Data to show how much nuclear transport of the WT is affected by the drugs would be informative. Without these data to determine whether and how much AR phase separation is affected by the drug, the BioID and NMR data that reveal AR self-interaction and interaction with other transcription factors would be sufficient to guide the drug design and interpret the drug effect and hence there is no need for the phase separation studies.

This fair point of Reviewer #4 is largely equivalent to the main point raised by Reviewer #1, who requested experiments linking the phase separation properties of the receptor with the development of compound 1ae. In addition, Reviewer #4 explicitly requests that we show how the information about phase separation can be used to guide drug design, beyond the information gathered by techniques that probe the structure of the domain or its interactions with other proteins.

As stated in our response to Reviewer #1 we have carried out experiments that show that EPI-001 partitions in the homotypic condensates formed by the AR AD, indicating that the AR condensates are indeed the target (Fig. 4A,J,K). In addition, we have shown that the aromatic character of the AR AD condensates determines the degree of partitioning of EPI-001, linking the aromatic character of this drug-like small molecules with that of the condensates: as a consequence of that increasing the aromatic character of 1aa, to obtain 1ae, led to an increase in potency. We also show that EPI-001 reduces the AR AD cloud point (Fig. 4J) and increases droplet size (Fig. 4K) *in vitro*, more so for the more hydrophobic 1ae. Furthermore, we show how EPI-001 treatment reduces colocalization of AR with MED1 and ARD1A in nuclear foci, more so for 1ae (Fig. 5H). We trust that Reviewer #4 will agree that these experiments show how the drugs target phase separation and thus the deep understanding of this process was fundamental for drug design.

Other minor concerns:

1. Two types of AR condensates are reported: one in the cytoplasm and one in the nucleus. More discussion about similarities and difference in the formation, possible compositions, and functions of those two condensates would be useful. Does the drug disrupt one or both?

As Reviewer #4 points out, after androgen binding AR can form both cytosolic and nuclear condensates. Our experiments indicate that the aromatic character of the AR AD is key for both cytosolic (Fig. 2A) and nuclear (Fig. 2C, D) condensation indicating that AR represents the scaffolding protein in both cases. Our results also indicate that the functions of these condensates are associated with their ability to form heterotypic condensates with intrinsically disordered proteins that are rich in aromatic residues: for the cytosolic condensates these are likely FG-Nups, rich in phenylalanines, whereas for the nuclear ones this is, among others, the intrinsically disordered C-terminal tail of RNA Pol II, rich in tyrosines. From our perspective, therefore, the two types of AR condensates are fundamentally equivalent and will differ mainly in terms of the client biomolecules that partition in them as they perform their functions. Our data shows drug partitioning into purified AR AD droplets *in vitro* (Fig 4A,J,K), as well as effects in transcriptional activity (Fig. 4I and 6C) and nuclear interactions (Fig. 5B-I, Fig. 6D-G), thus indicating that the drugs might potentially target both cytoplasmic and nuclear condensates. We have added text to the discussion to introduce these ideas and thank the Reviewer for requesting that we address this fair point.

2. What is the relevant importance of the drug target region to phase separation comparing to other regions? Since other regions of AR are known to be important for its phase separation, instead of using AD and mutants for in vitro phase separation, using the full-length and mutants would be more relevant.

This is another fair point. We would like to clarify that our experiments in cells (Fig. 1A, Fig. 2), aimed at studying the functional roles of AR phase separation, were carried out with the full-length protein (and the disease-related splice variant). The results that we obtained provided us with clear evidence that the AR AD is the most relevant AR domain for phase separation in cells (Fig. 1A), which is in agreement with the fact that it readily undergoes phase separation *in vitro* (Fig. 1D). Given that this domain and, especially, the aromatic residues in Tau-5 are the target of EPI-001[10,11] we considered that working with this region of the protein was sufficient for the goal of this work, which was to learn how to improve the structure of EPI-001 to make it a more potent inhibitor. We would like to add that we tried to express and purify full-length AR in mammalian cells but were not able to overcome challenges associated with the fact that, on the one hand, inactive AR forms a tight complex with Hsp70 that we could not dissociate *in vitro* – despite the presence of androgens in the solution – and, on the other hand, that active AR formed insoluble aggregates: we have added a note to mention this in the revised manuscript.

3. Also when testing the importance of LBD and AD binding for phase separation, why not use the full-length protein and its truncations? And test direct effect of hormone binding on phase separation in vitro? This would be better than adding LBD to AD. If it’s due to technical difficulty, the authors should point out and discuss the limitations. For example, the AD shows LCST behavior, the full protein may not.

We agree with the reviewer that it would have been better to work with the full-length protein *in vitro*, but the challenges listed in our response to the previous point prevented us from doing so. We would nevertheless like to note that our general approach is to carry out experiments *in vitro*, with the inevitable simplifications that this involves, and then validate the results in relevant cells with the full-length protein and under solution conditions

resembling the physiological ones. Also for the investigation of the impact of the N/C interaction on AR phase separation, specifically mentioned by the Reviewer, we would like to note that to respond to a point of Reviewer #1 we repeated our *in vitro* experiments with a construct that is exactly equivalent to that used in cells, concluding that this interaction is not indispensable for AR separation but contributes to driving it.

4. Figure 1G, RNAPII-CTD droplets are smaller for the 22YtoS mutant. Is that representative? In other words, does AD passively partition or actively affect RNAPII-CTD phase behavior? This information would be useful for understanding the nature of AR condensates at transcription sites.

AD passively partitions in the RNAPII-CTD condensates but mutation of the tyrosine residues to serines significantly decreases the partitioning coefficient (Fig. 1H). We attribute the increase in the size of the RNAPII-CTD condensates to a stabilization of the RNAPII-CTD condensates by AD. The small size of the RNAPII-CTD condensates in the presence of the 22YtoS is related to this effect.

—-

To end our response we would like to thank all four Reviewers for their help in improving our manuscript and hope that they will consider that it is now suitable for publication in *Nat Struct Mol Biol.*

References

1. He, B. *et al.* Structural basis for androgen receptor interdomain and coactivator interactions suggests a transition in nuclear receptor activation function dominance. *Mol. Cell* **16**, 425–438 (2004).
2. van Royen, M. E., van Cappellen, W. A., de Vos, C., Houtsmuller, A. B. & Trapman, J. Stepwise androgen receptor dimerization. *J. Cell Sci.* **125**, 1970–1979 (2012).
3. Nadal, M. *et al.* Structure of the homodimeric androgen receptor ligand-binding domain. *Nat. Commun.* **8**, 14388 (2017).
4. Martin, E. W. & Mittag, T. Relationship of Sequence and Phase Separation in Protein Low-Complexity Regions. *Biochemistry* **57**, 2478–2487 (2018).
5. Dignon, G. L., Zheng, W., Kim, Y. C., Best, R. B. & Mittal, J. Sequence determinants of protein phase behavior from a coarse-grained model. *PLoS Comput. Biol.* **14**, e1005941 (2018).
6. Shin, Y. *et al.* Spatiotemporal Control of Intracellular Phase Transitions Using

Light-Activated optoDroplets. *Cell* vol. 168 159–171.e14 (2017).

7. Li, H.-R., Chiang, W.-C., Chou, P.-C., Wang, W.-J. & Huang, J.-R. TAR DNA-binding protein 43 (TDP-43) liquid-liquid phase separation is mediated by just a few aromatic residues. *J. Biol. Chem.* **293**, 6090–6098 (2018).
8. Cato, L. *et al.* ARv7 Represses Tumor-Suppressor Genes in Castration-Resistant Prostate Cancer. *Cancer Cell* **35**, 401–413.e6 (2019).
9. Martin, E. W. *et al.* Valence and patterning of aromatic residues determine the phase behavior of prion-like domains. *Science* **367**, 694–699 (2020).
10. De Mol, E. *et al.* EPI-001, A Compound Active against Castration-Resistant Prostate Cancer, Targets Transactivation Unit 5 of the Androgen Receptor. *ACS Chem. Biol.* **11**, 2499–2505 (2016).
11. Zhu, J., Salvatella, X. & Robustelli, P. Small molecules targeting the disordered transactivation domain of the androgen receptor induce the formation of collapsed helical states. *Nat. Commun.* **13**, 6390 (2022).

Decision Letter, first revision:

Message: 26th Jun 2023

Dear Dr. Salvatella,

Thank you again for submitting your manuscript "Rational optimization of a transcription factor activation domain inhibitor". We now have comments (below) from the 4 reviewers who evaluated your paper. In light of those reports, we remain interested in your study and would like to see your response to the comments of the referees, in the form of a revised manuscript.

You will see that while the reviewers highly appreciate the improvements made in the last round of revision, Reviewers #1 and #4 have outstanding requests to solidify the link between the phase separation aspects of the study with the activity of the AR-targeting compounds. Reviewer #4 requests to interpret the AR phase separation observations in terms of the mechanism of action of the compounds.
Although we would editorially be willing to overrule on the concerns of Reviewer #1 concerning expanding on the panel of tested compounds, we would like to see the effect of the two compounds EPI-001 and 1ae on AR condensation in cells tested - a concern echoed by both Reviewer #1 and Reviewer #4 - to allow a more meaningful interpretation of the relationship between the activity of the compounds and the phase separation of AR. Editorially, we agree with the two reviewers that this would substantially strengthen the manuscript. We would also like to see the remaining points of Reviewer #1 addressed textually to expound the limits of interpretability of the selectivity and partitioning of the compounds tested at the present time. Please be sure to address/respond to all concerns of the referees in full in a point-by-point response and highlight all changes in the revised manuscript text file. If you have comments that are intended for editors only, please include those in a separate cover letter. Please don't hesitate to reach out to me if you wish to discuss these reviewer reports further.

We are committed to providing a fair and constructive peer-review process. Do not hesitate to contact us if there are specific requests from the reviewers that you believe are technically impossible or unlikely to yield a meaningful outcome.

We expect to see your revised manuscript within 6 weeks. If you cannot send it within this time, please contact us to discuss an extension; we would still consider your revision, provided that no similar work has been accepted for publication at NSMB or published elsewhere.

As you already know, we put great emphasis on ensuring that the methods and statistics reported in our papers are correct and accurate. As such, if there are any changes that should be reported, please submit an updated version of the Reporting Summary along with your revision.

Please follow the links below to download these files:

Reporting Summary:
https://www.nature.com/documents/nr-reporting-summary.pdf

Please note that the form is a dynamic ‘smart pdf’ and must therefore be downloaded and completed in Adobe Reader.

When submitting the revised version of your manuscript, please pay close attention to our href="https://www.nature.com/nature-portfolio/editorial-policies/image-integrity">Digital Image Integrity Guidelines. and to the following points below:

-- that unprocessed scans are clearly labelled and match the gels and western blots presented in figures.
-- that control panels for gels and western blots are appropriately described as loading on sample processing controls
-- all images in the paper are checked for duplication of panels and for splicing of gel lanes.

Finally, please ensure that you retain unprocessed data and metadata files after publication, ideally archiving data in perpetuity, as these may be requested during the peer review and production process or after publication if any issues arise.

Please note that all key data shown in the main figures as cropped gels or blots should be presented in uncropped form, with molecular weight markers. These data can be aggregated into a single supplementary figure item. While these data can be displayed in a relatively informal style, they must refer back to the relevant figures. These data should be submitted with the final revision, as source data, prior to acceptance, but you may want to start putting it together at this point.

SOURCE DATA: we request that authors provide, in tabular form, the data underlying the graphical representations used in figures. This is to further increase transparency in data reporting, as detailed in this editorial (http://www.nature.com/nsmb/journal/v22/n10/full/nsmb.3110.html). Spreadsheets can be submitted in excel format. Only one (1) file per figure is permitted; thus, for multi-paneled figures, the source data for each panel should be clearly labeled in the Excel file; alternately the data can be provided as multiple, clearly labeled sheets in an Excel file. When submitting files, the title field should indicate which figure the source data pertains to. We encourage our authors to provide source data at the revision stage, so that they are part of the peer-review process.

Data availability: this journal strongly supports public availability of data. All data used in accepted papers should be available via a public data repository, or alternatively, as Supplementary Information. If data can only be shared on request, please explain why in your Data Availability Statement, and also in the correspondence with your editor. Please note that for some data types, deposition in a public repository is mandatory - more information on our data deposition policies and available repositories can be found below: https://www.nature.com/nature-research/editorial-policies/reporting-standards#availability-of-data

We require deposition of coordinates (and, in the case of crystal structures, structure factors) into the Protein Data Bank with the designation of immediate release upon publication (HPUB). Electron microscopy-derived density maps and coordinate data must

be deposited in EMDB and released upon publication. Deposition and immediate release of NMR chemical shift assignments are highly encouraged. Deposition of deep sequencing and microarray data is mandatory, and the datasets must be released prior to or upon publication. To avoid delays in publication, dataset accession numbers must be supplied with the final accepted manuscript and appropriate release dates must be indicated at the galley proof stage.

While we encourage the use of color in preparing figures, please note that this will incur a charge to partially defray the cost of printing. Information about color charges can be found at http://www.nature.com/nsmb/authors/submit/index.html#costs

Nature Structural & Molecular Biology is committed to improving transparency in authorship. As part of our efforts in this direction, we are now requesting that all authors identified as 'corresponding author' on published papers create and link their Open Researcher and Contributor Identifier (ORCID) with their account on the Manuscript Tracking System (MTS), prior to acceptance. This applies to primary research papers only. ORCID helps the scientific community achieve unambiguous attribution of all scholarly contributions. You can create and link your ORCID from the home page of the MTS by clicking on 'Modify my Springer Nature account'. For more information please visit please visit www.springernature.com/orcid.

Please use the link below to submit your revised manuscript and related files:

[redacted]

Note: This URL links to your confidential home page and associated information about manuscripts you may have submitted, or that you are reviewing for us. If you wish to forward this email to co-authors, please delete the link to your homepage.

We look forward to seeing the revised manuscript and thank you for the opportunity to review your work.

Sincerely,
Sara

Sara Osman, Ph.D.
Associate Editor
Nature Structural & Molecular Biology

Referee expertise:

Reviewers' Comments:

Reviewer #1:
Remarks to the Author:
Thank you for revising the manuscript and addressing many of the original comments. The manuscript has improved and the link between condensates and small molecule optimization is now clearer. However, some questions remain regarding the link between compounds' in-cell / anti-tumor effects and the modulation of AR condensates.

The authors report the partition coefficient of EPI-001 (Fig. 4A in the revised manuscript and associated text). Was this measured for the optimized compounds also, and compound 1ae in particular? Is partitioning selective for AR condensates?

It would be helpful to build a more complete link between compounds' effect on reconstituted condensates (in a test tube) and their activity in cells by subjecting a larger panel of compounds discussed in the manuscript to cloud point and droplet size assays (only EPI-001 and compound 1ae were tested), and partitioning assays. Is there a quantitative relationship between condensate modulation and in-cell activity?

Moreover, it would be helpful to measure the effect of the same compound panel (or minimally of EPI-001 and compound 1ae) on cellular AR condensates, in intact cells, in assays like those used to study AR mutants. This would address more directly condensate-specific effects and complement the BioID-MS analysis reported.

Minor points

The authors state at the end of the abstract "These results show that it is possible to rationally design small molecules that target the activation domains of oncogenic transcription factors." However, as stated two sentences above, compound 1ae was optimized from a molecule obtained from a phenotypic screen. For this reason, it would be more appropriate to use "rationally optimize" rather than "rationally design", which may imply design of a new molecule from nothing.

When discussing compound partitioning in AR-AD droplets, (PWT EPI-001 ≈32) should read "P8YtoS" or equivalent, as it refers to the mutant.

Readers would benefit from explanations or definitions for "oligomer" versus "aggregate" versus "condensate", also given the rationale for using the L26P mutant.

Reviewer #2:
Remarks to the Author:
The authors have fully addressed my suggestions in the revised version of the manuscript.

Reviewer #3:
Remarks to the Author:
I don't have any additional comments

Reviewer #4:
Remarks to the Author:
The authors have addressed most of my concerns except one: the relevance of AR phase separation in the drug design. According to the new data, EPI001 and more so 1ae reduce cloud point and increase AR droplet size. What does this mean? The drug works by promoting AR phase separation? Is this conserved in cells, i.e., how does the drug affect

the cytoplasmic and nuclear AR condensates? Does the optimized 1ae affect them more than EPI001? The authors stated: "after androgen binding AR can form both cytosolic and nuclear condensates. Our experiments indicate that the aromatic character of the AR AD is key for both cytosolic (Fig. 2A) and nuclear (Fig. 2C, D) condensation indicating that AR represents
the scaffolding protein in both cases. Our results also indicate that the functions of these condensates are associated with their ability to form heterotypic condensates with intrinsically disordered proteins that are rich in aromatic residues: for the cytosolic condensates these are likely FG-Nups, rich in phenylalanines, whereas for the nuclear ones
this is, among others, the intrinsically disordered C-terminal tail of RNA Pol II, rich in tyrosines. From our perspective, therefore, the two types of AR condensates are fundamentally equivalent and will differ mainly in terms of the client biomolecules that partition in them as they perform their functions". If the aromatic residues are important for homotypic phase separation and heterotypic interaction with clients in the two condensates, how come the drug increase AR phase separation while reduce interaction with transcription factors?

Author Rebuttal, first revision:

Reviewers' Comments:

Reviewer #1:

Remarks to the Author:
Thank you for revising the manuscript and addressing many of the original comments. The manuscript has improved and the link between condensates and small molecule optimization is now clearer. However, some questions remain regarding the link between compounds' in-cell / anti-tumor effects and the modulation of AR condensates.

We would like to thank the reviewer for the useful comments throughout the review process, and for noting the improvements. We address all the outstanding questions below.

The authors report the partition coefficient of EPI-001 (Fig. 4A in the revised manuscript and associated text). Was this measured for the optimized compounds also, and compound 1ae in particular? Is partitioning selective for AR condensates?

We measured the partition coefficient of optimized compounds in AR condensates *in vitro*. The optimized compounds tend to have poor solubility, which poses a significant problem in these assays, and results in large variance between measurements. Therefore, we prefer not to include these data, and work out the technical challenges in future work.

It would be helpful to build a more complete link between compounds' effect on reconstituted condensates (in a test tube) and their activity in cells by subjecting a larger panel of compounds discussed in the manuscript to cloud point and droplet size assays (only EPI-001 and compound 1ae were tested), and partitioning assays. Is there a quantitative relationship between condensate modulation and in-cell activity?

We agree that a more complete link would be helpful. The challenges with testing the various compounds in *in vitro* assays are described above. We note that there is a correlation between the hydrophobicity and inhibitory effect of the compounds in luciferase assays (Fig. 4H).

Our model on the mode of action of the compounds summarized as follows: when the AR partitions into condensates, sequences that are prone to form helices assume transiently stable helical conformations. The aromatic residues and the transient binding pocket formed by the helices in the Tau 5 region facilitate the partitioning of the compounds into the AR condensates. The interaction of the compound with the AR is stabilized by the transient pocket and further stabilized by covalent attachment to cysteine residues. The compound interaction traps the AR in a conformation that is less potent in interacting with transcriptional co-factors, and leads to an inhibition of AR-dependent transcriptional programs. This model is consistent with the *in vitro* cloud point, mutagenesis, RNA-Seq, Bio-ID, Mass-Spec and Xenograft assays. We stress that this model postulates that *compound partitioning into AR condensates* is key for the mechanism of action. *This does not necessarily mean that the compounds impact AR condensation in cells*. We have clarified the model in a newly added paragraph in the Discussion.

We appreciate that investigating the effect of the compounds on AR condensates is an interesting question, with noting that it is not an essential part of the proposed model. Imaging AR condensates is technically challenging, because they are small and dynamic. The requested measurements in cells are on the technical limits of current super-resolution imaging. We used three independent cellular systems to perform such measurements. 1) imaging endogenous AR condensates in DMSO-treated and 1ae-treated LnCAP prostate cancer cells using antibody staining against the AR, 2) generating a transgenic HeLa cell line that encodes AR-GFP transgenes, and treating the cell line with DMSO and 1ae, and imaging AR-GFP in fixed cells with an anti-GFP antibody, and 3) imaging HEK293T cell lines transiently transfected with AR-GFP and treated with either DMSO or 1ae. We used STED super-resolution imaging to characterize the number, size and signal intensity of AR condensates in the three systems (**Reviewer Figure 1**, below). In brief we found the following:

1. Detecting AR condensates is challenging, because of their small size and large number. Even with STED imaging, the number of pixels that constitute an AR condensate is small, leading to limited range of signal. See Panels A and B in reviewer Figure 1 below. Both in the HeLa AR-GFP system (top), and the LnCAP cells (bottom), condensates are clearly detectable, are nevertheless small, and challenging to identify algorithmically.

2. Quantifying the number, size and mean intensity of signal of AR condensates did not reveal robustly quantifiable differences between 1ae-treadted and DMSO treated LnCAP cells (panel C), HeLa AR-GFP cells (panel D) or HEK293T cells transiently transfected with AR-GFP expression vector (panel E).

We prefer not to include these data in the manuscript for two main reasons. First, as detailed above, our model on the mechanism of action of the compounds is agnostic to any effect on AR condensation in cells. Second, in the systems described above, the absence of large, robustly quantifiable differences of AR condensates between DMSO and 1ae-treated cells may be caused by limitations of the detection, and tools for confirming partitioning of compounds into AR condensates in cells are lacking.

Moreover, it would be helpful to measure the effect of the same compound panel (or minimally of EPI-001 and compound 1ae) on cellular AR condensates, in intact cells, in assays like those used to study AR mutants. This would address more directly condensate-specific effects and complement the BioID-MS analysis reported.

The response to this point is included in the response to the point above.

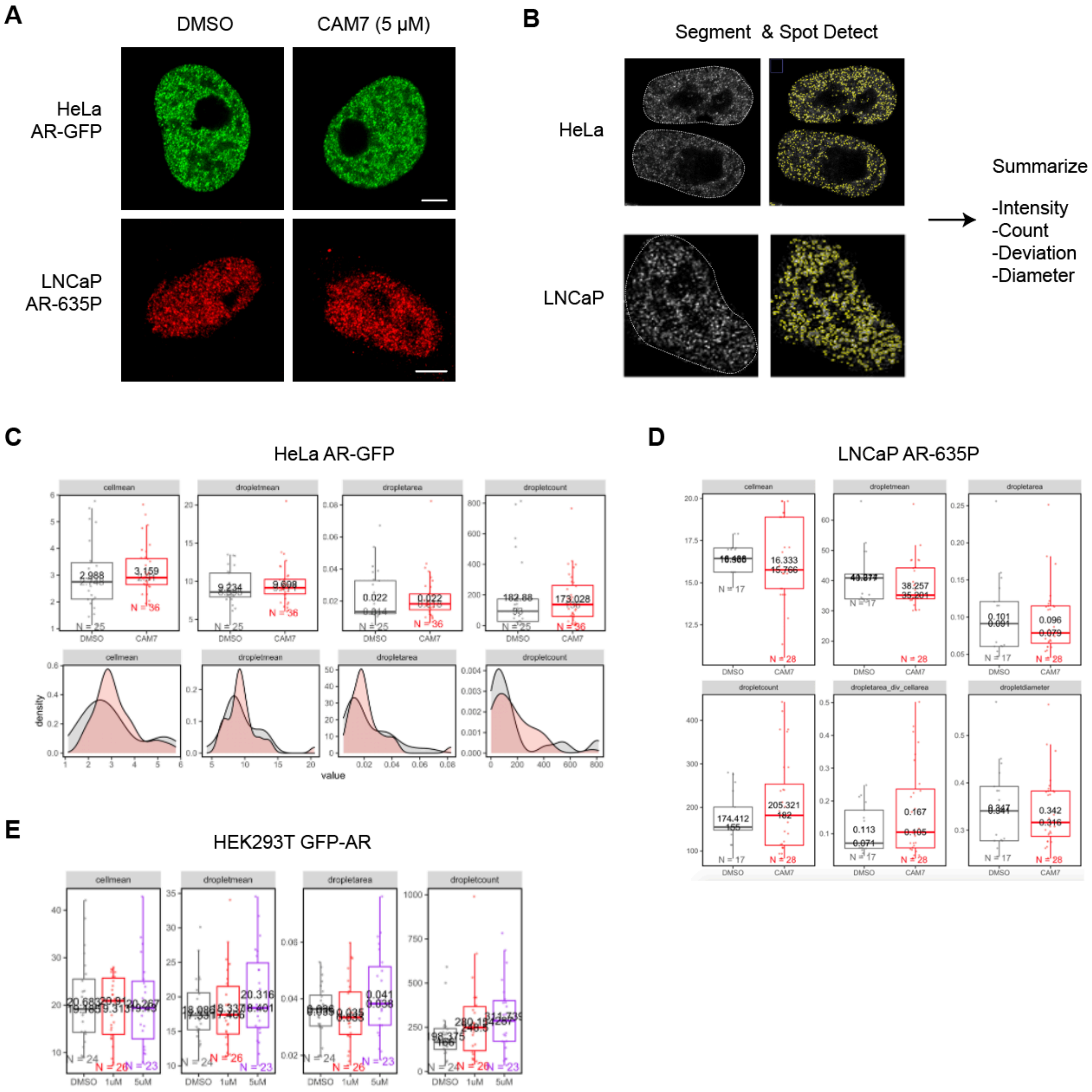

Reviewer Figure 1. A.) STED images of the nuclei of DMSO and 1ae-treated HeLa AR-GFP cells with a GFP antibody (top), and LnCAP prostate cancer cells with an AR antibody. **B.)** Scheme of the segmentation and spot detection algorithm. **C-E.)** Quantification of the indicated condensate features in the three cell systems imaged with STED.

Minor points

The authors state at the end of the abstract “These results show that it is possible to rationally design small molecules that target the activation domains of oncogenic transcription factors.” However, as stated two sentences above, compound 1ae was optimized from a molecule obtained from a phenotypic screen. For this reason, it would be more appropriate to use “rationally optimize” rather than “rationally design”, which may imply design of a new molecule from nothing.

We revised the sentence in the abstract to read: “These results suggest that it is possible to rationally optimise, and potentially even to design small molecules that target the activation domains of oncogenic transcription factors.”

When discussing compound partitioning in AR-AD droplets, (PWT EPI-001 ≈32) should read “P8YtoS” or equivalent, as it refers to the mutant.

Fixed. Thank you!

Readers would benefit from explanations or definitions for “oligomer” versus “aggregate” versus “condensate”, also given the rationale for using the L26P mutant.

We define the term oligomer and aggregate in the methods section.

Reviewer #2:

Remarks to the Author:
The authors have fully addressed my suggestions in the revised version of the manuscript.

Reviewer #3:

Remarks to the Author:
I don't have any additional comments

Reviewer #4:

Remarks to the Author:
The authors have addressed most of my concerns except one: the relevance of AR phase separation in the drug design. According to the new data, EPI001 and more so 1ae reduce cloud point and increase AR droplet size. What does this mean? The drug works by promoting AR phase separation? Is this conserved in cells, i.e., how does the drug affect the cytoplasmic and

nuclear AR condensates? Does the optimized 1ae affect them more than EPI001? The authors stated: “after androgen binding AR can form both cytosolic and nuclear condensates. Our experiments indicate that the aromatic character of the AR AD is key for both cytosolic (Fig. 2A) and nuclear (Fig. 2C, D) condensation indicating that AR represents the scaffolding protein in both cases. Our results also indicate that the functions of these condensates are associated with their ability to form heterotypic condensates with intrinsically disordered proteins that are rich in aromatic residues: for the cytosolic condensates these are likely FG-Nups, rich in phenylalanines, whereas for the nuclear ones this is, among others, the intrinsically disordered C-terminal tail of RNA Pol II, rich in tyrosines. From our perspective, therefore, the two types of AR condensates are fundamentally equivalent and will differ mainly in terms of the client biomolecules that partition in them as they perform their functions”. If the aromatic residues are important for homotypic phase separation and heterotypic interaction with clients in the two condensates, how come the drug increase AR phase separation while reduce interaction with transcription factors?

We thank the reviewer for the comment, which prompted us to further clarify the link between AR phase separation and compound optimisation.

Our model on the mode of action of the compounds summarized as follows: when the AR partitions into condensates, sequences that are prone to form helices assume transiently stable helical conformation. The aromatic residues and the transient binding pocket formed by the helices in the Tau 5 region facilitate the partitioning of the compounds into the AR condensates. The interaction of the compound with the AR is stabilized by the transient pocket and further stabilized by covalent attachment to nearby cysteines. The compound interaction traps the AR in a conformation that is less potent in interacting with transcriptional co-factors, and leads to an inhibition of AR-dependent transcriptional programs. This model is consistent with the *in vitro* cloud point, mutagenesis, RNA-Seq, Bio-ID, Mass-Spec and Xenograft assays. We stress that this model postulates that compound partitioning into AR condensates is key for the mechanism of action. This does not necessarily mean that the compounds impact AR condensation in cells. We have clarified the model in a newly added paragraph in the Discussion.

The results and comments on imaging AR condensates in compound-treated cells is described in detail at our response the major comment 2 of Reviewer 1 above. *In vitro*, the compounds indeed appear to enhance partitioning of AR AD protein into condensates, as described above.

Decision Letter, second revision:

Message: Our ref: NSMB-A47068B

13th Jul 2023

Dear Dr. Salvatella,

Thank you for submitting your revised manuscript "Rational optimization of a transcription factor activation domain inhibitor" (NSMB-A47068B). We have now editorially assessed the manuscript and the point-by-point response letter to the reviewers' remaining concerns and find that the manuscript has improved in revision, and we are editorially satisfied with the responses, and therefore we'll be happy in principle to publish it in Nature Structural & Molecular Biology, pending minor revisions to comply with our editorial and formatting guidelines.

We are now performing detailed checks on your paper and will send you a checklist detailing our editorial and formatting requirements in the next couple of weeks. Please do not upload the final materials and make any revisions until you receive this additional information from us.

To facilitate our work at this stage, it is important that we have a copy of the main text as a word file. If you could please send along a word version of this file as soon as possible, we would greatly appreciate it; please make sure to copy the NSMB account (cc'ed above).

Thank you again for your interest in Nature Structural & Molecular Biology Please do not hesitate to contact me if you have any questions.

Sincerely,
Sara

Sara Osman, Ph.D.
Associate Editor
Nature Structural & Molecular Biology

Final Decision Letter:

Message: 23rd Oct 2023

Dear Dr. Salvatella,

We are now happy to accept your revised paper "Rational optimization of a transcription factor activation domain inhibitor" for publication as a Article in Nature Structural & Molecular Biology.

Acceptance is conditional on the manuscript's not being published elsewhere and on there being no announcement of this work to the newspapers, magazines, radio or television until the publication date in Nature Structural & Molecular Biology.

Over the next few weeks, your paper will be copyedited to ensure that it conforms to Nature Structural & Molecular Biology style. Once your paper is typeset, you will receive an email with a link to choose the appropriate publishing options for your paper and our Author Services team will be in touch regarding any additional information that may be required.

After the grant of rights is completed, you will receive a link to your electronic proof via email with a request to make any corrections within 48 hours. If, when you receive your proof, you cannot meet this deadline, please inform us at rjsproduction@springernature.com immediately.

You will not receive your proofs until the publishing agreement has been received through our system.

Due to the importance of these deadlines, we ask that you please let us know now whether you will be difficult to contact over the next month. If this is the case, we ask you provide us with the contact information (email, phone and fax) of someone who will be able to check the proofs on your behalf, and who will be available to address any last-minute problems.

To assist our authors in disseminating their research to the broader community, our SharedIt initiative provides all co-authors with the ability to generate a unique shareable link that will allow anyone (with or without a subscription) to read the published article. Recipients of the link with a subscription will also be able to download and print the PDF.

As soon as your article is published, you can generate your shareable link by entering the DOI of your article here: <a href="http://authors.springernature.com/share">http://authors.springernature.com/share <a>. Corresponding authors will also receive an automated email with the shareable link

Note the policy of the journal on data deposition: http://www.nature.com/authors/policies/availability.html.

Your paper will be published online soon after we receive proof corrections and will appear in print in the next available issue. You can find out your date of online publication by contacting the production team shortly after sending your proof corrections. Content is published online weekly on Mondays and Thursdays, and the embargo is set at 16:00 London time (GMT)/11:00 am US Eastern time (EST) on the day of publication. Now is the time to inform your Public Relations or Press Office about your paper, as they might be interested in promoting its publication. This will allow them time to prepare an accurate and satisfactory press release. Include your manuscript tracking number (NSMB-A47068C) and our journal name, which they will need when they contact our press office.

About one week before your paper is published online, we shall be distributing a press release to news organizations worldwide, which may very well include details of your work. We are happy for your institution or funding agency to prepare its own press release, but it must mention the embargo date and Nature Structural & Molecular Biology. If you or your

Press Office have any enquiries in the meantime, please contact press@nature.com.

You can now use a single sign-on for all your accounts, view the status of all your manuscript submissions and reviews, access usage statistics for your published articles and download a record of your refereeing activity for the Nature journals.

If you have not already done so, we strongly recommend that you upload the step-by-step protocols used in this manuscript to the Protocol Exchange. Protocol Exchange is an open online resource that allows researchers to share their detailed experimental know-how. All uploaded protocols are made freely available, assigned DOIs for ease of citation and fully searchable through nature.com. Protocols can be linked to any publications in which they are used and will be linked to from your article. You can also establish a dedicated page to collect all your lab Protocols. By uploading your Protocols to Protocol Exchange, you are enabling researchers to more readily reproduce or adapt the methodology you use, as well as increasing the visibility of your protocols and papers. Upload your Protocols at www.nature.com/protocolexchange/. Further information can be found at www.nature.com/protocolexchange/about.

An online order form for reprints of your paper is available at https://www.nature.com/reprints/author-reprints.html. Please let your coauthors and your institutions' public affairs office know that they are also welcome to order reprints by this method.

Please note that <i>Nature Structural & Molecular Biology</i> is a Transformative Journal (TJ). Authors may publish their research with us through the traditional subscription access route or make their paper immediately open access through payment of an article-processing charge (APC). Authors will not be required to make a final decision about access to their article until it has been accepted. Find out more about Transformative Journals

Authors may need to take specific actions to achieve compliance with funder and institutional open access mandates. If your research is supported by a funder that requires immediate open access (e.g. according to Plan S principles) then you should select the gold OA route, and we will direct you to the compliant route where possible. For authors selecting the subscription publication route, the journal’s standard licensing terms will need to be accepted, including self-archiving policies. Those licensing terms will supersede any other terms that the author or any third party may assert apply to any version of the manuscript.

In approximately 10 business days you will receive an email with a link to choose the appropriate publishing options for your paper and our Author Services team will be in touch regarding any additional information that may be required.

You will not receive your proofs until the publishing agreement has been received through our system.

If you have any questions about our publishing options, costs, Open Access requirements, or our legal forms, please contact ASJournals@springernature.com

Sincerely,
Sara

Sara Osman, Ph.D.
Associate Editor
Nature Structural & Molecular Biology
